# Identity state‐dependent self‐relevance and emotional intensity ratings of words in dissociative identity disorder: A controlled longitudinal study

**DOI:** 10.1002/brb3.3208

**Published:** 2023-09-18

**Authors:** Aikaterini I. Strouza, Andrew J. Lawrence, Eline M. Vissia, Andreana Kakouris, Ayse Akan, Ellert R. S. Nijenhuis, Nel Draijer, Sima Chalavi, Antje A. T. S. Reinders

**Affiliations:** ^1^ Department of Psychological Medicine, Institute of Psychiatry, Psychology & Neuroscience King's College London London UK; ^2^ Department of Psychiatry, Amsterdam UMC, Location VUmc VU University Amsterdam Amsterdam The Netherlands; ^3^ Heelzorg Centre for Psychotrauma Zwolle The Netherlands; ^4^ Department of Psychosis Studies, Institute of Psychiatry King's College London London UK; ^5^ North East London NHS Foundation Trust London UK; ^6^ Clienia Littenheid AG Private Clinic for Psychiatry and Psychotherapy Littenheid Switzerland; ^7^ Department of Psychiatry VU University Medical Center Amsterdam The Netherlands; ^8^ Movement Control and Neuroplasticity Research Group, Department of Movement Sciences KU Leuven Leuven Belgium

**Keywords:** dissociation, posttraumatic stress disorder, trauma

## Abstract

**Introduction:**

Dissociative identity disorder (DID) is characterized by, among others, amnesic episodes and the recurrence of different dissociative identity states. While consistently observed in clinical settings, to our knowledge, no controlled research study has shown the degree to which different identity states report autobiographical knowledge over time. Hence, the current study investigates self‐relevance and emotional intensity ratings of words longitudinally.

**Methods:**

Data of 46 participants were included: 13 individuals with DID, 11 DID‐simulating actors, and a control group of 22 paired individuals. Individuals with DID and DID simulators participated once in the neutral identity state (NIS) and once in the trauma‐related dissociative identity state (TIS). The control group paired 11 healthy controls with 11 participants with posttraumatic stress disorder (PTSD) as a NIS–TIS pair. Self‐relevance ratings of different word types were collected in a baseline and a follow‐up session, on average 6 weeks apart. A mixed ANOVA design was used to assess the effects of group, session, word type, and dissociative identity state.

**Results:**

All participants in TIS and individuals with DID in NIS rated self‐relevant trauma‐related words more negatively. In the NIS, the control group rated self‐relevant trauma‐related words as less negative, whereas the ratings of simulating actors were intermediate. There was no group‐dependent longitudinal effect for intensity ratings.

**Conclusions:**

This study was the first to confirm clinical observations that self‐relevant and emotional processing are different between individuals with DID and controls, but consistent over time. Actors were unable to perfectly simulate DID. The finding that ratings of self‐relevant trauma‐related words differ between subgroups as included in the study is in line with clinical observations.

## Graphical abstract

Self‐relevant and emotional processing was identity state dependent and different between individuals with dissociative identity disorder and two control groups, but consistent over time. Participating actors were not able to perfectly simulate the dissociative identity disorder.

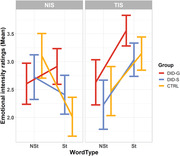

## 1 INTRODUCTION

Dissociative identity disorder (DID) is a psychiatric disorder characterized by recurrent activation of two or more distinct dissociative identity states, episodes of dissociative amnesia, and various other dissociative symptoms (APA, 2013). Although disrupted memory function in DID is considered a core characteristic and has been studied cross‐sectionally, longitudinal research of memory functioning in DID is lacking. A recent study suggests that individuals with DID in either adult or child dissociative identity states do not always experience a consistent sense of self over time (Dorahy et al., 2021). A longitudinal study into the consistency of self‐relevance and emotional intensity of word ratings can be considered a next step in the study of the identity state‐dependent sense of self in individuals with DID. However, most cross‐sectional studies have used standardized lists of general neutral or emotionally valenced words rather than subject‐specific and trauma‐related words (for review, see Reinders et al., 2022). The present study aims to investigate self‐relevance and emotional intensity ratings of individualized words over time by individuals with DID and two control groups.

Different prototypical dissociative identity states (Nijenhuis, 2015) have been referred to as trauma‐related identity states (TIS) and neutral identity states (NIS), respectively (Reinders et al., 2012; Vissia et al., 2016). These dissociative identity states recurrently take control of the individual's behavior and consciousness affecting autobiographical recall (Chiu et al., 2012; Reinders et al., 2012). As an NIS, individuals with DID long and strive to function in daily life as “normal” as possible. They achieve this aim in part by mentally avoiding trauma‐related knowledge while reporting partial or complete amnesia (Boysen & Vanbergen, 2013; Reinders et al., 2003, 2012, 2016). In contrast, as a TIS, individuals often present with child‐like behavior and sense of age, are disoriented in place and time, and are more likely to recollect autobiographical trauma‐related experiences (Dorahy et al., 2021; Reinders et al., 2006).

The majority of studies investigating memory performance in DID have focused on non‐autobiographical knowledge transfer between dissociative identity states, that is, inter‐identity amnesia (for review, see Reinders et al., 2022). The studies including self‐relevant cues (Huntjens et al., 2012, 2014, 2016; Marsh et al., 2018; Reinders et al., 2003, 2006, 2014, 2012, 2016) were cross‐sectional, and it remains unknown how consistent words are rated in terms of self‐relevance and valence at different points in time.

The current study aims to compare self‐relevance intensity ratings and emotional intensity ratings of words in two dissociative identity states at two points in time between three groups, that is, individuals with a diagnosis of DID, actors simulating DID, and a carefully paired control group of healthy participants and individuals with a diagnosis of posttraumatic stress disorder (PTSD). Our null hypothesis is that the intensity ratings of words did not differ between groups at the two points in time. Three types of a priori rated identity state‐dependent words were included: self‐relevant trauma‐related (St), non‐self‐relevant trauma‐related (NSt), and non‐self‐relevant neutral (NSn). We hypothesize that at both time points, both dissociative identity states of the three groups rate St items as more self‐relevant than NSt items, rate trauma‐related words (St, NSt) as more emotionally intense than neutral words (NSn), but an effect of group and identity state for self‐relevance and emotional intensity exists such that individuals diagnosed with DID (genuine DID, DID‐G) show a different pattern of reactions relative to each of the two control groups. More specifically, we expect DID‐G to show highest ratings of self‐relevance for the self‐relevant trauma‐related words (St) and higher emotional intensity ratings for the trauma‐related words (St, NSt) so that the DID‐simulating controls (DID‐S) will not, or only partially, simulate DID's reactions and the paired control group will be most different from the DID‐G group; these ratings will be identity state dependent and highest in the DID‐G TIS state.

## 2 METHODS

### 2.1 Participants

The present study is part of the Dutch Neuroimaging Dissociative Identity Disorder project that included the study of autobiographical memory. Participants information has been described in detail (Chalavi, Vissia, Giesen, Nijenhuis, Draijer, Barker, et al., 2015; Chalavi, Vissia, Giesen, Nijenhuis, Draijer, Cole, et al., 2015; Dimitrova et al., 2022; Vissia et al., 2016).

In sum, individuals with diagnosed DID (DID‐G; for definitions of all key abbreviations, refer to Appendix A in the Supporting Information) were recruited from various psychiatric care settings in the Netherlands or through online advertisements. Two independent DID experts, Dr Ellert R. S. Nijenhuis and Dr Nel Draijer, confirmed the participants’ diagnosis of DID using the Dutch edition of the Structural Clinical Interview for DSM‐IV Dissociative Disorders (Boon & Draijer, 1993; Steinberg, 1993) and their ability to sufficiently control and switch between their neutral and trauma‐related identity states. Participants for this study were carefully selected based on having these two different identity states. Of note, whether within an individual with DID all of the dissociative identity states can firmly and unequivocally be assigned to either a neutral or trauma‐related category needs to be clinically and empirically confirmed. The identity state criteria for participants with diagnosed DID are provided in Appendix B in the Supporting Information.

The participants who simulated DID (DID‐S) were actors with a minimum of 2 years of acting experience recruited through advertisements and included to address etiology concerns (Reinders & Veltman, 2021). Inclusion criteria included no diagnosis of any psychiatric disorders and no past psychiatric medication. They were prescreened to closely match the DID‐G individuals for age, gender, and years of education. The actors were required to simulate genuine DID and enact two different dissociative identity states, one NIS and one TIS of the DID‐G group.

DID and PTSD are both trauma‐related and highly comorbid disorders, suggesting a close relationship between them (Vissia et al., 2016). Therefore, individuals with PTSD were carefully selected as controls for the trauma aware identity state, that is, the TIS of participants with genuine DID. Additionally, non‐simulating study‐blind healthy participants were included as an analogue of the NIS for the diagnosed DID group (Vissia et al., 2016). Thus, a paired between‐subject control group was formed to be comparable to the TIS and NIS within‐subject identity states of the genuine and simulating DID groups. Individuals with PTSD were recruited from various mental healthcare settings, and their diagnosis was confirmed through the use of the Clinician‐Administered PTSD Scale (Blake et al., 1995; Hovens et al., 2005). The CTRL‐HC group was recruited through advertisements in newspapers. They were informed that autobiographical memory was studied, but exact features of the other groups were not disclosed.

Data of 46 participants were analyzed in the present study: 13 participants with genuine DID (DID‐G), 11 actors to simulate DID (DID‐S), 11 healthy controls (CTRL‐HC), and 11 individuals with PTSD (CTRL‐PTSD). All participants were female (only female individuals with DID volunteered), of Western European ancestry, native speakers of Dutch, and aged between 18 and 65 years. Notably, a word rating pilot study conducted in an independent healthy control group showed that emotional and self‐relevance processing is gender independent (Dimitrova et al., 2022).

Data were gathered in Amsterdam and Groningen, the Netherlands. Ethical approval was acquired by the Amsterdam Medical Centre (reference number: MEC09/155) and the Medical Ethical Committee of the University Medical Centre Groningen (reference number: METC2008.211). Before participating, all individuals provided written consent after being informed in detail about the procedures, their right to withdraw at any time, and the anonymity and confidentiality of their personal data.

### 2.2 Procedure

**FIGURE 1:**
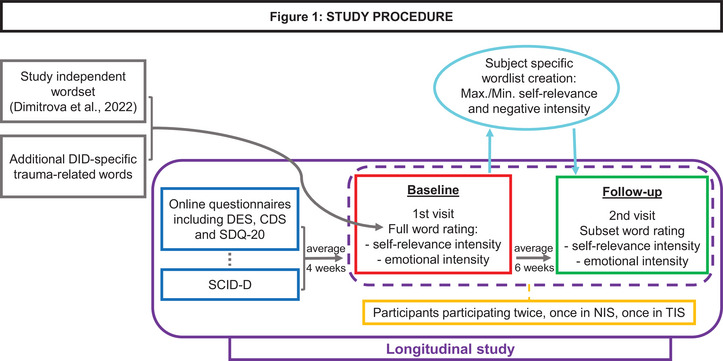
Study procedure. Words rated with maximum self‐relevance intensity and emotional intensity in the Baseline session were included as self‐relevant trauma‐related words (St) in the Follow‐up session. Conversely, words rated with minimum self‐relevance and maximum emotional intensity were incorporated as non‐self‐relevant trauma‐related word type (NSt). Finally, words rated with minimum self‐relevance and emotional intensity were selected as the non‐self‐relevant neutral word type (NSn). DES, Dissociative Experiences Scale; CDS, Cambridge Depersonalization Scale; SDQ‐20, Somatoform Dissociation Questionnaire; SCID‐D, Structured Clinical Interview for DSM‐IV Dissociative Disorders; Max., Maximum; Min., Minimum.

The overall procedure of the data acquisition is depicted in Figure 1. Data were acquired in two sessions with the software program Presentation version 14 (Neurobehavioural Systems, 2010) (coding by S.C.). During the first session, also referred to as “Baseline” session, the participants were asked to rate a list of 278 Dutch words, in terms of self‐relevance intensity and emotional intensity. These self‐relevance and intensity ratings are representative of the subjective autobiographical connotation and knowledge of the word under evaluation. The independent word list was obtained from a word evaluation study in the general population (Dimitrova et al., 2022) and included additional DID‐specific trauma‐related words. During this Baseline session, study participants assigned negative intensity values ranging from 0 (not‐negative) to 4 (very negative) and self‐relevance intensity scores ranging from 0 (not‐self‐relevant) to 2 (very self‐relevant) to each of these 278 words. A second word rating session was completed during the second visit, also referred to as “Follow‐up” session, which took place on average 6 weeks after the Baseline session (mean = 6.36, standard deviation = 5.93). In the Follow‐up session, study participants only rated subsets of subject‐specific words previously selected as most (S) or least (NS) self‐relevant and most (t) or least (n) emotionally intense at Baseline.

### 2.3 Materials

#### 2.3.1 Word lists

The full list of words presented during the Baseline Session is provided in Appendix D in the Supporting Information. Based on the Baseline session ratings, three subject‐specific individualized word lists were created for each participant to re‐rate in the Follow‐up session. The lists consisted of 20 non‐self‐relevant neutral (NSn: both intensity ratings were “0” at Baseline), 20 non‐self‐relevant traumatic (NSt: 0 for self‐relevance rating and the highest negativity intensity scores), and 20 self‐relevant traumatic (St: highest scores for both self‐relevance and negativity) words. Self‐relevant neutral words were not included, as Dimitrova et al. (2022) showed that individuals rate neutral words mostly as non‐self‐relevant.

#### 2.3.2 Dissociation scales

(1) The Cambridge Depersonalization Scale (CDS) (Sierra & Berrios, 2000) assessed the recurrence and duration of depersonalization and derealization symptoms; (2) the Dissociative Experiences Scale (DES) (Bernstein & Putnam, 1986) evaluated the frequency of cognitive–emotional dissociative phenomena; and (3) the Somatoform Dissociation Questionnaire (SDQ‐20) (Nijenhuis et al., 1996) measured the severity of somatoform (i.e., sensory and motor) dissociative phenomena, as detailed in Vissia et al. (2016). For the internal consistency and reliability scores of these scales, please refer to Appendix C in the Supporting Information.

### 2.4 Statistical analyses

Presentation log files from Baseline and Follow‐up sessions were processed using R (version 3.4.1; R Core Team, 2017) to calculate each participant's average negative intensity ratings, and average self‐relevance intensity ratings for each class of cues (St, NSt, NSn), at each session and dissociative identity state. IBM SPSS for Windows (Version 24) software was used for all statistical analyses. Demographics and trait dissociation levels were analyzed through nonparametric Kruskal–Wallis tests and post hoc Mann–Whitney tests, as the data did not meet the assumption of normal distribution or heterogeneity of variance. As detailed below, three‐ and four‐way mixed‐model analyses of variance (ANOVAs) were used for self‐relevance and emotional intensity ratings. In line with our null hypothesis, our analyses started with the highest order statistically significant interaction term featuring Group. Directional effects were probed with post hoc ANOVA tests using multiple comparisons (Fisher's LSD) and *t*‐tests to allow interpretation.

#### 2.4.1 Experimental manipulations and null hypothesis testing

We investigated whether our experimental manipulations of all three Word types were effective, that is, self‐relevant words being indeed rated as more self‐relevant than non‐self‐relevant ones, and trauma‐related words as more emotionally negative than neutral words. Additionally, we explored whether we could reject our null hypothesis that there would be no differences in ratings between groups at the two time points. A four‐way design was employed with three participant Groups (genuine DID [DID‐G], participants who simulated DID [DID‐S], and control group [(CTRL]) as the single between‐subjects factor and three within‐subject factors: two Sessions (Baseline and Follow‐up), two dissociative identity States (NIS and TIS), and three Word types (St, NSt, and NSn).

#### 2.4.2 Self‐relevance intensity ratings

Because we had only one Word type that was related to self‐relevance intensity ratings, namely, the St condition, a three‐way ANOVA design was applied with one between‐subject factor (Group: DID‐G, DID‐S, and CTRL) and two within‐subject factors (Session [Baseline and Follow‐up] and State [NIS and TIS]).

#### 2.4.3 Emotional intensity ratings

Of the three experimental conditions, only two had emotional valence, namely, St and NSt. Therefore, a four‐way design was employed with one between‐subject factor (Group: DID‐G, DID‐S, and CTRL) and three within‐subject factors (Session [Baseline, Follow‐up], State [NIS, TIS], and Word type [St, NSt]).

## 3 RESULTS

### 3.1 Participant characteristics

There were no statistically significant differences in age and education between the groups. Highly significant group differences in trait dissociation scores were found in the CDS, DES, and SDQ‐20 scales when comparing the DID‐G (diagnosed DID) group to the control groups (see Table 1).

**TABLE 1:** Participant groups’ demographic characteristics and statistic descriptives.

	Mean (*SD*)				ANOVA/Kruskal–Wallis		Post hoc tests (Mann–Whitney)					
	DID‐G (*n* = 13)	DID‐S (*n* = 11)	CTRL‐PTSD (*n* = 11)	CTRL‐HC (*n* = 11)	Statistic	Sign. (two‐tailed)	DID‐G vs. DID‐S	DID‐G vs. PTSD	DID‐G vs. HC	DID‐S vs. PTSD	DID‐S vs. HC	PTSD vs. HC
Age	44.15 (9.83)	42.89 (13.54)	38.23 (12.76)	43.16 (11.68)	*F*(3, 45) = 0.57	n.s.						
Education	15.08 (0.64)	14.91 (1.81)	14.82 (0.87)	15.27 (0.47)	*F*(3, 45) = 0.39	n.s.						
CDS^a^	132.69 (35.98)	18.60 (15.79)	70.18 (35.08)	20.64 (13.68)	*H*(3) = 33.04^*^	*p* < .001	*U* = 0, *p* < .001	*U* = 11, *p* < .001	*U* = 0, *p* < .001	*U* = 9, *p* < .001	n.s.	*U* = 10, *p* < .001
DES^a^	56.13 (15.94)	4.97 (2.96)	21.19 (11.69)	5.98 (4.10)	*H*(3) = 35.65^*^	*p* < .001	*U* = 0, *p* < .001	*U* = 6, *p* < .001	*U* = 0, *p* < .001	*U* = 4, *p* < .001	n.s.	*U* = 8, *p* < .001
SDQ‐20^a^	56.46 (17.35)	21.55 (1.69)	34.64 (14.62)	22.09 (2.63)	*H*(3) = 34.22^*^	*p* < .001	*U* = 0, *p* < .001	*U* = 20.5, *p* = .003	*U* = 0, *p* < .001	*U* = 4, *p* < .001	n.s.	*U* = 8, *p* < .001
Session interval	8.56 (8.84)	5.16 (3.05)	4.31 (2.19)	6.07 (3.44)	*H*(3) = 3.18	n.s.						

Abbreviations: CDS, Cambridge Depersonalization Scale; CTRL‐HC, healthy controls; CTRL‐PTSD, individuals diagnosed with posttraumatic stress disorder; DES, Dissociative Experiences Scale; DID‐G, individuals with genuine dissociative identity disorder; DID‐S, individuals simulating DID; n.s., nonsignificant outcome; SD, standard deviation; SDQ‐20, Somatoform Dissociation Questionnaire.

^a^ Data acquired in the most prominent NIS (neutral identity state).

* *p* ≤ .001.

### 3.2 Effectiveness of experimental manipulations and null hypothesis testing

#### 3.2.1 Main effects

We found a statistically significant main effect of session, dissociative identity state, and word type in the participants’ self‐relevance and emotional intensity ratings (see Tables 2 and 3).

**TABLE 2:** Three‐way multivariate ANOVA summary table for self‐relevance intensity ratings of the St words.

	Source of variation		Type III sum of squares	*df*	Mean square	*F*	Sig.	Partial eta squared
Within‐subjects effects	Session	Sphericity assumed	11.470	1	11.470	156.934^**^	<.001	.831
	Group × Session	Sphericity assumed	0.079	2	0.040	0.542	.587	.033
	Error(Session)	Sphericity assumed	2.339	32	0.073			
	State	Sphericity assumed	14.481	1	14.481	73.290^**^	<.001	.696
	Group × State	Sphericity assumed	0.811	2	0.406	2.053	.145	.114
	Error(State)	Sphericity assumed	6.323	32	0.198			
	Session × State	Sphericity assumed	0.991	1	0.991	17.170^**^	<.001	.349
	Group × Session × State	Sphericity assumed	0.103	2	0.051	0.890	.421	.053
	Error(Session × State)	Sphericity assumed	1.847	32	0.058			
Between‐subjects effects	Group		2.238	2	1.119	3.861^*^	.031	.194
	Error		9.277	32	0.290			

Abbreviation: St, self‐relevant trauma‐related.

* *p* ≤ .05.

** *p* ≤ .001.

**TABLE 3:** Four‐way multivariate ANOVA summary table for emotional intensity ratings of the St and NSt words.

	Source of variation		Type III sum of squares	*df*	Mean square	*F*	Sig.	Partial eta squared
Within‐subjects effects	Session	Sphericity assumed	13.190	1	13.190	27.996^**^	<.001	.467
	Group × Session	Sphericity assumed	0.594	2	0.297	0.630	.539	.038
	Error(Session)	Sphericity assumed	15.076	32	0.471			
	State	Sphericity assumed	3.268	1	3.268	5.304^*^	.028	.142
	Group × State	Sphericity assumed	0.879	2	0.440	0.713	.498	.043
	Error(State)	Sphericity assumed	19.720	32	0.616			
	Word type	Sphericity assumed	3.312	1	3.312	6.610^*^	.015	.171
	Group × Word type	Sphericity assumed	8.185	2	4.093	8.170^**^	.001	.338
	Error(Word type)	Sphericity assumed	16.031	32	0.501			
	Session × Word type	Sphericity assumed	0.549	1.000	0.549	5.141^*^	.030	.138
	Group × Session × Word type	Sphericity assumed	0.561	2.000	0.280	2.625^***^	.088	.141
	Error(Session × Word type)	Sphericity assumed	3.418	32.000	0.107			
	Session × State	Sphericity assumed	3.467 × 10^–2^	1	3.467 × 10^–2^	0.127	.724	.004
	Group × Session × State	Sphericity assumed	0.212	2	0.106	0.389	.681	.024
	Error(Session × State)	Sphericity assumed	8.712	32	0.272			
	State × Word type	Sphericity assumed	23.776	1	23.776	51.903^**^	<.001	.619
	Group × State × Word type	Sphericity assumed	3.923	2	1.961	4.282^*^	.023	.211
	Error(State × Word type)	Sphericity assumed	14.659	32	0.458			
	Session × State × Word type	Sphericity assumed	1.104	1	1.104	6.325^*^	.017	.165
	Group × Session × State × Word type	Sphericity assumed	0.145	2	0.072	0.415	.664	.025
	Error(Session × State × Word type)	Sphericity assumed	5.586	32	0.175			
Between‐subjects effects	Group		5.635	2	2.817	1.986	.154	.110
	Error		45.387	32	1.418			

Abbreviations: NSt, non‐self‐relevant trauma‐related; St, self‐relevant trauma‐related.

* *p* ≤ .05

** *p* ≤ .001

*** .05 ≤ *p* ≤ .1 (trend).

#### 3.2.2 Self‐relevance intensity

Our experimental design proved to be effective as the words selected for the non‐self‐relevant (NSn [non‐self‐relevant neutral; mean = 0.046, standard error = 0.012], NSt [non‐self‐relevant trauma‐related; *M* = 0.141, *SE* = 0.029]) cues were rated as less self‐relevant than the self‐relevant (St [self‐relevant trauma‐related; *M* = 1.223, *SE* = 0.049]) words (NSn—St: mean difference = −1.177, *SE* = 0.044, 95% confidence interval [CI]: [−1.27, −1.09], *p* < .001; NSt—St: mean difference = −1.082, *SE* = 0.041, 95% CI: [−1.17, −1.00], *p* < .001). These significant differences in word‐type ratings were only found in the Baseline session between groups (Baseline: *F*(2.404, 38.468) = 3.659, *p* = .028, *η*_p_^2^ = .186; Follow‐up: *F*(3.333, 53.321) = 0.764, *p* = .532, *η*_p_^2^ = .046), thus not supporting the rejection of our null hypothesis.

#### 3.2.3 Emotional intensity

Consistent with our design, the words selected as neutral (NSn [*M* = 0.211, *SE* = 0.031]) were rated less negatively than traumatic words (NSt [*M* = 2.627, *SE* = 0.094], St [*M* = 2.868, *SE* = 0.084]) (NSt—NSn: mean difference = 2.416, *SE* = 0.096, 95% CI: [2.22, 2.61], *p* < .001; St—NSn: mean difference = 2.657, *SE* = 0.078, 95% CI: [2.50, 2.82], *p* < .001). Similar to self‐relevance intensity ratings, significant differences in Word‐type ratings were observed in the Baseline session between groups (Baseline: *F*(3.301, 52.818) = 5.527, *p* = .002, *η*_p_^2^ = .257; Follow‐up: *F*(3.239, 51.830) = 1.775, *p* = .160, *η*_p_^2^ = .100), again not supporting the rejection of our null hypothesis.

### 3.3 Significant Group differences

#### 3.3.1 Self‐relevance intensity ratings

**FIGURE 2:**
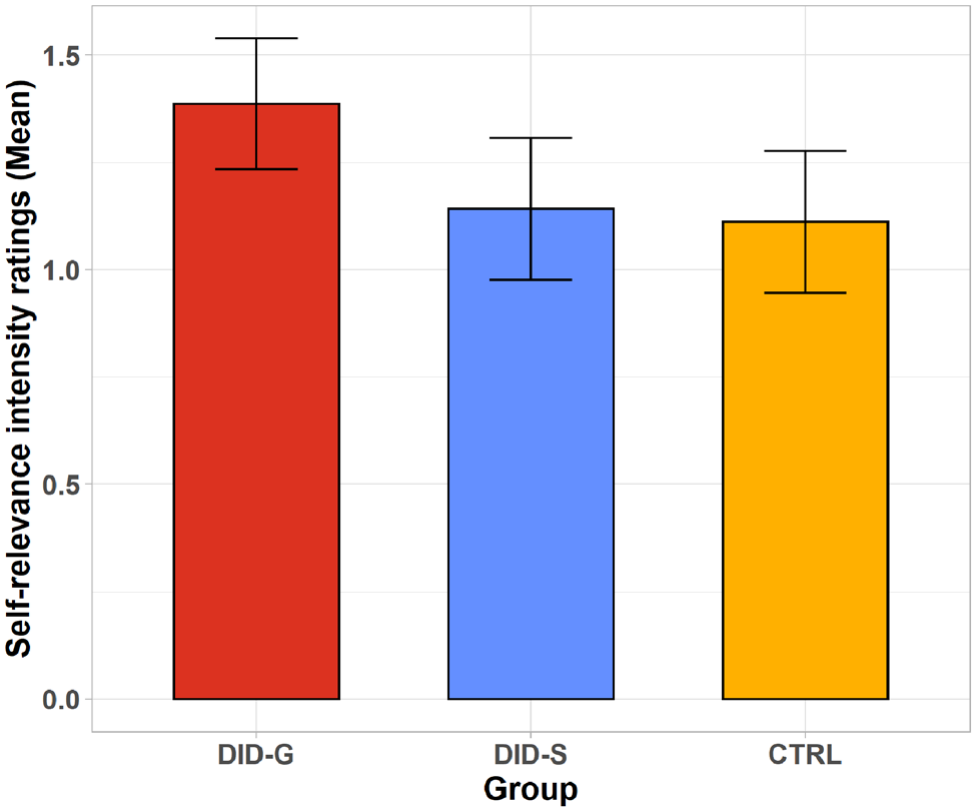
Self‐relevance intensity ratings of the St words in NIS and TIS. St, self‐relevant trauma‐related words; TIS, trauma‐related identity state; DID‐G, individuals with a diagnosis of dissociative identity disorder (DID), that is, genuine DID; DID‐S, DID‐simulating controls; CTRL, a paired control group of healthy participants (controls for the NIS) and individuals with a diagnosis of PTSD (controls for the TIS).

Neither the omnibus three‐way Group × Session × State interaction (*F*(2, 32) = 0.890, *p*‐value = .421, *η*_p_^2^ = .053) nor the two‐way interactions involving Group (*F*‐range: 0.5–2.1, *p*‐values all >.1) were significant (see Table 2). Only the main effect of Group reached statistical significance (*F*(2, 32) = 3.861, *p* = .031, *η*_p_^2^ = .194). Post hoc pairwise comparisons revealed significant differences between the DID‐G and CTRL (paired control group of individuals with PTSD and healthy controls) participants (mean difference = +0.276, *SE* = 0.110, 95% CI: [0.051, 0.500], *p* = .018), as well as between the DID‐G and DID‐S (DID‐simulating controls) groups (mean difference = +0.245, *SE* = 0.110, 95% CI: [0.021, 0.470], *p* = .033) (Figure 2).

#### 3.3.2 Emotional intensity ratings

**FIGURE 3:**
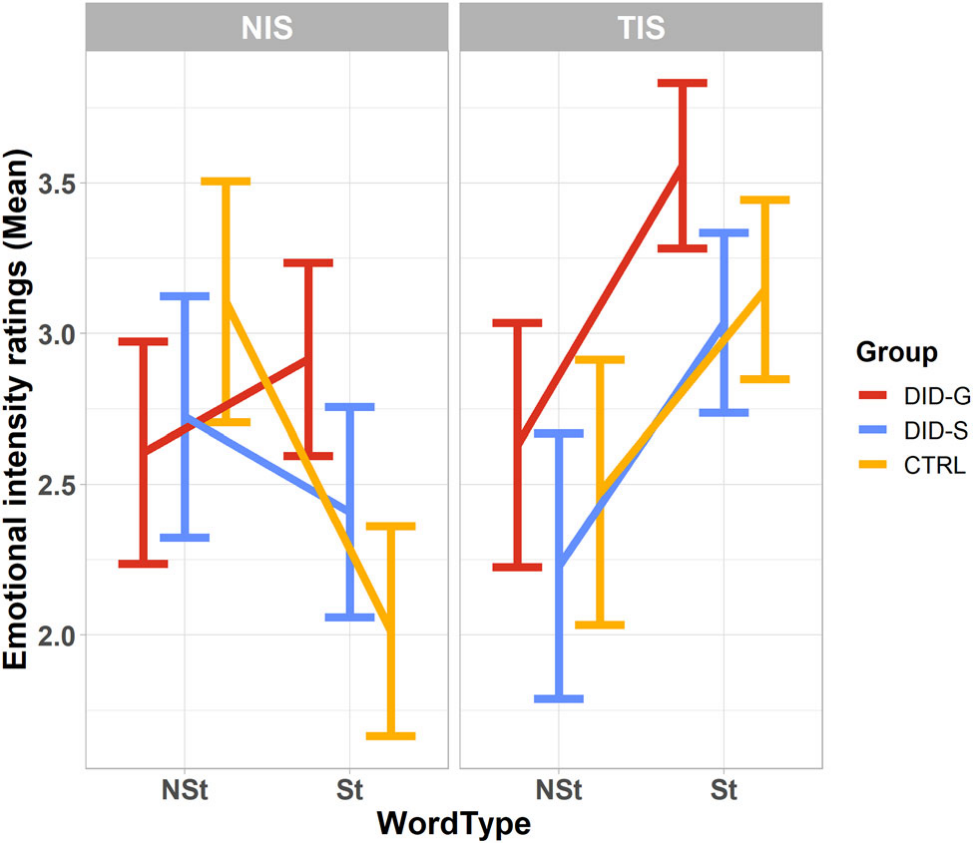
Emotional intensity ratings of the St and NSt words in NIS and TIS. St, self‐relevant trauma‐related; NSt, non‐self‐relevant trauma‐related; DID‐G, individuals with a diagnosis of dissociative identity disorder (DID), that is, genuine DID; DID‐S, DID‐simulating controls; CTRL, a paired control group of healthy participants (controls for the NIS) and individuals with a diagnosis of PTSD (controls for the TIS); NIS, neutral identity state; TIS, trauma‐related identity state.

The omnibus four‐way Group × Session × State × Word type interaction was not significant (*F*(2, 32) = 0.415, *p* = .664, *η*_p_^2^ = .025). Therefore, we proceeded with exploring the three‐way interactions involved in the main ANOVA (see Table 3). Of the three‐way interactions involving Group, only the Group × State × Word type interaction was statistically significant (*F*(2, 32) = 1.961, *p* = .023, *η*_p_^2^ = .211) (see Figure 3). To explore this interaction, we broke it down and investigated the two‐way Group × Word type interaction in ANOVA conducted at each level of State. The analysis revealed a significant Group × Word type interaction effect, only in the NIS (*F*(2, 32) = 10.247, *p* < .001, *η*_p_^2^ = .390), whereas in the TIS state there was a common effect of Word type (*F*(1, 32) = 57.903, *p* < .001, *η*_p_^2^ = .644) equal for all groups but no interaction with Group (*F*(2, 32) = 0.501, *p* = .611, *η*_p_^2^ = .030), as well as no main effect of Group (*F*(2, 32) = 2.352, *p* = .111, *η*_p_^2^ = .128). This common effect of Word type in the TIS state was such that all participants from all groups in the TIS state rated St words as more negatively emotionally valent as compared to NSt (mean difference = +0.803, *SE* = ±0.105, 95% CI: [0.588, 1.018], *p* < .001) (Figure 3). In contrast, in the NIS state the main effect of Word type (over all groups) indicated the St words were rated less negatively than NSt (mean difference = −0.366, *SE* = ± 0.128, 95% CI: [−0.628, −0.105], *p* = .007). However, significant differences were only observed for the CTRL group, such that CTRL participants rated St less negative than NSt (−1.20 ± 0.380, *p* < .001) (Figure 3).

## 4 DISCUSSION

The present study investigated the self‐relevancy and emotional intensity rating of words over time for individuals with genuine DID, DID‐simulating controls, and a paired group of healthy controls to represent the NIS and individuals with PTSD to represent the TIS (trauma‐related identity state). Our primary finding was that the three groups did not differ in how they rated subject‐specific words longitudinally. Our most important finding with regard to self‐relevance is that individuals with DID significantly rated the self‐relevant word type as more self‐relevant compared to the paired control group of healthy participants and individuals with PTSD. Another finding was that the ratings of emotionally valenced words were largely dependent on the dissociative identity state and most negatively rated in the TIS state across all participant groups.

All participant groups rated the self‐relevant (St) stimuli as more self‐relevant than the non‐self‐relevant (NSn, NSt), and the trauma‐related (NSt, St) words as more negative than the neutral (NSn) items, especially in the TIS. Importantly, these findings were observed across sessions and no significant session differences were found between the participant groups. This outcome concurs with the findings of a study by Coluccia et al. (2006), in which the consistency of students’ autobiographical knowledge following a traumatic experience was found to not be affected by time and might indicate that self‐relevance and emotional processing in individuals with DID changes over time in a similar way as in nontraumatized populations. Longitudinal research in PTSD and other psychiatric disorders (Dickie et al., 2011; Frías et al., 2018; Zhou et al., 2017) has also shown consistent processing over time, as well as considerable change in overall functioning following therapeutic treatment. Our results also expanded the outcomes of a previous study by Dorahy and colleagues (2021) that indicated that there were no significant differences in the sense of self over time between participants with diagnosed DID and populations with psychosis or healthy participants.

Our study extrapolates previous findings that DID does not result from suggestion and motivated role‐playing (Reinders & Veltman, 2021). Simulated and genuine DID rated the self‐relevancy and emotional intensity of subject‐specific words significantly different, which is also found in other studies documenting that individuals with DID score trauma‐related cues higher than various control groups, including DID simulators (Elzinga et al., 2003; Huntjens et al., 2016; Vissia et al., 2016). However, other studies found that DID simulators and individuals with PTSD tend to rate trauma‐related cues similarly to the genuine DID individuals (Boysen & VanBergen, 2014; Huntjens et al., 2016). An explanation for this discrepancy is that not all studies used subject‐specific trauma‐related information and therefore lack the ability to differentiate between the groups. With regard to psychophysiological and neural activation patterns, marked distinctions between participants with genuine DID and individuals who simulated DID were found (Reinders & Veltman, 2021; Vissia et al., 2022). Together, these studies suggest that DID does not involve simulation and provides further evidence that DID is a genuine and trauma‐related disorder.

Dissociative identity state‐dependent ratings were observed, such that participants recalled more negative and trauma‐related knowledge when functioning as a trauma‐related dissociative identity state compared to when functioning as a neutral dissociative identity state. These findings are in line with a previous study by Huntjens and colleagues (2016), which also included individuals with DID and DID simulators. They are also in line with brain imaging studies documenting that different prototypical dissociative identity states are associated with their own patterns of neural and physiological reactivity to trauma‐related cues and other cues that have a different meaning for these dissociative identity states (Reinders et al., 2003, 2006, 2014, 2012, 2016; Schlumpf et al., 2013, 2014, 2019).

### 4.1 Implications of the outcomes

The findings of the present study help to further understand the complex nature of DID, by demonstrating the existence of distinct differences in self‐relevance and emotional intensity processing between the NIS and the TIS of individuals with DID. Moreover, the marked differences between the groups in the NIS’ ratings highlight observations of clinicians that DID's NIS is not affectively neutral (Nijenhuis & Boer, 2011; van der Hart et al., 2010, 2004). Furthermore, consistent with observations of DID clinicians, the intensity ratings of individuals with DID in a TIS as compared to those of participants with PTSD were significantly different.

Finally, the outcomes of this study enrich our current knowledge regarding cognitive aspects of the disorder and reaffirm long‐standing clinical observations in a controlled research setting. Importantly, the evaluation of self‐relevant words over a period of weeks simulates the period between treatment sessions. This can inform and guide the advancement of treatment techniques for disorders involving trauma‐related dissociation.

### 4.2 Strengths and limitations

The important strength of the present study is the use of individualized word lists, as opposed to the majority of previously published research (for review, see Reinders et al., 2022). The consistency of word evaluation was assessed across a relatively short period of time as compared to other longitudinal studies, which can be considered a limitation of this study. However, within the realm of treatment where sessions are relatively close together, this is a more informative time span. During this 6‐week period between sessions, the participants continued with their life as normal. A positive aspect of our design is that the likelihood of confounding events is low and indeed none were reported to the researchers. A limitation of our study, as well as for many other longitudinal studies, is that we did not structurally assess confounding variables that could have impacted our outcomes of the study, and we recommend such assessments for follow‐up studies. The fact that only women volunteered could be considered a limitation. However, some comments have been made about the advantages of including same‐sex individuals, especially in imaging or cognition‐related studies (Bell et al., 2006; Goldstein et al., 2005). The sample used in the analyses of this study could be considered small, especially in comparison to the available literature of other psychiatric disorders. However, with regard to samples of individuals with a DID diagnosis, our sample is of average size and despite the relatively small numbers of participants, significant findings were found. Finally, we note that the equivalence of the session effect between groups was not formally tested with an equivalence testing procedure such as the Two One‐Sided Tests approach (Lakens, 2017) that would require a larger sample (Flight & Julious, 2016).

## 5 CONCLUSIONS

Self‐relevant and emotional processing significantly differs between individuals with DID, control groups, and DID‐simulating actors, in a consistent manner over time. Ratings of self‐relevant trauma‐related words are dissociative identity state dependent. These outcomes are in line with clinical observations.

### PEER REVIEW

The peer review history for this article is available at https://publons.com/publon/10.1002/brb3.3208.

## Supporting information


**Appendix A**: Table of key abbreviations, in alphabetical order.
**Appendix B**: Identity state criteria for participants with diagnosed DID.
**Appendix C**: Internal consistency and reliability scores of administered scales.
**Appendix D**: Table of all words selected by participants during the baseline session, sorted first by participant Group, and then by highest rated word.

## Data Availability

The data used to support the findings of this study are available from the corresponding author upon request.
