# Supplementary material for: Identity state‐dependent self‐relevance and emotional intensity ratings of words in dissociative identity disorder: A controlled longitudinal study
Source: Brain Behav. 2023 Sep 18;13(10):e3208. doi: 10.1002/brb3.3208 (PMC10570477; doi:10.1002/brb3.3208)
Supplement: Supplementary file 1 — Appendix A: Table of key abbreviations, in alphabetical order. Appendix B: Identity state criteria for participants with diagnosed DID. Appendix C: Internal consistency and reliability scores of administered scales. Appendix D: Table of all words selected by participants during the baseline session, sorted first by participant Group, and then by highest rated word. [file BRB3-13-e3208-s001.docx]

**SUPPLEMENTARY MATERIALS**

For paper entitled “Identity State-Dependent Self-Relevance and Emotional Intensity Ratings of Words in Dissociative Identity Disorder: A Controlled Longitudinal Study”

In submission for: Brain and Behavior

**Appendix A**: Table of key abbreviations, in alphabetical order.

| **Abbreviation** | **Definition** |
| --- | --- |
| ANOVA | Analysis of variance |
| CTRL | Paired control group |
| CTRL-HC | Healthy controls included in the paired group |
| CTRL-PTSD | Individuals with PTSD included in the paired group |
| DID | Dissociative identity disorder |
| DID-G | Individuals diagnosed with (genuine) DID |
| DID-S | DID simulating controls |
| NIS | Neutral identity state |
| NSn | Non-self-relevant neutral |
| NSt | Non-self-relevant trauma-related |
| PTSD | Post-traumatic stress disorder |
| St | Self-relevant trauma-related |
| TIS | Trauma-related identity state |

**Appendix B**: Identity state criteria for participants with diagnosed DID.

As Vissia et al. (2016) previously described in detail, the participants with genuine DID chose which NIS and TIS would participate in this study, in consultation with their therapists. Detailed descriptions of the selected identity states were provided by the participants for the researchers to review. The research team then confirmed that the selected NIS was capable to mentally avoid trauma-related memories, whereas the selected TIS had access to these memories (Vissia et al., 2016). During the research sessions, the researchers also ensured that the participants had voluntarily alternated between the two chosen states and the intended identity state under investigation was present. The specific characteristics of the diagnosed and simulated DID states have previously been described in detail in the Supplementary Materials of Vissia et al. (2016), which can be found online (https://onlinelibrary.wiley.com/action/downloadSupplement?doi=10.1111%2Facps.12590&file=acps12590-sup-0001-SupInfo.pdf).

**Appendix C**: Internal consistency and reliability scores of administered scales.

As previously detailed in Vissia et al. (2016), Sierra & Berrios (2000) have shown a high internal consistency (Cronbach's alpha=0.89) and good reliability (split-half reliability=0.92) on the Cambridge Depersonalization Scale (CDS). The Dissociative Experiences Scale (DES) has been shown to have high internal consistency, and the test-retest reliability ranges from 0.79 to 0.96 (Bernstein & Putnam, 1986; Frischholz et al., 1990). According to Vissia et al. (2016), various studies have indicated a high internal consistency of the Somatoform Dissociation Questionnaire (SDQ-20) (Nijenhuis et al., 1996), as well as convergent validity of this questionnaire with other measures of dissociation, including the DES (Nijenhuis et al., 1998; Sar et al., 2000).

**Appendix D**: Table of all words selected by participants during the baseline session, sorted first by participant Group, and then by highest rated word.

| **Group** | **State** | **TrialType** | **Text** | **eText** | **n** | **prop** |
| --- | --- | --- | --- | --- | --- | --- |
| DID-G^^n1^ | NIS | St | trauma | trauma | 11 | 79% |
| DID-G | NIS | NSt | roofmoord | robbery with murder | 11 | 79% |
| DID-G | NIS | NSt | wurgen | to strangle | 11 | 79% |
| DID-G | NIS | NSn | consulaat | consulate | 11 | 79% |
| DID-G | TIS | St | dwang | coercion or force | 10 | 71% |
| DID-G | NIS | NSt | atoombom | nuclear bomb | 10 | 71% |
| DID-G | NIS | NSt | martelen | to torture | 10 | 71% |
| DID-G | TIS | St | bang | afraid | 9 | 64% |
| DID-G | TIS | St | geweld | violence | 9 | 64% |
| DID-G | NIS | St | onmacht | powerlessness | 9 | 64% |
| DID-G | TIS | St | opsluiten | to lock up | 9 | 64% |
| DID-G | TIS | St | verkrachting | rape | 9 | 64% |
| DID-G | NIS | NSt | doodslag | manslaughter | 9 | 64% |
| DID-G | NIS | NSt | moord | murder | 9 | 64% |
| DID-G | TIS | NSn | ijzer | iron | 9 | 64% |
| DID-G | NIS | NSn | metselaar | bricklayer | 9 | 64% |
| DID-G | TIS | NSn | telegram | telegram | 9 | 64% |
| DID-G | TIS | NSn | zetel | seat | 9 | 64% |
| DID-G | TIS | St | angst | fear | 8 | 57% |
| DID-G | NIS | St | bang | afraid | 8 | 57% |
| DID-G | TIS | St | incest | incest | 8 | 57% |
| DID-G | NIS | St | onzeker | uncertain | 8 | 57% |
| DID-G | TIS | St | pijn | pain | 8 | 57% |
| DID-G | TIS | St | seks | sex | 8 | 57% |
| DID-G | NIS | St | verkrachting | rape | 8 | 57% |
| DID-G | TIS | NSt | atoombom | nuclear bomb | 8 | 57% |
| DID-G | NIS | NSt | bloedbad | bloodbath | 8 | 57% |
| DID-G | NIS | NSt | doden | to kill | 8 | 57% |
| DID-G | NIS | NSt | misdrijf | crime | 8 | 57% |
| DID-G | TIS | NSt | oorlog | war | 8 | 57% |
| DID-G | NIS | NSt | pedofiel | paedophile | 8 | 57% |
| DID-G | TIS | NSt | roofmoord | robbery with murder | 8 | 57% |
| DID-G | NIS | NSt | tiran | tyrant | 8 | 57% |
| DID-G | TIS | NSn | cirkel | circle | 8 | 57% |
| DID-G | TIS | NSn | magazijn | warehouse | 8 | 57% |
| DID-G | TIS | NSn | metaal | metal | 8 | 57% |
| DID-G | TIS | NSn | metselaar | bricklayer | 8 | 57% |
| DID-G | TIS | NSn | pilaar | pillar | 8 | 57% |
| DID-G | TIS | NSn | rad | wheel | 8 | 57% |
| DID-G | TIS | NSn | stellen | to set | 8 | 57% |
| DID-G | TIS | NSn | stomerij | drycleaner | 8 | 57% |
| DID-G | TIS | NSn | zegel | seal | 8 | 57% |
| DID-G | TIS | St | afsnauwen | to snap at | 7 | 50% |
| DID-G | NIS | St | afwijzing | rejection | 7 | 50% |
| DID-G | NIS | St | angst | fear | 7 | 50% |
| DID-G | TIS | St | domkop | idiot | 7 | 50% |
| DID-G | NIS | St | dwang | coercion or force | 7 | 50% |
| DID-G | NIS | St | dwingen | to coerce or to force | 7 | 50% |
| DID-G | NIS | St | geweld | violence | 7 | 50% |
| DID-G | TIS | St | haten | to hate | 7 | 50% |
| DID-G | TIS | St | kreunen | to moan | 7 | 50% |
| DID-G | TIS | St | onmacht | powerlessness | 7 | 50% |
| DID-G | NIS | St | schuldig | guilty | 7 | 50% |
| DID-G | TIS | St | schuldig | guilty | 7 | 50% |
| DID-G | TIS | St | stikken | to suffocate | 7 | 50% |
| DID-G | TIS | St | walging | disgust | 7 | 50% |
| DID-G | NIS | NSt | beroerte | stroke | 7 | 50% |
| DID-G | NIS | NSt | folteren | to torture | 7 | 50% |
| DID-G | NIS | NSt | oorlog | war | 7 | 50% |
| DID-G | NIS | NSt | orgie | orgy | 7 | 50% |
| DID-G | TIS | NSn | dakgoot | gutter | 7 | 50% |
| DID-G | NIS | NSn | deurknop | door knob | 7 | 50% |
| DID-G | NIS | NSn | firma | firm | 7 | 50% |
| DID-G | TIS | NSn | gebouw | building | 7 | 50% |
| DID-G | NIS | NSn | haak | hook | 7 | 50% |
| DID-G | NIS | NSn | kast | closet | 7 | 50% |
| DID-G | NIS | NSn | kozijn | window frame | 7 | 50% |
| DID-G | NIS | NSn | plank | shelf | 7 | 50% |
| DID-G | TIS | NSn | schroef | screw | 7 | 50% |
| DID-G | TIS | NSn | tegel | tile | 7 | 50% |
| DID-G | NIS | NSn | telegram | telegram | 7 | 50% |
| DID-G | NIS | NSn | trottoir | pavement | 7 | 50% |
| DID-G | NIS | NSn | versie | version | 7 | 50% |
| DID-G | NIS | NSn | vierkant | square | 7 | 50% |
| DID-G | NIS | St | alleen | alone | 6 | 43% |
| DID-G | TIS | St | alleen | alone | 6 | 43% |
| DID-G | NIS | St | conflict | conflict | 6 | 43% |
| DID-G | TIS | St | doorslikken | to swallow | 6 | 43% |
| DID-G | NIS | St | droefheid | sadness | 6 | 43% |
| DID-G | TIS | St | ellende | misery | 6 | 43% |
| DID-G | NIS | St | falen | to fail | 6 | 43% |
| DID-G | NIS | St | haten | to hate | 6 | 43% |
| DID-G | NIS | St | incest | incest | 6 | 43% |
| DID-G | NIS | St | ongewenst | unwanted | 6 | 43% |
| DID-G | NIS | St | ruzie | fight or quarrel | 6 | 43% |
| DID-G | NIS | St | seks | sex | 6 | 43% |
| DID-G | TIS | St | slet | slut | 6 | 43% |
| DID-G | NIS | St | stikken | to suffocate | 6 | 43% |
| DID-G | NIS | St | tegenslag | setback | 6 | 43% |
| DID-G | NIS | St | vader | father | 6 | 43% |
| DID-G | NIS | St | verdriet | sadness | 6 | 43% |
| DID-G | NIS | NSt | bordeel | brothel | 6 | 43% |
| DID-G | NIS | NSt | crimineel | criminal | 6 | 43% |
| DID-G | NIS | NSt | gezwel | tumor | 6 | 43% |
| DID-G | NIS | NSt | incest | incest | 6 | 43% |
| DID-G | NIS | NSt | misdaad | crime | 6 | 43% |
| DID-G | NIS | NSt | sadist | sadist | 6 | 43% |
| DID-G | NIS | NSt | treiteren | to harass | 6 | 43% |
| DID-G | TIS | NSn | consulaat | consulate | 6 | 43% |
| DID-G | TIS | NSn | dwerg | dwarf | 6 | 43% |
| DID-G | TIS | NSn | flacon | bottle or vial | 6 | 43% |
| DID-G | NIS | NSn | ijzer | iron | 6 | 43% |
| DID-G | NIS | NSn | ivoor | ivory | 6 | 43% |
| DID-G | NIS | NSn | kenteken | license plate | 6 | 43% |
| DID-G | NIS | NSn | kogel | bullet | 6 | 43% |
| DID-G | NIS | NSn | omroep | broadcasting | 6 | 43% |
| DID-G | TIS | NSn | omroep | broadcasting | 6 | 43% |
| DID-G | TIS | NSn | pasen | Easter | 6 | 43% |
| DID-G | NIS | NSn | regenton | rain barrel | 6 | 43% |
| DID-G | NIS | NSn | register | register | 6 | 43% |
| DID-G | NIS | NSn | roeren | to stir | 6 | 43% |
| DID-G | NIS | NSn | schroef | screw | 6 | 43% |
| DID-G | NIS | NSn | steil | steep | 6 | 43% |
| DID-G | TIS | NSn | tapijt | tapestry | 6 | 43% |
| DID-G | NIS | NSn | tegel | tile | 6 | 43% |
| DID-G | NIS | NSn | teller | counter | 6 | 43% |
| DID-G | TIS | NSn | theelepel | teaspoon | 6 | 43% |
| DID-G | TIS | NSn | trede | step | 6 | 43% |
| DID-G | TIS | NSn | trottoir | pavement | 6 | 43% |
| DID-G | TIS | NSn | vierkant | square | 6 | 43% |
| DID-G | NIS | NSn | zakje | little bag | 6 | 43% |
| DID-G | TIS | NSn | zandloper | hourglass | 6 | 43% |
| DID-G | TIS | St | afkeer | aversion | 5 | 36% |
| DID-G | TIS | St | bedreigen | to threaten | 5 | 36% |
| DID-G | TIS | St | bloedbad | bloodbath | 5 | 36% |
| DID-G | NIS | St | boos | angry | 5 | 36% |
| DID-G | TIS | St | buurman | neighbor | 5 | 36% |
| DID-G | TIS | St | doodgaan | to die | 5 | 36% |
| DID-G | NIS | St | ellende | misery | 5 | 36% |
| DID-G | TIS | St | folteren | to torture | 5 | 36% |
| DID-G | TIS | St | hijgen | to pant | 5 | 36% |
| DID-G | TIS | St | kreng | bitch | 5 | 36% |
| DID-G | TIS | St | lijden | to suffer | 5 | 36% |
| DID-G | TIS | St | man | man | 5 | 36% |
| DID-G | TIS | St | martelen | to torture | 5 | 36% |
| DID-G | NIS | St | misdrijf | crime | 5 | 36% |
| DID-G | NIS | St | moeder | mother | 5 | 36% |
| DID-G | NIS | St | pedofiel | paedophile | 5 | 36% |
| DID-G | TIS | St | slecht | bad | 5 | 36% |
| DID-G | TIS | St | stinken | to stink | 5 | 36% |
| DID-G | TIS | St | trappen | to kick | 5 | 36% |
| DID-G | TIS | St | trauma | trauma | 5 | 36% |
| DID-G | NIS | St | verlammen | to paralyze | 5 | 36% |
| DID-G | NIS | St | verraad | betrayal | 5 | 36% |
| DID-G | NIS | St | wanhoop | despair | 5 | 36% |
| DID-G | TIS | St | wreedheid | cruelty | 5 | 36% |
| DID-G | TIS | St | wurgen | to strangle | 5 | 36% |
| DID-G | TIS | St | zeer | ache | 5 | 36% |
| DID-G | TIS | NSt | armoede | poverty | 5 | 36% |
| DID-G | TIS | NSt | inbraak | burglary | 5 | 36% |
| DID-G | TIS | NSt | kanker | cancer | 5 | 36% |
| DID-G | NIS | NSt | lepra | leprosy | 5 | 36% |
| DID-G | TIS | NSt | lepra | leprosy | 5 | 36% |
| DID-G | NIS | NSt | messteek | knife stab | 5 | 36% |
| DID-G | NIS | NSt | miskraam | miscarriage | 5 | 36% |
| DID-G | NIS | NSt | mismaakt | deformed | 5 | 36% |
| DID-G | TIS | NSt | slet | slut | 5 | 36% |
| DID-G | NIS | NSt | steekwond | stab wound | 5 | 36% |
| DID-G | NIS | NSt | stikken | to suffocate | 5 | 36% |
| DID-G | NIS | NSt | verkrachting | rape | 5 | 36% |
| DID-G | NIS | NSt | vernielen | to vandalize | 5 | 36% |
| DID-G | NIS | NSt | verzuipen | to drown | 5 | 36% |
| DID-G | NIS | NSt | walging | disgust | 5 | 36% |
| DID-G | NIS | NSn | absorptie | absorption | 5 | 36% |
| DID-G | TIS | NSn | absorptie | absorption | 5 | 36% |
| DID-G | NIS | NSn | bestek | cutlery | 5 | 36% |
| DID-G | TIS | NSn | bladzijde | page | 5 | 36% |
| DID-G | TIS | NSn | citaat | quote | 5 | 36% |
| DID-G | NIS | NSn | dakgoot | gutter | 5 | 36% |
| DID-G | TIS | NSn | deurknop | door knob | 5 | 36% |
| DID-G | NIS | NSn | dozijn | dozen | 5 | 36% |
| DID-G | NIS | NSn | dwerg | dwarf | 5 | 36% |
| DID-G | TIS | NSn | element | element | 5 | 36% |
| DID-G | TIS | NSn | firma | firm | 5 | 36% |
| DID-G | NIS | NSn | flacon | bottle or vial | 5 | 36% |
| DID-G | NIS | NSn | gebouw | building | 5 | 36% |
| DID-G | NIS | NSn | grondstof | natural resource | 5 | 36% |
| DID-G | TIS | NSn | grondstof | natural resource | 5 | 36% |
| DID-G | NIS | NSn | hertogin | duchess | 5 | 36% |
| DID-G | TIS | NSn | ivoor | ivory | 5 | 36% |
| DID-G | TIS | NSn | juni | June | 5 | 36% |
| DID-G | TIS | NSn | kenteken | license plate | 5 | 36% |
| DID-G | TIS | NSn | laden | to load | 5 | 36% |
| DID-G | NIS | NSn | legpuzzel | jigsaw puzzle | 5 | 36% |
| DID-G | NIS | NSn | leuning | railing | 5 | 36% |
| DID-G | TIS | NSn | leuning | railing | 5 | 36% |
| DID-G | NIS | NSn | magazijn | warehouse | 5 | 36% |
| DID-G | NIS | NSn | metaal | metal | 5 | 36% |
| DID-G | TIS | NSn | plafond | ceiling | 5 | 36% |
| DID-G | TIS | NSn | plank | shelf | 5 | 36% |
| DID-G | NIS | NSn | regel | rule | 5 | 36% |
| DID-G | TIS | NSn | roeren | to stir | 5 | 36% |
| DID-G | NIS | NSn | stoelpoot | chair leg | 5 | 36% |
| DID-G | NIS | NSn | stomerij | drycleaner | 5 | 36% |
| DID-G | NIS | NSn | stukadoor | plasterer | 5 | 36% |
| DID-G | TIS | NSn | stukadoor | plasterer | 5 | 36% |
| DID-G | NIS | NSn | tapijt | tapestry | 5 | 36% |
| DID-G | TIS | NSn | teller | counter | 5 | 36% |
| DID-G | TIS | NSn | tijdperk | era | 5 | 36% |
| DID-G | NIS | NSn | traject | route | 5 | 36% |
| DID-G | NIS | NSn | trede | step | 5 | 36% |
| DID-G | NIS | NSn | treden | to step | 5 | 36% |
| DID-G | NIS | NSn | uitgever | publisher | 5 | 36% |
| DID-G | NIS | NSn | zegel | seal | 5 | 36% |
| DID-G | TIS | St | achterlaten | leave behind | 4 | 29% |
| DID-G | NIS | St | afkeer | aversion | 4 | 29% |
| DID-G | NIS | St | afkraken | to decry | 4 | 29% |
| DID-G | NIS | St | afscheid | goodbye | 4 | 29% |
| DID-G | TIS | St | afwijzing | rejection | 4 | 29% |
| DID-G | NIS | St | agressie | aggression | 4 | 29% |
| DID-G | TIS | St | bed | bed | 4 | 29% |
| DID-G | NIS | St | benauwen | to agitate | 4 | 29% |
| DID-G | TIS | St | boos | angry | 4 | 29% |
| DID-G | TIS | St | branden | to burn | 4 | 29% |
| DID-G | NIS | St | depressie | depression | 4 | 29% |
| DID-G | NIS | St | domkop | idiot | 4 | 29% |
| DID-G | NIS | St | doodgaan | to die | 4 | 29% |
| DID-G | TIS | St | doodslag | manslaughter | 4 | 29% |
| DID-G | TIS | St | doodsteek | deathblow | 4 | 29% |
| DID-G | TIS | St | dwingen | to coerce or to force | 4 | 29% |
| DID-G | NIS | St | kelder | basement | 4 | 29% |
| DID-G | TIS | St | kelder | basement | 4 | 29% |
| DID-G | NIS | St | lawaai | noise | 4 | 29% |
| DID-G | NIS | St | lijden | to suffer | 4 | 29% |
| DID-G | TIS | St | meester | master | 4 | 29% |
| DID-G | NIS | St | mislukt | failed | 4 | 29% |
| DID-G | NIS | St | nacht | night | 4 | 29% |
| DID-G | NIS | St | omkomen | to perish | 4 | 29% |
| DID-G | TIS | St | ongewenst | unwanted | 4 | 29% |
| DID-G | TIS | St | pedofiel | paedophile | 4 | 29% |
| DID-G | NIS | St | piekeren | to mull | 4 | 29% |
| DID-G | NIS | St | pijn | pain | 4 | 29% |
| DID-G | NIS | St | slecht | bad | 4 | 29% |
| DID-G | NIS | St | snauwen | to snarl | 4 | 29% |
| DID-G | TIS | St | vader | father | 4 | 29% |
| DID-G | TIS | St | verdriet | sadness | 4 | 29% |
| DID-G | TIS | St | verzuipen | to drown | 4 | 29% |
| DID-G | TIS | St | wanhoop | despair | 4 | 29% |
| DID-G | NIS | St | zeer | ache | 4 | 29% |
| DID-G | NIS | NSt | afblaffen | to bark at | 4 | 29% |
| DID-G | NIS | NSt | afgunst | envy | 4 | 29% |
| DID-G | TIS | NSt | afschuw | revulsion | 4 | 29% |
| DID-G | NIS | NSt | agressie | aggression | 4 | 29% |
| DID-G | NIS | NSt | bedreigen | to threaten | 4 | 29% |
| DID-G | TIS | NSt | bedrieger | deceiver | 4 | 29% |
| DID-G | TIS | NSt | bedrog | deceit | 4 | 29% |
| DID-G | TIS | NSt | bordeel | brothel | 4 | 29% |
| DID-G | NIS | NSt | chanteren | to blackmail | 4 | 29% |
| DID-G | TIS | NSt | crimineel | criminal | 4 | 29% |
| DID-G | NIS | NSt | diefstal | theft | 4 | 29% |
| DID-G | TIS | NSt | doodslag | manslaughter | 4 | 29% |
| DID-G | NIS | NSt | doodsteek | deathblow | 4 | 29% |
| DID-G | NIS | NSt | geweld | violence | 4 | 29% |
| DID-G | NIS | NSt | gijzeling | kidnapping | 4 | 29% |
| DID-G | TIS | NSt | gijzeling | kidnapping | 4 | 29% |
| DID-G | NIS | NSt | kanker | cancer | 4 | 29% |
| DID-G | NIS | NSt | kreng | bitch | 4 | 29% |
| DID-G | NIS | NSt | krenken | to hurt | 4 | 29% |
| DID-G | NIS | NSt | omkomen | to perish | 4 | 29% |
| DID-G | NIS | NSt | opsluiten | to lock up | 4 | 29% |
| DID-G | TIS | NSt | pedofiel | paedophile | 4 | 29% |
| DID-G | NIS | NSt | slet | slut | 4 | 29% |
| DID-G | TIS | NSt | slijmen | suck up to | 4 | 29% |
| DID-G | NIS | NSt | snauwen | to snarl | 4 | 29% |
| DID-G | NIS | NSt | verdrinken | to drown | 4 | 29% |
| DID-G | NIS | NSt | verraad | betrayal | 4 | 29% |
| DID-G | NIS | NSt | wreedheid | cruelty | 4 | 29% |
| DID-G | TIS | NSt | wurgen | to strangle | 4 | 29% |
| DID-G | NIS | NSn | aanmaken | to prepare | 4 | 29% |
| DID-G | TIS | NSn | aanmaken | to prepare | 4 | 29% |
| DID-G | TIS | NSn | bestek | cutlery | 4 | 29% |
| DID-G | TIS | NSn | blikje | can | 4 | 29% |
| DID-G | NIS | NSn | element | element | 4 | 29% |
| DID-G | NIS | NSn | elleboog | elbow | 4 | 29% |
| DID-G | TIS | NSn | elleboog | elbow | 4 | 29% |
| DID-G | TIS | NSn | etiket | label | 4 | 29% |
| DID-G | NIS | NSn | geit | goat | 4 | 29% |
| DID-G | TIS | NSn | hagedis | lizard | 4 | 29% |
| DID-G | NIS | NSn | juni | June | 4 | 29% |
| DID-G | TIS | NSn | kast | closet | 4 | 29% |
| DID-G | NIS | NSn | meester | master | 4 | 29% |
| DID-G | NIS | NSn | middel | middle | 4 | 29% |
| DID-G | NIS | NSn | mond | mouth | 4 | 29% |
| DID-G | TIS | NSn | octaaf | octave | 4 | 29% |
| DID-G | TIS | NSn | ontslag | resignation or dismissal | 4 | 29% |
| DID-G | NIS | NSn | paragraaf | paragraph | 4 | 29% |
| DID-G | NIS | NSn | pilaar | pillar | 4 | 29% |
| DID-G | NIS | NSn | plafond | ceiling | 4 | 29% |
| DID-G | NIS | NSn | postzegel | stamp | 4 | 29% |
| DID-G | TIS | NSn | potlood | pencil | 4 | 29% |
| DID-G | NIS | NSn | rad | wheel | 4 | 29% |
| DID-G | TIS | NSn | regenton | rain barrel | 4 | 29% |
| DID-G | NIS | NSn | schuren | to polish | 4 | 29% |
| DID-G | NIS | NSn | stellen | to set | 4 | 29% |
| DID-G | NIS | NSn | stoel | chair | 4 | 29% |
| DID-G | TIS | NSn | stoelpoot | chair leg | 4 | 29% |
| DID-G | TIS | NSn | takken | branches | 4 | 29% |
| DID-G | NIS | NSn | theelepel | teaspoon | 4 | 29% |
| DID-G | NIS | NSn | tijdperk | era | 4 | 29% |
| DID-G | TIS | NSn | traject | route | 4 | 29% |
| DID-G | TIS | NSn | treden | to step | 4 | 29% |
| DID-G | TIS | NSn | uitgever | publisher | 4 | 29% |
| DID-G | NIS | NSn | vergroten | enlarge | 4 | 29% |
| DID-G | NIS | NSn | zandloper | hourglass | 4 | 29% |
| DID-G | TIS | St | afgrijzen | horror | 3 | 21% |
| DID-G | NIS | St | afschuw | revulsion | 3 | 21% |
| DID-G | NIS | St | afsnauwen | to snap at | 3 | 21% |
| DID-G | TIS | St | agressie | aggression | 3 | 21% |
| DID-G | NIS | St | armoede | poverty | 3 | 21% |
| DID-G | NIS | St | belazerd | fooled | 3 | 21% |
| DID-G | TIS | St | belazerd | fooled | 3 | 21% |
| DID-G | NIS | St | bloedbad | bloodbath | 3 | 21% |
| DID-G | NIS | St | doodslag | manslaughter | 3 | 21% |
| DID-G | NIS | St | doodsteek | deathblow | 3 | 21% |
| DID-G | NIS | St | doorslikken | to swallow | 3 | 21% |
| DID-G | TIS | St | droefheid | sadness | 3 | 21% |
| DID-G | NIS | St | ergeren | to annoy | 3 | 21% |
| DID-G | NIS | St | ergernis | annoyance | 3 | 21% |
| DID-G | NIS | St | etiket | label | 3 | 21% |
| DID-G | TIS | St | handen | hands | 3 | 21% |
| DID-G | NIS | St | hijgen | to pant | 3 | 21% |
| DID-G | TIS | St | hysterie | hysteria | 3 | 21% |
| DID-G | NIS | St | instorten | to collapse | 3 | 21% |
| DID-G | NIS | St | kotsen | to vomit | 3 | 21% |
| DID-G | NIS | St | kou | cold | 3 | 21% |
| DID-G | TIS | St | kuthoer | pussy whore | 3 | 21% |
| DID-G | NIS | St | liegen | to lie | 3 | 21% |
| DID-G | NIS | St | man | man | 3 | 21% |
| DID-G | NIS | St | martelen | to torture | 3 | 21% |
| DID-G | NIS | St | miskraam | miscarriage | 3 | 21% |
| DID-G | TIS | St | moeder | mother | 3 | 21% |
| DID-G | NIS | St | moord | murder | 3 | 21% |
| DID-G | NIS | St | ontrouw | unfaithful | 3 | 21% |
| DID-G | TIS | St | oom | uncle | 3 | 21% |
| DID-G | NIS | St | opsluiten | to lock up | 3 | 21% |
| DID-G | NIS | St | piemel | willy | 3 | 21% |
| DID-G | TIS | St | ruzie | fight or quarrel | 3 | 21% |
| DID-G | TIS | St | sadist | sadist | 3 | 21% |
| DID-G | NIS | St | schok | shock | 3 | 21% |
| DID-G | TIS | St | schoppen | to kick | 3 | 21% |
| DID-G | TIS | St | snijden | to cut | 3 | 21% |
| DID-G | TIS | St | stank | stench | 3 | 21% |
| DID-G | TIS | St | steekwond | stab wound | 3 | 21% |
| DID-G | TIS | St | treiteren | to harass | 3 | 21% |
| DID-G | NIS | St | uitgescholden | scolded | 3 | 21% |
| DID-G | TIS | St | uitlachen | to laugh at | 3 | 21% |
| DID-G | TIS | St | vastbinden | to tie | 3 | 21% |
| DID-G | TIS | St | verdrinken | to drown | 3 | 21% |
| DID-G | TIS | St | vies | dirty | 3 | 21% |
| DID-G | NIS | St | wanhopen | to despair | 3 | 21% |
| DID-G | TIS | St | woest | enraged | 3 | 21% |
| DID-G | NIS | St | zeuren | to nag | 3 | 21% |
| DID-G | TIS | St | zondebok | scapegoat | 3 | 21% |
| DID-G | NIS | NSt | aanslag | attack | 3 | 21% |
| DID-G | TIS | NSt | aanslag | attack | 3 | 21% |
| DID-G | TIS | NSt | afwijzing | rejection | 3 | 21% |
| DID-G | NIS | NSt | angst | fear | 3 | 21% |
| DID-G | NIS | NSt | bedrieger | deceiver | 3 | 21% |
| DID-G | NIS | NSt | bedrog | deceit | 3 | 21% |
| DID-G | TIS | NSt | beroerte | stroke | 3 | 21% |
| DID-G | TIS | NSt | besmetten | to contaminate | 3 | 21% |
| DID-G | NIS | NSt | blinddoek | blindfold | 3 | 21% |
| DID-G | TIS | NSt | bloedbad | bloodbath | 3 | 21% |
| DID-G | NIS | NSt | boos | angry | 3 | 21% |
| DID-G | TIS | NSt | diefstal | theft | 3 | 21% |
| DID-G | NIS | NSt | etter | pus | 3 | 21% |
| DID-G | NIS | NSt | getreiter | harassment | 3 | 21% |
| DID-G | NIS | NSt | hoer | whore | 3 | 21% |
| DID-G | NIS | NSt | inbraak | burglary | 3 | 21% |
| DID-G | TIS | NSt | incest | incest | 3 | 21% |
| DID-G | NIS | NSt | instorten | to collapse | 3 | 21% |
| DID-G | NIS | NSt | kuthoer | pussy whore | 3 | 21% |
| DID-G | TIS | NSt | kuthoer | pussy whore | 3 | 21% |
| DID-G | NIS | NSt | kwetsen | to hurt | 3 | 21% |
| DID-G | TIS | NSt | messteek | knife stab | 3 | 21% |
| DID-G | NIS | NSt | noodkreet | cry for help | 3 | 21% |
| DID-G | NIS | NSt | onrecht | injustice | 3 | 21% |
| DID-G | NIS | NSt | ontrouw | unfaithful | 3 | 21% |
| DID-G | TIS | NSt | ontrouw | unfaithful | 3 | 21% |
| DID-G | NIS | NSt | oplichten | to scam | 3 | 21% |
| DID-G | TIS | NSt | oplichten | to scam | 3 | 21% |
| DID-G | TIS | NSt | orgie | orgy | 3 | 21% |
| DID-G | NIS | NSt | piemel | willy | 3 | 21% |
| DID-G | TIS | NSt | ruzie | fight or quarrel | 3 | 21% |
| DID-G | NIS | NSt | schande | shame | 3 | 21% |
| DID-G | NIS | NSt | slaaf | slave | 3 | 21% |
| DID-G | TIS | NSt | snijden | to cut | 3 | 21% |
| DID-G | TIS | NSt | steekwond | stab wound | 3 | 21% |
| DID-G | NIS | NSt | stinken | to stink | 3 | 21% |
| DID-G | TIS | NSt | verdord | withered | 3 | 21% |
| DID-G | NIS | NSt | wanhoop | despair | 3 | 21% |
| DID-G | TIS | NSt | wraak | revenge | 3 | 21% |
| DID-G | TIS | NSn | atoombom | nuclear bomb | 3 | 21% |
| DID-G | TIS | NSn | bad | bath | 3 | 21% |
| DID-G | NIS | NSn | begraven | to bury | 3 | 21% |
| DID-G | NIS | NSn | chauffeur | driver | 3 | 21% |
| DID-G | NIS | NSn | cirkel | circle | 3 | 21% |
| DID-G | NIS | NSn | citaat | quote | 3 | 21% |
| DID-G | NIS | NSn | document | document | 3 | 21% |
| DID-G | NIS | NSn | etiket | label | 3 | 21% |
| DID-G | NIS | NSn | hagedis | lizard | 3 | 21% |
| DID-G | TIS | NSn | hertogin | duchess | 3 | 21% |
| DID-G | TIS | NSn | kerk | church | 3 | 21% |
| DID-G | NIS | NSn | krabben | to scratch | 3 | 21% |
| DID-G | NIS | NSn | laden | to load | 3 | 21% |
| DID-G | TIS | NSn | lepra | leprosy | 3 | 21% |
| DID-G | NIS | NSn | ogen | eyes | 3 | 21% |
| DID-G | NIS | NSn | ontslag | resignation or dismissal | 3 | 21% |
| DID-G | TIS | NSn | paragraaf | paragraph | 3 | 21% |
| DID-G | NIS | NSn | pasen | Easter | 3 | 21% |
| DID-G | TIS | NSn | postzegel | stamp | 3 | 21% |
| DID-G | TIS | NSn | register | register | 3 | 21% |
| DID-G | TIS | NSn | schuren | to polish | 3 | 21% |
| DID-G | TIS | NSn | stoep | sidewalk | 3 | 21% |
| DID-G | NIS | NSn | touw | rope | 3 | 21% |
| DID-G | TIS | NSn | touw | rope | 3 | 21% |
| DID-G | NIS | NSn | trappen | to kick | 3 | 21% |
| DID-G | TIS | NSn | vergroten | enlarge | 3 | 21% |
| DID-G | NIS | NSn | vreemde | stranger | 3 | 21% |
| DID-G | TIS | NSn | zakje | little bag | 3 | 21% |
| DID-G | NIS | NSn | zetel | seat | 3 | 21% |
| DID-G | TIS | St | afblaffen | to bark at | 2 | 14% |
| DID-G | NIS | St | afgunst | envy | 2 | 14% |
| DID-G | TIS | St | afkraken | to decry | 2 | 14% |
| DID-G | TIS | St | armoede | poverty | 2 | 14% |
| DID-G | NIS | St | bed | bed | 2 | 14% |
| DID-G | NIS | St | bedreigen | to threaten | 2 | 14% |
| DID-G | NIS | St | bedrieger | deceiver | 2 | 14% |
| DID-G | NIS | St | begraven | to bury | 2 | 14% |
| DID-G | TIS | St | benauwen | to agitate | 2 | 14% |
| DID-G | TIS | St | besmetten | to contaminate | 2 | 14% |
| DID-G | NIS | St | bloot | naked | 2 | 14% |
| DID-G | TIS | St | bordeel | brothel | 2 | 14% |
| DID-G | NIS | St | branden | to burn | 2 | 14% |
| DID-G | NIS | St | buurman | neighbor | 2 | 14% |
| DID-G | NIS | St | chanteren | to blackmail | 2 | 14% |
| DID-G | TIS | St | chanteren | to blackmail | 2 | 14% |
| DID-G | TIS | St | conflict | conflict | 2 | 14% |
| DID-G | TIS | St | crimineel | criminal | 2 | 14% |
| DID-G | NIS | St | doden | to kill | 2 | 14% |
| DID-G | TIS | St | dokter | (general practice) doctor | 2 | 14% |
| DID-G | NIS | St | dreigen | to threaten | 2 | 14% |
| DID-G | TIS | St | droevig | sad | 2 | 14% |
| DID-G | NIS | St | dubbel | double | 2 | 14% |
| DID-G | NIS | St | etter | pus | 2 | 14% |
| DID-G | NIS | St | folteren | to torture | 2 | 14% |
| DID-G | TIS | St | gaatje | little hole | 2 | 14% |
| DID-G | NIS | St | handen | hands | 2 | 14% |
| DID-G | NIS | St | hoer | whore | 2 | 14% |
| DID-G | TIS | St | instorten | to collapse | 2 | 14% |
| DID-G | NIS | St | kader | framework | 2 | 14% |
| DID-G | NIS | St | kanker | cancer | 2 | 14% |
| DID-G | NIS | St | kast | closet | 2 | 14% |
| DID-G | TIS | St | kast | closet | 2 | 14% |
| DID-G | NIS | St | kerk | church | 2 | 14% |
| DID-G | TIS | St | kotsen | to vomit | 2 | 14% |
| DID-G | TIS | St | kou | cold | 2 | 14% |
| DID-G | NIS | St | kwetsen | to hurt | 2 | 14% |
| DID-G | TIS | St | kwetsen | to hurt | 2 | 14% |
| DID-G | TIS | St | lafaard | coward | 2 | 14% |
| DID-G | TIS | St | likken | to lick | 2 | 14% |
| DID-G | NIS | St | masker | mask | 2 | 14% |
| DID-G | TIS | St | masker | mask | 2 | 14% |
| DID-G | NIS | St | messteek | knife stab | 2 | 14% |
| DID-G | TIS | St | messteek | knife stab | 2 | 14% |
| DID-G | TIS | St | misdaad | crime | 2 | 14% |
| DID-G | TIS | St | misdrijf | crime | 2 | 14% |
| DID-G | NIS | St | moedeloos | despondent | 2 | 14% |
| DID-G | TIS | St | nacht | night | 2 | 14% |
| DID-G | TIS | St | ongeluk | accident | 2 | 14% |
| DID-G | NIS | St | onrecht | injustice | 2 | 14% |
| DID-G | TIS | St | onrecht | injustice | 2 | 14% |
| DID-G | TIS | St | onzeker | uncertain | 2 | 14% |
| DID-G | NIS | St | oom | uncle | 2 | 14% |
| DID-G | TIS | St | piemel | willy | 2 | 14% |
| DID-G | NIS | St | sadist | sadist | 2 | 14% |
| DID-G | NIS | St | schoft | bastard | 2 | 14% |
| DID-G | TIS | St | schuren | to polish | 2 | 14% |
| DID-G | TIS | St | slaaf | slave | 2 | 14% |
| DID-G | TIS | St | snauwen | to snarl | 2 | 14% |
| DID-G | NIS | St | snijden | to cut | 2 | 14% |
| DID-G | NIS | St | stank | stench | 2 | 14% |
| DID-G | NIS | St | steil | steep | 2 | 14% |
| DID-G | TIS | St | stiekem | secretly | 2 | 14% |
| DID-G | NIS | St | tiran | tyrant | 2 | 14% |
| DID-G | TIS | St | tiran | tyrant | 2 | 14% |
| DID-G | NIS | St | trappen | to kick | 2 | 14% |
| DID-G | NIS | St | treiteren | to harass | 2 | 14% |
| DID-G | NIS | St | vastbinden | to tie | 2 | 14% |
| DID-G | NIS | St | verbranden | to burn | 2 | 14% |
| DID-G | TIS | St | vergroten | enlarge | 2 | 14% |
| DID-G | TIS | St | verlammen | to paralyze | 2 | 14% |
| DID-G | TIS | St | verleidster | temptress | 2 | 14% |
| DID-G | TIS | St | verraad | betrayal | 2 | 14% |
| DID-G | NIS | St | verzuipen | to drown | 2 | 14% |
| DID-G | NIS | St | vies | dirty | 2 | 14% |
| DID-G | NIS | St | vuur | fire | 2 | 14% |
| DID-G | TIS | St | wanhopen | to despair | 2 | 14% |
| DID-G | NIS | St | woest | enraged | 2 | 14% |
| DID-G | NIS | St | wurgen | to strangle | 2 | 14% |
| DID-G | TIS | St | zuigen | to suck | 2 | 14% |
| DID-G | NIS | NSt | achterlaten | leave behind | 2 | 14% |
| DID-G | TIS | NSt | achterlaten | leave behind | 2 | 14% |
| DID-G | TIS | NSt | afgunst | envy | 2 | 14% |
| DID-G | TIS | NSt | afhakken | to chop off | 2 | 14% |
| DID-G | NIS | NSt | afkraken | to decry | 2 | 14% |
| DID-G | TIS | NSt | afscheid | goodbye | 2 | 14% |
| DID-G | NIS | NSt | afschuw | revulsion | 2 | 14% |
| DID-G | NIS | NSt | armoede | poverty | 2 | 14% |
| DID-G | NIS | NSt | besmetten | to contaminate | 2 | 14% |
| DID-G | TIS | NSt | conflict | conflict | 2 | 14% |
| DID-G | NIS | NSt | depressie | depression | 2 | 14% |
| DID-G | TIS | NSt | doodsteek | deathblow | 2 | 14% |
| DID-G | NIS | NSt | dreigen | to threaten | 2 | 14% |
| DID-G | NIS | NSt | dwang | coercion or force | 2 | 14% |
| DID-G | NIS | NSt | embryo | embryo | 2 | 14% |
| DID-G | TIS | NSt | etter | pus | 2 | 14% |
| DID-G | TIS | NSt | folteren | to torture | 2 | 14% |
| DID-G | TIS | NSt | gezwel | tumor | 2 | 14% |
| DID-G | NIS | NSt | haten | to hate | 2 | 14% |
| DID-G | NIS | NSt | hijgen | to pant | 2 | 14% |
| DID-G | TIS | NSt | hijgen | to pant | 2 | 14% |
| DID-G | TIS | NSt | hoer | whore | 2 | 14% |
| DID-G | NIS | NSt | hysterie | hysteria | 2 | 14% |
| DID-G | TIS | NSt | hysterie | hysteria | 2 | 14% |
| DID-G | NIS | NSt | kelder | basement | 2 | 14% |
| DID-G | NIS | NSt | knijpen | to pinch | 2 | 14% |
| DID-G | NIS | NSt | kotsen | to vomit | 2 | 14% |
| DID-G | TIS | NSt | kotsen | to vomit | 2 | 14% |
| DID-G | NIS | NSt | kreunen | to moan | 2 | 14% |
| DID-G | NIS | NSt | laden | to load | 2 | 14% |
| DID-G | TIS | NSt | lafaard | coward | 2 | 14% |
| DID-G | NIS | NSt | liegen | to lie | 2 | 14% |
| DID-G | TIS | NSt | liegen | to lie | 2 | 14% |
| DID-G | NIS | NSt | lijden | to suffer | 2 | 14% |
| DID-G | NIS | NSt | likken | to lick | 2 | 14% |
| DID-G | NIS | NSt | masker | mask | 2 | 14% |
| DID-G | NIS | NSt | meeloper | opportunist | 2 | 14% |
| DID-G | TIS | NSt | meeloper | opportunist | 2 | 14% |
| DID-G | TIS | NSt | misdaad | crime | 2 | 14% |
| DID-G | TIS | NSt | misdrijf | crime | 2 | 14% |
| DID-G | NIS | NSt | mislukt | failed | 2 | 14% |
| DID-G | NIS | NSt | ongeluk | accident | 2 | 14% |
| DID-G | TIS | NSt | ongeval | accident | 2 | 14% |
| DID-G | NIS | NSt | ontslag | resignation or dismissal | 2 | 14% |
| DID-G | TIS | NSt | oom | uncle | 2 | 14% |
| DID-G | NIS | NSt | razernij | fury | 2 | 14% |
| DID-G | TIS | NSt | schoft | bastard | 2 | 14% |
| DID-G | NIS | NSt | schuldig | guilty | 2 | 14% |
| DID-G | TIS | NSt | slaaf | slave | 2 | 14% |
| DID-G | NIS | NSt | snijden | to cut | 2 | 14% |
| DID-G | NIS | NSt | stank | stench | 2 | 14% |
| DID-G | NIS | NSt | stiekem | secretly | 2 | 14% |
| DID-G | TIS | NSt | stiekem | secretly | 2 | 14% |
| DID-G | TIS | NSt | stikken | to suffocate | 2 | 14% |
| DID-G | TIS | NSt | tegenslag | setback | 2 | 14% |
| DID-G | NIS | NSt | trappen | to kick | 2 | 14% |
| DID-G | NIS | NSt | uitjouwen | to boo | 2 | 14% |
| DID-G | TIS | NSt | uitjouwen | to boo | 2 | 14% |
| DID-G | NIS | NSt | uitkleden | to undress | 2 | 14% |
| DID-G | TIS | NSt | verbranden | to burn | 2 | 14% |
| DID-G | NIS | NSt | verlammen | to paralyze | 2 | 14% |
| DID-G | NIS | NSt | verleidster | temptress | 2 | 14% |
| DID-G | TIS | NSt | verleidster | temptress | 2 | 14% |
| DID-G | NIS | NSt | vies | dirty | 2 | 14% |
| DID-G | NIS | NSt | wraak | revenge | 2 | 14% |
| DID-G | TIS | NSn | aanslag | attack | 2 | 14% |
| DID-G | TIS | NSn | achterlaten | leave behind | 2 | 14% |
| DID-G | NIS | NSn | afhakken | to chop off | 2 | 14% |
| DID-G | NIS | NSn | bad | bath | 2 | 14% |
| DID-G | NIS | NSn | benauwen | to agitate | 2 | 14% |
| DID-G | NIS | NSn | bladzijde | page | 2 | 14% |
| DID-G | NIS | NSn | blikje | can | 2 | 14% |
| DID-G | NIS | NSn | bloot | naked | 2 | 14% |
| DID-G | TIS | NSn | broer | brother | 2 | 14% |
| DID-G | TIS | NSn | chauffeur | driver | 2 | 14% |
| DID-G | NIS | NSn | conflict | conflict | 2 | 14% |
| DID-G | TIS | NSn | depressie | depression | 2 | 14% |
| DID-G | TIS | NSn | doodsteek | deathblow | 2 | 14% |
| DID-G | TIS | NSn | dozijn | dozen | 2 | 14% |
| DID-G | TIS | NSn | dubbel | double | 2 | 14% |
| DID-G | NIS | NSn | embryo | embryo | 2 | 14% |
| DID-G | TIS | NSn | embryo | embryo | 2 | 14% |
| DID-G | NIS | NSn | ergeren | to annoy | 2 | 14% |
| DID-G | TIS | NSn | gaatje | little hole | 2 | 14% |
| DID-G | TIS | NSn | geit | goat | 2 | 14% |
| DID-G | TIS | NSn | haak | hook | 2 | 14% |
| DID-G | TIS | NSn | haken | hooks | 2 | 14% |
| DID-G | NIS | NSn | handen | hands | 2 | 14% |
| DID-G | TIS | NSn | handen | hands | 2 | 14% |
| DID-G | TIS | NSn | hysterie | hysteria | 2 | 14% |
| DID-G | TIS | NSn | instorten | to collapse | 2 | 14% |
| DID-G | NIS | NSn | kader | framework | 2 | 14% |
| DID-G | TIS | NSn | kader | framework | 2 | 14% |
| DID-G | NIS | NSn | kou | cold | 2 | 14% |
| DID-G | NIS | NSn | kreunen | to moan | 2 | 14% |
| DID-G | TIS | NSn | legpuzzel | jigsaw puzzle | 2 | 14% |
| DID-G | NIS | NSn | lepra | leprosy | 2 | 14% |
| DID-G | NIS | NSn | likken | to lick | 2 | 14% |
| DID-G | TIS | NSn | middel | middle | 2 | 14% |
| DID-G | NIS | NSn | misleiden | to deceive | 2 | 14% |
| DID-G | TIS | NSn | misvormen | to deform | 2 | 14% |
| DID-G | NIS | NSn | moedeloos | despondent | 2 | 14% |
| DID-G | TIS | NSn | moord | murder | 2 | 14% |
| DID-G | NIS | NSn | octaaf | octave | 2 | 14% |
| DID-G | TIS | NSn | onmacht | powerlessness | 2 | 14% |
| DID-G | NIS | NSn | ontrouw | unfaithful | 2 | 14% |
| DID-G | NIS | NSn | oom | uncle | 2 | 14% |
| DID-G | TIS | NSn | oplichten | to scam | 2 | 14% |
| DID-G | NIS | NSn | orgie | orgy | 2 | 14% |
| DID-G | NIS | NSn | potlood | pencil | 2 | 14% |
| DID-G | NIS | NSn | snijden | to cut | 2 | 14% |
| DID-G | TIS | NSn | stank | stench | 2 | 14% |
| DID-G | TIS | NSn | steekwond | stab wound | 2 | 14% |
| DID-G | TIS | NSn | steil | steep | 2 | 14% |
| DID-G | TIS | NSn | stikken | to suffocate | 2 | 14% |
| DID-G | NIS | NSn | stinken | to stink | 2 | 14% |
| DID-G | TIS | NSn | stoel | chair | 2 | 14% |
| DID-G | NIS | NSn | stoep | sidewalk | 2 | 14% |
| DID-G | NIS | NSn | takken | branches | 2 | 14% |
| DID-G | TIS | NSn | tegenslag | setback | 2 | 14% |
| DID-G | TIS | NSn | tiran | tyrant | 2 | 14% |
| DID-G | TIS | NSn | uitjouwen | to boo | 2 | 14% |
| DID-G | NIS | NSn | uitlachen | to laugh at | 2 | 14% |
| DID-G | NIS | NSn | verdord | withered | 2 | 14% |
| DID-G | TIS | NSn | verdord | withered | 2 | 14% |
| DID-G | TIS | NSn | woest | enraged | 2 | 14% |
| DID-G | NIS | NSn | zeer | ache | 2 | 14% |
| DID-G | NIS | NSn | zuigen | to suck | 2 | 14% |
| DID-G | NIS | St | achterlaten | leave behind | 1 | 7% |
| DID-G | NIS | St | afblaffen | to bark at | 1 | 7% |
| DID-G | NIS | St | afgrijzen | horror | 1 | 7% |
| DID-G | TIS | St | afscheid | goodbye | 1 | 7% |
| DID-G | TIS | St | afschuw | revulsion | 1 | 7% |
| DID-G | TIS | St | baby | baby | 1 | 7% |
| DID-G | NIS | St | bedrog | deceit | 1 | 7% |
| DID-G | TIS | St | bedrog | deceit | 1 | 7% |
| DID-G | NIS | St | besmetten | to contaminate | 1 | 7% |
| DID-G | NIS | St | blinddoek | blindfold | 1 | 7% |
| DID-G | TIS | St | blinddoek | blindfold | 1 | 7% |
| DID-G | TIS | St | bloot | naked | 1 | 7% |
| DID-G | NIS | St | broer | brother | 1 | 7% |
| DID-G | NIS | St | chauffeur | driver | 1 | 7% |
| DID-G | TIS | St | cirkel | circle | 1 | 7% |
| DID-G | NIS | St | crimineel | criminal | 1 | 7% |
| DID-G | TIS | St | depressie | depression | 1 | 7% |
| DID-G | TIS | St | doden | to kill | 1 | 7% |
| DID-G | TIS | St | dreigen | to threaten | 1 | 7% |
| DID-G | NIS | St | droevig | sad | 1 | 7% |
| DID-G | TIS | St | dubbel | double | 1 | 7% |
| DID-G | NIS | St | elleboog | elbow | 1 | 7% |
| DID-G | TIS | St | embryo | embryo | 1 | 7% |
| DID-G | TIS | St | ergeren | to annoy | 1 | 7% |
| DID-G | TIS | St | ergernis | annoyance | 1 | 7% |
| DID-G | NIS | St | getreiter | harassment | 1 | 7% |
| DID-G | NIS | St | gezwel | tumor | 1 | 7% |
| DID-G | TIS | St | gezwel | tumor | 1 | 7% |
| DID-G | NIS | St | gijzeling | kidnapping | 1 | 7% |
| DID-G | TIS | St | gijzeling | kidnapping | 1 | 7% |
| DID-G | TIS | St | ijzer | iron | 1 | 7% |
| DID-G | TIS | St | kanker | cancer | 1 | 7% |
| DID-G | TIS | St | kerk | church | 1 | 7% |
| DID-G | NIS | St | knijpen | to pinch | 1 | 7% |
| DID-G | TIS | St | knijpen | to pinch | 1 | 7% |
| DID-G | NIS | St | kogel | bullet | 1 | 7% |
| DID-G | NIS | St | kreng | bitch | 1 | 7% |
| DID-G | NIS | St | krenken | to hurt | 1 | 7% |
| DID-G | TIS | St | krenken | to hurt | 1 | 7% |
| DID-G | NIS | St | lafaard | coward | 1 | 7% |
| DID-G | TIS | St | lawaai | noise | 1 | 7% |
| DID-G | NIS | St | leugen | lie | 1 | 7% |
| DID-G | TIS | St | liegen | to lie | 1 | 7% |
| DID-G | NIS | St | mes | knife | 1 | 7% |
| DID-G | TIS | St | mes | knife | 1 | 7% |
| DID-G | TIS | St | middel | middle | 1 | 7% |
| DID-G | TIS | St | miskraam | miscarriage | 1 | 7% |
| DID-G | TIS | St | misleiden | to deceive | 1 | 7% |
| DID-G | TIS | St | mislukt | failed | 1 | 7% |
| DID-G | TIS | St | mismaakt | deformed | 1 | 7% |
| DID-G | TIS | St | moedeloos | despondent | 1 | 7% |
| DID-G | TIS | St | moord | murder | 1 | 7% |
| DID-G | NIS | St | noodkreet | cry for help | 1 | 7% |
| DID-G | TIS | St | noodkreet | cry for help | 1 | 7% |
| DID-G | NIS | St | ogen | eyes | 1 | 7% |
| DID-G | TIS | St | ogen | eyes | 1 | 7% |
| DID-G | TIS | St | omkomen | to perish | 1 | 7% |
| DID-G | NIS | St | ongeluk | accident | 1 | 7% |
| DID-G | NIS | St | ongeval | accident | 1 | 7% |
| DID-G | TIS | St | ontrouw | unfaithful | 1 | 7% |
| DID-G | NIS | St | ontslag | resignation or dismissal | 1 | 7% |
| DID-G | NIS | St | oorlog | war | 1 | 7% |
| DID-G | NIS | St | oplichten | to scam | 1 | 7% |
| DID-G | NIS | St | orgie | orgy | 1 | 7% |
| DID-G | TIS | St | orgie | orgy | 1 | 7% |
| DID-G | NIS | St | plafond | ceiling | 1 | 7% |
| DID-G | TIS | St | plafond | ceiling | 1 | 7% |
| DID-G | NIS | St | rad | wheel | 1 | 7% |
| DID-G | TIS | St | razernij | fury | 1 | 7% |
| DID-G | NIS | St | regel | rule | 1 | 7% |
| DID-G | TIS | St | regel | rule | 1 | 7% |
| DID-G | NIS | St | roofmoord | robbery with murder | 1 | 7% |
| DID-G | TIS | St | roofmoord | robbery with murder | 1 | 7% |
| DID-G | NIS | St | schande | shame | 1 | 7% |
| DID-G | TIS | St | schande | shame | 1 | 7% |
| DID-G | TIS | St | schoft | bastard | 1 | 7% |
| DID-G | TIS | St | schok | shock | 1 | 7% |
| DID-G | NIS | St | schoppen | to kick | 1 | 7% |
| DID-G | TIS | St | slijmen | suck up to | 1 | 7% |
| DID-G | NIS | St | spoelen | to flush or to rinse | 1 | 7% |
| DID-G | NIS | St | steekwond | stab wound | 1 | 7% |
| DID-G | NIS | St | sterven | to die | 1 | 7% |
| DID-G | NIS | St | stinken | to stink | 1 | 7% |
| DID-G | NIS | St | stoelpoot | chair leg | 1 | 7% |
| DID-G | TIS | St | tegenslag | setback | 1 | 7% |
| DID-G | TIS | St | treden | to step | 1 | 7% |
| DID-G | NIS | St | uitkleden | to undress | 1 | 7% |
| DID-G | TIS | St | uitkleden | to undress | 1 | 7% |
| DID-G | NIS | St | uitlachen | to laugh at | 1 | 7% |
| DID-G | TIS | St | verbranden | to burn | 1 | 7% |
| DID-G | TIS | St | verdord | withered | 1 | 7% |
| DID-G | NIS | St | vergroten | enlarge | 1 | 7% |
| DID-G | NIS | St | vernielen | to vandalize | 1 | 7% |
| DID-G | NIS | St | vierkant | square | 1 | 7% |
| DID-G | NIS | St | wraak | revenge | 1 | 7% |
| DID-G | TIS | St | zeuren | to nag | 1 | 7% |
| DID-G | NIS | St | zondebok | scapegoat | 1 | 7% |
| DID-G | NIS | St | zuigen | to suck | 1 | 7% |
| DID-G | TIS | NSt | absorptie | absorption | 1 | 7% |
| DID-G | NIS | NSt | afgrijzen | horror | 1 | 7% |
| DID-G | TIS | NSt | afgrijzen | horror | 1 | 7% |
| DID-G | NIS | NSt | afhakken | to chop off | 1 | 7% |
| DID-G | NIS | NSt | afkeer | aversion | 1 | 7% |
| DID-G | TIS | NSt | afkraken | to decry | 1 | 7% |
| DID-G | NIS | NSt | afwijzing | rejection | 1 | 7% |
| DID-G | TIS | NSt | agressie | aggression | 1 | 7% |
| DID-G | NIS | NSt | baby | baby | 1 | 7% |
| DID-G | NIS | NSt | bang | afraid | 1 | 7% |
| DID-G | TIS | NSt | bedreigen | to threaten | 1 | 7% |
| DID-G | NIS | NSt | begraven | to bury | 1 | 7% |
| DID-G | TIS | NSt | begraven | to bury | 1 | 7% |
| DID-G | NIS | NSt | beklemmen | to oppress | 1 | 7% |
| DID-G | TIS | NSt | beklemmen | to oppress | 1 | 7% |
| DID-G | NIS | NSt | belazerd | fooled | 1 | 7% |
| DID-G | TIS | NSt | belazerd | fooled | 1 | 7% |
| DID-G | TIS | NSt | blinddoek | blindfold | 1 | 7% |
| DID-G | NIS | NSt | bloot | naked | 1 | 7% |
| DID-G | TIS | NSt | chanteren | to blackmail | 1 | 7% |
| DID-G | NIS | NSt | chauffeur | driver | 1 | 7% |
| DID-G | NIS | NSt | conflict | conflict | 1 | 7% |
| DID-G | TIS | NSt | depressie | depression | 1 | 7% |
| DID-G | NIS | NSt | deurknop | door knob | 1 | 7% |
| DID-G | TIS | NSt | doden | to kill | 1 | 7% |
| DID-G | NIS | NSt | domkop | idiot | 1 | 7% |
| DID-G | NIS | NSt | doodgaan | to die | 1 | 7% |
| DID-G | NIS | NSt | doorslikken | to swallow | 1 | 7% |
| DID-G | TIS | NSt | droefheid | sadness | 1 | 7% |
| DID-G | TIS | NSt | droevig | sad | 1 | 7% |
| DID-G | NIS | NSt | dwingen | to coerce or to force | 1 | 7% |
| DID-G | NIS | NSt | elleboog | elbow | 1 | 7% |
| DID-G | NIS | NSt | ellende | misery | 1 | 7% |
| DID-G | NIS | NSt | ergeren | to annoy | 1 | 7% |
| DID-G | TIS | NSt | ergeren | to annoy | 1 | 7% |
| DID-G | NIS | NSt | ergernis | annoyance | 1 | 7% |
| DID-G | TIS | NSt | firma | firm | 1 | 7% |
| DID-G | NIS | NSt | gaatje | little hole | 1 | 7% |
| DID-G | TIS | NSt | getreiter | harassment | 1 | 7% |
| DID-G | TIS | NSt | geweld | violence | 1 | 7% |
| DID-G | NIS | NSt | haak | hook | 1 | 7% |
| DID-G | TIS | NSt | haak | hook | 1 | 7% |
| DID-G | TIS | NSt | hagedis | lizard | 1 | 7% |
| DID-G | NIS | NSt | haken | hooks | 1 | 7% |
| DID-G | NIS | NSt | handen | hands | 1 | 7% |
| DID-G | TIS | NSt | ivoor | ivory | 1 | 7% |
| DID-G | NIS | NSt | kerk | church | 1 | 7% |
| DID-G | NIS | NSt | kogel | bullet | 1 | 7% |
| DID-G | TIS | NSt | kogel | bullet | 1 | 7% |
| DID-G | NIS | NSt | krabben | to scratch | 1 | 7% |
| DID-G | TIS | NSt | kreng | bitch | 1 | 7% |
| DID-G | TIS | NSt | kreunen | to moan | 1 | 7% |
| DID-G | NIS | NSt | lafaard | coward | 1 | 7% |
| DID-G | TIS | NSt | lawaai | noise | 1 | 7% |
| DID-G | NIS | NSt | legpuzzel | jigsaw puzzle | 1 | 7% |
| DID-G | NIS | NSt | leugen | lie | 1 | 7% |
| DID-G | TIS | NSt | lijden | to suffer | 1 | 7% |
| DID-G | TIS | NSt | likken | to lick | 1 | 7% |
| DID-G | TIS | NSt | man | man | 1 | 7% |
| DID-G | TIS | NSt | masker | mask | 1 | 7% |
| DID-G | TIS | NSt | mes | knife | 1 | 7% |
| DID-G | TIS | NSt | miskraam | miscarriage | 1 | 7% |
| DID-G | TIS | NSt | misleiden | to deceive | 1 | 7% |
| DID-G | TIS | NSt | mismaakt | deformed | 1 | 7% |
| DID-G | NIS | NSt | misvormen | to deform | 1 | 7% |
| DID-G | TIS | NSt | misvormen | to deform | 1 | 7% |
| DID-G | TIS | NSt | moedeloos | despondent | 1 | 7% |
| DID-G | TIS | NSt | moeder | mother | 1 | 7% |
| DID-G | TIS | NSt | moord | murder | 1 | 7% |
| DID-G | TIS | NSt | ongeluk | accident | 1 | 7% |
| DID-G | NIS | NSt | ongeval | accident | 1 | 7% |
| DID-G | NIS | NSt | ongewenst | unwanted | 1 | 7% |
| DID-G | NIS | NSt | onmacht | powerlessness | 1 | 7% |
| DID-G | TIS | NSt | ontslag | resignation or dismissal | 1 | 7% |
| DID-G | NIS | NSt | plafond | ceiling | 1 | 7% |
| DID-G | NIS | NSt | plank | shelf | 1 | 7% |
| DID-G | NIS | NSt | rad | wheel | 1 | 7% |
| DID-G | TIS | NSt | razernij | fury | 1 | 7% |
| DID-G | TIS | NSt | regel | rule | 1 | 7% |
| DID-G | NIS | NSt | ruzie | fight or quarrel | 1 | 7% |
| DID-G | NIS | NSt | schaden | to damage | 1 | 7% |
| DID-G | TIS | NSt | schande | shame | 1 | 7% |
| DID-G | NIS | NSt | schoft | bastard | 1 | 7% |
| DID-G | NIS | NSt | schok | shock | 1 | 7% |
| DID-G | TIS | NSt | schok | shock | 1 | 7% |
| DID-G | NIS | NSt | schoppen | to kick | 1 | 7% |
| DID-G | TIS | NSt | schuldig | guilty | 1 | 7% |
| DID-G | NIS | NSt | slecht | bad | 1 | 7% |
| DID-G | NIS | NSt | spoelen | to flush or to rinse | 1 | 7% |
| DID-G | NIS | NSt | sterven | to die | 1 | 7% |
| DID-G | TIS | NSt | sterven | to die | 1 | 7% |
| DID-G | TIS | NSt | stinken | to stink | 1 | 7% |
| DID-G | NIS | NSt | stoep | sidewalk | 1 | 7% |
| DID-G | NIS | NSt | teller | counter | 1 | 7% |
| DID-G | TIS | NSt | trappen | to kick | 1 | 7% |
| DID-G | NIS | NSt | trauma | trauma | 1 | 7% |
| DID-G | TIS | NSt | treiteren | to harass | 1 | 7% |
| DID-G | NIS | NSt | uitgescholden | scolded | 1 | 7% |
| DID-G | TIS | NSt | uitgescholden | scolded | 1 | 7% |
| DID-G | NIS | NSt | uitlachen | to laugh at | 1 | 7% |
| DID-G | NIS | NSt | vastbinden | to tie | 1 | 7% |
| DID-G | NIS | NSt | verbranden | to burn | 1 | 7% |
| DID-G | TIS | NSt | verdrinken | to drown | 1 | 7% |
| DID-G | TIS | NSt | verkrachting | rape | 1 | 7% |
| DID-G | TIS | NSt | verlammen | to paralyze | 1 | 7% |
| DID-G | TIS | NSt | vernielen | to vandalize | 1 | 7% |
| DID-G | TIS | NSt | verzuipen | to drown | 1 | 7% |
| DID-G | TIS | NSt | vreemde | stranger | 1 | 7% |
| DID-G | TIS | NSt | walging | disgust | 1 | 7% |
| DID-G | NIS | NSt | wanhopen | to despair | 1 | 7% |
| DID-G | NIS | NSt | woest | enraged | 1 | 7% |
| DID-G | NIS | NSt | zeuren | to nag | 1 | 7% |
| DID-G | TIS | NSt | zeuren | to nag | 1 | 7% |
| DID-G | NIS | NSt | zondebok | scapegoat | 1 | 7% |
| DID-G | NIS | NSt | zuigen | to suck | 1 | 7% |
| DID-G | TIS | NSt | zuigen | to suck | 1 | 7% |
| DID-G | NIS | NSn | aanslag | attack | 1 | 7% |
| DID-G | NIS | NSn | achterlaten | leave behind | 1 | 7% |
| DID-G | NIS | NSn | afblaffen | to bark at | 1 | 7% |
| DID-G | TIS | NSn | afblaffen | to bark at | 1 | 7% |
| DID-G | NIS | NSn | afgunst | envy | 1 | 7% |
| DID-G | TIS | NSn | afkeer | aversion | 1 | 7% |
| DID-G | TIS | NSn | afkraken | to decry | 1 | 7% |
| DID-G | NIS | NSn | afscheid | goodbye | 1 | 7% |
| DID-G | TIS | NSn | afschuw | revulsion | 1 | 7% |
| DID-G | NIS | NSn | alleen | alone | 1 | 7% |
| DID-G | NIS | NSn | armoede | poverty | 1 | 7% |
| DID-G | TIS | NSn | armoede | poverty | 1 | 7% |
| DID-G | NIS | NSn | atoombom | nuclear bomb | 1 | 7% |
| DID-G | TIS | NSn | baby | baby | 1 | 7% |
| DID-G | NIS | NSn | bang | afraid | 1 | 7% |
| DID-G | NIS | NSn | bed | bed | 1 | 7% |
| DID-G | TIS | NSn | bed | bed | 1 | 7% |
| DID-G | NIS | NSn | bedrog | deceit | 1 | 7% |
| DID-G | TIS | NSn | begraven | to bury | 1 | 7% |
| DID-G | NIS | NSn | beklemmen | to oppress | 1 | 7% |
| DID-G | TIS | NSn | beklemmen | to oppress | 1 | 7% |
| DID-G | TIS | NSn | blinddoek | blindfold | 1 | 7% |
| DID-G | TIS | NSn | bloot | naked | 1 | 7% |
| DID-G | NIS | NSn | bordeel | brothel | 1 | 7% |
| DID-G | TIS | NSn | bordeel | brothel | 1 | 7% |
| DID-G | NIS | NSn | branden | to burn | 1 | 7% |
| DID-G | NIS | NSn | broer | brother | 1 | 7% |
| DID-G | NIS | NSn | buurman | neighbor | 1 | 7% |
| DID-G | TIS | NSn | buurman | neighbor | 1 | 7% |
| DID-G | NIS | NSn | chanteren | to blackmail | 1 | 7% |
| DID-G | TIS | NSn | conflict | conflict | 1 | 7% |
| DID-G | NIS | NSn | crimineel | criminal | 1 | 7% |
| DID-G | TIS | NSn | diefstal | theft | 1 | 7% |
| DID-G | TIS | NSn | dokter | (general practice) doctor | 1 | 7% |
| DID-G | NIS | NSn | domkop | idiot | 1 | 7% |
| DID-G | TIS | NSn | domkop | idiot | 1 | 7% |
| DID-G | NIS | NSn | doodgaan | to die | 1 | 7% |
| DID-G | NIS | NSn | doodsteek | deathblow | 1 | 7% |
| DID-G | NIS | NSn | doorslikken | to swallow | 1 | 7% |
| DID-G | NIS | NSn | dubbel | double | 1 | 7% |
| DID-G | NIS | NSn | dwingen | to coerce or to force | 1 | 7% |
| DID-G | TIS | NSn | ergeren | to annoy | 1 | 7% |
| DID-G | NIS | NSn | ergernis | annoyance | 1 | 7% |
| DID-G | NIS | NSn | etter | pus | 1 | 7% |
| DID-G | NIS | NSn | falen | to fail | 1 | 7% |
| DID-G | NIS | NSn | gaatje | little hole | 1 | 7% |
| DID-G | TIS | NSn | gezwel | tumor | 1 | 7% |
| DID-G | NIS | NSn | gijzeling | kidnapping | 1 | 7% |
| DID-G | NIS | NSn | haken | hooks or to hook | 1 | 7% |
| DID-G | NIS | NSn | haten | to hate | 1 | 7% |
| DID-G | TIS | NSn | huisdier | pet | 1 | 7% |
| DID-G | NIS | NSn | hysterie | hysteria | 1 | 7% |
| DID-G | NIS | NSn | inbraak | burglary | 1 | 7% |
| DID-G | TIS | NSn | inbraak | burglary | 1 | 7% |
| DID-G | NIS | NSn | incest | incest | 1 | 7% |
| DID-G | NIS | NSn | kelder | basement | 1 | 7% |
| DID-G | TIS | NSn | kelder | basement | 1 | 7% |
| DID-G | TIS | NSn | knijpen | to pinch | 1 | 7% |
| DID-G | TIS | NSn | kogel | bullet | 1 | 7% |
| DID-G | TIS | NSn | kozijn | window frame | 1 | 7% |
| DID-G | TIS | NSn | krabben | to scratch | 1 | 7% |
| DID-G | TIS | NSn | kreng | bitch | 1 | 7% |
| DID-G | NIS | NSn | krenken | to hurt | 1 | 7% |
| DID-G | TIS | NSn | krenken | to hurt | 1 | 7% |
| DID-G | NIS | NSn | kwetsen | to hurt | 1 | 7% |
| DID-G | NIS | NSn | lawaai | noise | 1 | 7% |
| DID-G | NIS | NSn | liegen | to lie | 1 | 7% |
| DID-G | NIS | NSn | man | man | 1 | 7% |
| DID-G | TIS | NSn | martelen | to torture | 1 | 7% |
| DID-G | NIS | NSn | mes | knife | 1 | 7% |
| DID-G | TIS | NSn | mes | knife | 1 | 7% |
| DID-G | TIS | NSn | misdrijf | crime | 1 | 7% |
| DID-G | NIS | NSn | miskraam | miscarriage | 1 | 7% |
| DID-G | TIS | NSn | misleiden | to deceive | 1 | 7% |
| DID-G | TIS | NSn | mislukt | failed | 1 | 7% |
| DID-G | TIS | NSn | mismaakt | deformed | 1 | 7% |
| DID-G | NIS | NSn | nacht | night | 1 | 7% |
| DID-G | NIS | NSn | omkomen | to perish | 1 | 7% |
| DID-G | TIS | NSn | omkomen | to perish | 1 | 7% |
| DID-G | NIS | NSn | ongeluk | accident | 1 | 7% |
| DID-G | TIS | NSn | ongeluk | accident | 1 | 7% |
| DID-G | NIS | NSn | ongeval | accident | 1 | 7% |
| DID-G | TIS | NSn | ongeval | accident | 1 | 7% |
| DID-G | NIS | NSn | onzeker | uncertain | 1 | 7% |
| DID-G | TIS | NSn | oorlog | war | 1 | 7% |
| DID-G | TIS | NSn | orgie | orgy | 1 | 7% |
| DID-G | TIS | NSn | pedofiel | paedophile | 1 | 7% |
| DID-G | NIS | NSn | piekeren | to mull | 1 | 7% |
| DID-G | TIS | NSn | regel | rule | 1 | 7% |
| DID-G | TIS | NSn | roofmoord | robbery with murder | 1 | 7% |
| DID-G | NIS | NSn | ruzie | fight or quarrel | 1 | 7% |
| DID-G | TIS | NSn | sadist | sadist | 1 | 7% |
| DID-G | TIS | NSn | schaden | to damage | 1 | 7% |
| DID-G | TIS | NSn | schoft | bastard | 1 | 7% |
| DID-G | NIS | NSn | schok | shock | 1 | 7% |
| DID-G | NIS | NSn | schoppen | to kick | 1 | 7% |
| DID-G | NIS | NSn | slet | slut | 1 | 7% |
| DID-G | NIS | NSn | snauwen | to snarl | 1 | 7% |
| DID-G | TIS | NSn | snijden | to cut | 1 | 7% |
| DID-G | TIS | NSn | spoelen | to flush or to rinse | 1 | 7% |
| DID-G | NIS | NSn | stank | stench | 1 | 7% |
| DID-G | NIS | NSn | steekwond | stab wound | 1 | 7% |
| DID-G | TIS | NSn | sterven | to die | 1 | 7% |
| DID-G | NIS | NSn | stiekem | secretly | 1 | 7% |
| DID-G | NIS | NSn | stikken | to suffocate | 1 | 7% |
| DID-G | TIS | NSn | trauma | trauma | 1 | 7% |
| DID-G | NIS | NSn | uitjouwen | to boo | 1 | 7% |
| DID-G | NIS | NSn | uitkleden | to undress | 1 | 7% |
| DID-G | TIS | NSn | uitlachen | to laugh at | 1 | 7% |
| DID-G | NIS | NSn | vader | father | 1 | 7% |
| DID-G | NIS | NSn | verdrinken | to drown | 1 | 7% |
| DID-G | NIS | NSn | verleidster | temptress | 1 | 7% |
| DID-G | TIS | NSn | verleidster | temptress | 1 | 7% |
| DID-G | TIS | NSn | verraad | betrayal | 1 | 7% |
| DID-G | TIS | NSn | versie | version | 1 | 7% |
| DID-G | NIS | NSn | vuur | fire | 1 | 7% |
| DID-G | TIS | NSn | vuur | fire | 1 | 7% |
| DID-G | TIS | NSn | wraak | revenge | 1 | 7% |
| DID-G | TIS | NSn | wurgen | to strangle | 1 | 7% |
| DID-G | TIS | NSn | zeuren | to nag | 1 | 7% |
| DID-G | NIS | NSn | zondebok | scapegoat | 1 | 7% |
| DID-G | TIS | NSn | zondebok | scapegoat | 1 | 7% |
| DID-S^^n2^ | NIS | NSt | verkrachting | rape | 14 | 100% |
| DID-S | TIS | NSn | tijdperk | era | 11 | 79% |
| DID-S | TIS | St | verkrachting | rape | 10 | 71% |
| DID-S | TIS | St | walging | disgust | 10 | 71% |
| DID-S | NIS | NSt | bloedbad | bloodbath | 10 | 71% |
| DID-S | NIS | NSt | crimineel | criminal | 10 | 71% |
| DID-S | NIS | NSt | incest | incest | 10 | 71% |
| DID-S | TIS | NSt | kanker | cancer | 10 | 71% |
| DID-S | NIS | NSt | verdrinken | to drown | 10 | 71% |
| DID-S | TIS | NSn | grondstof | natural resource | 10 | 71% |
| DID-S | NIS | NSn | schroef | screw | 10 | 71% |
| DID-S | TIS | NSn | telegram | telegram | 10 | 71% |
| DID-S | NIS | St | afwijzing | rejection | 9 | 64% |
| DID-S | TIS | St | geweld | violence | 9 | 64% |
| DID-S | NIS | NSt | folteren | to torture | 9 | 64% |
| DID-S | NIS | NSt | geweld | violence | 9 | 64% |
| DID-S | NIS | NSt | martelen | to torture | 9 | 64% |
| DID-S | NIS | NSt | messteek | knife stab | 9 | 64% |
| DID-S | NIS | NSt | miskraam | miscarriage | 9 | 64% |
| DID-S | TIS | NSt | oorlog | war | 9 | 64% |
| DID-S | NIS | NSt | pedofiel | paedophile | 9 | 64% |
| DID-S | NIS | NSn | grondstof | natural resource | 9 | 64% |
| DID-S | TIS | NSn | tapijt | tapestry | 9 | 64% |
| DID-S | TIS | NSn | theelepel | teaspoon | 9 | 64% |
| DID-S | NIS | NSn | tijdperk | era | 9 | 64% |
| DID-S | TIS | NSn | trottoir | pavement | 9 | 64% |
| DID-S | TIS | NSn | zandloper | hourglass | 9 | 64% |
| DID-S | NIS | St | angst | fear | 8 | 57% |
| DID-S | TIS | St | bang | afraid | 8 | 57% |
| DID-S | TIS | St | doorslikken | to swallow | 8 | 57% |
| DID-S | TIS | St | dwang | coercion or force | 8 | 57% |
| DID-S | TIS | St | dwingen | to coerce or to force | 8 | 57% |
| DID-S | NIS | St | onmacht | powerlessness | 8 | 57% |
| DID-S | NIS | St | onrecht | injustice | 8 | 57% |
| DID-S | TIS | NSt | bloedbad | bloodbath | 8 | 57% |
| DID-S | NIS | NSt | doodslag | manslaughter | 8 | 57% |
| DID-S | NIS | NSt | gezwel | tumor | 8 | 57% |
| DID-S | NIS | NSt | kogel | bullet | 8 | 57% |
| DID-S | NIS | NSt | misdrijf | crime | 8 | 57% |
| DID-S | NIS | NSt | omkomen | to perish | 8 | 57% |
| DID-S | NIS | NSt | oorlog | war | 8 | 57% |
| DID-S | NIS | NSt | slet | slut | 8 | 57% |
| DID-S | TIS | NSt | verdrinken | to drown | 8 | 57% |
| DID-S | TIS | NSt | verzuipen | to drown | 8 | 57% |
| DID-S | NIS | NSn | plank | shelf | 8 | 57% |
| DID-S | TIS | NSn | plank | shelf | 8 | 57% |
| DID-S | TIS | NSn | rad | wheel | 8 | 57% |
| DID-S | NIS | NSn | register | register | 8 | 57% |
| DID-S | NIS | NSn | stukadoor | plasterer | 8 | 57% |
| DID-S | NIS | NSn | trede | step | 8 | 57% |
| DID-S | NIS | NSn | zandloper | hourglass | 8 | 57% |
| DID-S | TIS | NSn | zegel | seal | 8 | 57% |
| DID-S | TIS | St | afkeer | aversion | 7 | 50% |
| DID-S | TIS | St | haten | to hate | 7 | 50% |
| DID-S | NIS | St | leugen | lie | 7 | 50% |
| DID-S | TIS | St | lijden | to suffer | 7 | 50% |
| DID-S | TIS | St | man | man | 7 | 50% |
| DID-S | TIS | St | ongewenst | unwanted | 7 | 50% |
| DID-S | TIS | St | onmacht | powerlessness | 7 | 50% |
| DID-S | TIS | St | onrecht | injustice | 7 | 50% |
| DID-S | NIS | St | onzeker | uncertain | 7 | 50% |
| DID-S | TIS | St | pijn | pain | 7 | 50% |
| DID-S | TIS | St | slecht | bad | 7 | 50% |
| DID-S | TIS | St | verlammen | to paralyze | 7 | 50% |
| DID-S | TIS | St | zuigen | to suck | 7 | 50% |
| DID-S | TIS | NSt | aanslag | attack | 7 | 50% |
| DID-S | NIS | NSt | afgrijzen | horror | 7 | 50% |
| DID-S | TIS | NSt | crimineel | criminal | 7 | 50% |
| DID-S | TIS | NSt | gijzeling | kidnapping | 7 | 50% |
| DID-S | NIS | NSt | haten | to hate | 7 | 50% |
| DID-S | NIS | NSt | kanker | cancer | 7 | 50% |
| DID-S | NIS | NSt | moord | murder | 7 | 50% |
| DID-S | NIS | NSt | oplichten | to scam | 7 | 50% |
| DID-S | TIS | NSt | pedofiel | paedophile | 7 | 50% |
| DID-S | TIS | NSt | roofmoord | robbery with murder | 7 | 50% |
| DID-S | NIS | NSt | sadist | sadist | 7 | 50% |
| DID-S | NIS | NSt | steekwond | stab wound | 7 | 50% |
| DID-S | TIS | NSt | verdord | withered | 7 | 50% |
| DID-S | NIS | NSt | verzuipen | to drown | 7 | 50% |
| DID-S | NIS | NSt | walging | disgust | 7 | 50% |
| DID-S | NIS | NSt | wraak | revenge | 7 | 50% |
| DID-S | NIS | NSt | wurgen | to strangle | 7 | 50% |
| DID-S | TIS | NSn | bestek | cutlery | 7 | 50% |
| DID-S | NIS | NSn | cirkel | circle | 7 | 50% |
| DID-S | NIS | NSn | consulaat | consulate | 7 | 50% |
| DID-S | NIS | NSn | dakgoot | gutter | 7 | 50% |
| DID-S | NIS | NSn | deurknop | door knob | 7 | 50% |
| DID-S | TIS | NSn | flacon | bottle or vial | 7 | 50% |
| DID-S | NIS | NSn | kenteken | license plate | 7 | 50% |
| DID-S | NIS | NSn | leuning | railing | 7 | 50% |
| DID-S | NIS | NSn | metselaar | bricklayer | 7 | 50% |
| DID-S | TIS | NSn | metselaar | bricklayer | 7 | 50% |
| DID-S | TIS | NSn | potlood | pencil | 7 | 50% |
| DID-S | TIS | NSn | roeren | to stir | 7 | 50% |
| DID-S | TIS | NSn | schroef | screw | 7 | 50% |
| DID-S | TIS | NSn | stukadoor | plasterer | 7 | 50% |
| DID-S | NIS | NSn | tegel | tile | 7 | 50% |
| DID-S | TIS | NSn | tegel | tile | 7 | 50% |
| DID-S | NIS | NSn | teller | counter | 7 | 50% |
| DID-S | TIS | NSn | trede | step | 7 | 50% |
| DID-S | NIS | NSn | trottoir | pavement | 7 | 50% |
| DID-S | TIS | NSn | uitgever | publisher | 7 | 50% |
| DID-S | TIS | NSn | vierkant | square | 7 | 50% |
| DID-S | TIS | St | afwijzing | rejection | 6 | 43% |
| DID-S | NIS | St | bang | afraid | 6 | 43% |
| DID-S | TIS | St | bedreigen | to threaten | 6 | 43% |
| DID-S | TIS | St | ellende | misery | 6 | 43% |
| DID-S | NIS | St | ergernis | annoyance | 6 | 43% |
| DID-S | NIS | St | falen | to fail | 6 | 43% |
| DID-S | TIS | St | hijgen | to pant | 6 | 43% |
| DID-S | TIS | St | incest | incest | 6 | 43% |
| DID-S | NIS | St | liegen | to lie | 6 | 43% |
| DID-S | TIS | St | likken | to lick | 6 | 43% |
| DID-S | NIS | St | mislukt | failed | 6 | 43% |
| DID-S | NIS | St | ontrouw | unfaithful | 6 | 43% |
| DID-S | TIS | St | pedofiel | paedophile | 6 | 43% |
| DID-S | TIS | St | schoppen | to kick | 6 | 43% |
| DID-S | NIS | St | schuldig | guilty | 6 | 43% |
| DID-S | TIS | St | schuldig | guilty | 6 | 43% |
| DID-S | NIS | St | snauwen | to snarl | 6 | 43% |
| DID-S | TIS | St | stiekem | secretly | 6 | 43% |
| DID-S | TIS | St | vader | father | 6 | 43% |
| DID-S | NIS | St | vies | dirty | 6 | 43% |
| DID-S | NIS | NSt | beroerte | stroke | 6 | 43% |
| DID-S | TIS | NSt | doodslag | manslaughter | 6 | 43% |
| DID-S | NIS | NSt | doodsteek | deathblow | 6 | 43% |
| DID-S | NIS | NSt | dwang | coercion or force | 6 | 43% |
| DID-S | TIS | NSt | folteren | to torture | 6 | 43% |
| DID-S | NIS | NSt | meeloper | opportunist | 6 | 43% |
| DID-S | TIS | NSt | messteek | knife stab | 6 | 43% |
| DID-S | TIS | NSt | miskraam | miscarriage | 6 | 43% |
| DID-S | TIS | NSt | moord | murder | 6 | 43% |
| DID-S | TIS | NSt | omkomen | to perish | 6 | 43% |
| DID-S | TIS | NSt | sadist | sadist | 6 | 43% |
| DID-S | TIS | NSt | slet | slut | 6 | 43% |
| DID-S | NIS | NSt | verlammen | to paralyze | 6 | 43% |
| DID-S | TIS | NSt | verleidster | temptress | 6 | 43% |
| DID-S | TIS | NSt | wanhoop | despair | 6 | 43% |
| DID-S | NIS | NSt | wreedheid | cruelty | 6 | 43% |
| DID-S | TIS | NSn | aanmaken | to prepare | 6 | 43% |
| DID-S | NIS | NSn | bestek | cutlery | 6 | 43% |
| DID-S | TIS | NSn | bladzijde | page | 6 | 43% |
| DID-S | NIS | NSn | blikje | can | 6 | 43% |
| DID-S | TIS | NSn | broer | brother | 6 | 43% |
| DID-S | TIS | NSn | citaat | quote | 6 | 43% |
| DID-S | TIS | NSn | dwerg | dwarf | 6 | 43% |
| DID-S | NIS | NSn | element | element | 6 | 43% |
| DID-S | NIS | NSn | etiket | label | 6 | 43% |
| DID-S | NIS | NSn | firma | firm | 6 | 43% |
| DID-S | TIS | NSn | firma | firm | 6 | 43% |
| DID-S | NIS | NSn | haak | hook | 6 | 43% |
| DID-S | TIS | NSn | haak | hook | 6 | 43% |
| DID-S | NIS | NSn | kelder | basement | 6 | 43% |
| DID-S | NIS | NSn | kozijn | window frame | 6 | 43% |
| DID-S | TIS | NSn | leuning | railing | 6 | 43% |
| DID-S | TIS | NSn | magazijn | warehouse | 6 | 43% |
| DID-S | NIS | NSn | paragraaf | paragraph | 6 | 43% |
| DID-S | TIS | NSn | plafond | ceiling | 6 | 43% |
| DID-S | TIS | NSn | regenton | rain barrel | 6 | 43% |
| DID-S | TIS | NSn | stomerij | drycleaner | 6 | 43% |
| DID-S | NIS | NSn | telegram | telegram | 6 | 43% |
| DID-S | TIS | NSn | teller | counter | 6 | 43% |
| DID-S | NIS | NSn | theelepel | teaspoon | 6 | 43% |
| DID-S | NIS | NSn | touw | rope | 6 | 43% |
| DID-S | NIS | NSn | treden | to step | 6 | 43% |
| DID-S | NIS | NSn | uitgever | publisher | 6 | 43% |
| DID-S | NIS | NSn | zakje | little bag | 6 | 43% |
| DID-S | TIS | St | afblaffen | to bark at | 5 | 36% |
| DID-S | NIS | St | afkraken | to decry | 5 | 36% |
| DID-S | NIS | St | afscheid | goodbye | 5 | 36% |
| DID-S | NIS | St | afsnauwen | to snap at | 5 | 36% |
| DID-S | NIS | St | alleen | alone | 5 | 36% |
| DID-S | TIS | St | angst | fear | 5 | 36% |
| DID-S | NIS | St | bedrieger | deceiver | 5 | 36% |
| DID-S | NIS | St | bedrog | deceit | 5 | 36% |
| DID-S | NIS | St | belazerd | fooled | 5 | 36% |
| DID-S | NIS | St | droefheid | sadness | 5 | 36% |
| DID-S | NIS | St | dwang | coercion or force | 5 | 36% |
| DID-S | NIS | St | dwingen | to coerce or to force | 5 | 36% |
| DID-S | TIS | St | kreunen | to moan | 5 | 36% |
| DID-S | NIS | St | kwetsen | to hurt | 5 | 36% |
| DID-S | TIS | St | kwetsen | to hurt | 5 | 36% |
| DID-S | NIS | St | lafaard | coward | 5 | 36% |
| DID-S | NIS | St | lawaai | noise | 5 | 36% |
| DID-S | TIS | St | leugen | lie | 5 | 36% |
| DID-S | TIS | St | mislukt | failed | 5 | 36% |
| DID-S | TIS | St | opsluiten | to lock up | 5 | 36% |
| DID-S | NIS | St | piekeren | to mull | 5 | 36% |
| DID-S | TIS | St | piemel | willy | 5 | 36% |
| DID-S | TIS | St | stikken | to suffocate | 5 | 36% |
| DID-S | TIS | St | tiran | tyrant | 5 | 36% |
| DID-S | TIS | St | trauma | trauma | 5 | 36% |
| DID-S | NIS | St | verdriet | sadness | 5 | 36% |
| DID-S | TIS | St | vies | dirty | 5 | 36% |
| DID-S | TIS | St | wreedheid | cruelty | 5 | 36% |
| DID-S | TIS | St | wurgen | to strangle | 5 | 36% |
| DID-S | NIS | St | zeuren | to nag | 5 | 36% |
| DID-S | NIS | NSt | aanslag | attack | 5 | 36% |
| DID-S | NIS | NSt | atoombom | nuclear bomb | 5 | 36% |
| DID-S | NIS | NSt | bedreigen | to threaten | 5 | 36% |
| DID-S | NIS | NSt | bedrieger | deceiver | 5 | 36% |
| DID-S | TIS | NSt | beroerte | stroke | 5 | 36% |
| DID-S | NIS | NSt | besmetten | to contaminate | 5 | 36% |
| DID-S | TIS | NSt | besmetten | to contaminate | 5 | 36% |
| DID-S | NIS | NSt | doden | to kill | 5 | 36% |
| DID-S | TIS | NSt | doodsteek | deathblow | 5 | 36% |
| DID-S | NIS | NSt | ellende | misery | 5 | 36% |
| DID-S | TIS | NSt | gezwel | tumor | 5 | 36% |
| DID-S | NIS | NSt | gijzeling | kidnapping | 5 | 36% |
| DID-S | TIS | NSt | hijgen | to pant | 5 | 36% |
| DID-S | TIS | NSt | incest | incest | 5 | 36% |
| DID-S | NIS | NSt | instorten | to collapse | 5 | 36% |
| DID-S | NIS | NSt | kotsen | to vomit | 5 | 36% |
| DID-S | NIS | NSt | lepra | leprosy | 5 | 36% |
| DID-S | TIS | NSt | lepra | leprosy | 5 | 36% |
| DID-S | NIS | NSt | lijden | to suffer | 5 | 36% |
| DID-S | TIS | NSt | martelen | to torture | 5 | 36% |
| DID-S | NIS | NSt | moedeloos | despondent | 5 | 36% |
| DID-S | TIS | NSt | orgie | orgy | 5 | 36% |
| DID-S | NIS | NSt | schoft | bastard | 5 | 36% |
| DID-S | NIS | NSt | stinken | to stink | 5 | 36% |
| DID-S | TIS | NSt | stinken | to stink | 5 | 36% |
| DID-S | TIS | NSt | zeuren | to nag | 5 | 36% |
| DID-S | NIS | NSn | absorptie | absorption | 5 | 36% |
| DID-S | TIS | NSn | cirkel | circle | 5 | 36% |
| DID-S | NIS | NSn | citaat | quote | 5 | 36% |
| DID-S | TIS | NSn | consulaat | consulate | 5 | 36% |
| DID-S | TIS | NSn | dakgoot | gutter | 5 | 36% |
| DID-S | TIS | NSn | deurknop | door knob | 5 | 36% |
| DID-S | NIS | NSn | dwerg | dwarf | 5 | 36% |
| DID-S | NIS | NSn | hertogin | duchess | 5 | 36% |
| DID-S | NIS | NSn | ijzer | iron | 5 | 36% |
| DID-S | TIS | NSn | ivoor | ivory | 5 | 36% |
| DID-S | TIS | NSn | kerk | church | 5 | 36% |
| DID-S | NIS | NSn | middel | middle | 5 | 36% |
| DID-S | NIS | NSn | omroep | broadcasting | 5 | 36% |
| DID-S | TIS | NSn | omroep | broadcasting | 5 | 36% |
| DID-S | TIS | NSn | ontslag | resignation or dismissal | 5 | 36% |
| DID-S | TIS | NSn | paragraaf | paragraph | 5 | 36% |
| DID-S | NIS | NSn | pilaar | pillar | 5 | 36% |
| DID-S | TIS | NSn | pilaar | pillar | 5 | 36% |
| DID-S | NIS | NSn | plafond | ceiling | 5 | 36% |
| DID-S | NIS | NSn | postzegel | stamp | 5 | 36% |
| DID-S | TIS | NSn | postzegel | stamp | 5 | 36% |
| DID-S | NIS | NSn | potlood | pencil | 5 | 36% |
| DID-S | NIS | NSn | rad | wheel | 5 | 36% |
| DID-S | NIS | NSn | schuren | to polish | 5 | 36% |
| DID-S | NIS | NSn | stoelpoot | chair leg | 5 | 36% |
| DID-S | TIS | NSn | takken | branches | 5 | 36% |
| DID-S | TIS | NSn | touw | rope | 5 | 36% |
| DID-S | TIS | NSn | traject | route | 5 | 36% |
| DID-S | TIS | NSn | treden | to step | 5 | 36% |
| DID-S | NIS | NSn | zegel | seal | 5 | 36% |
| DID-S | TIS | NSn | zetel | seat | 5 | 36% |
| DID-S | TIS | St | afgrijzen | horror | 4 | 29% |
| DID-S | NIS | St | afgunst | envy | 4 | 29% |
| DID-S | TIS | St | bedrieger | deceiver | 4 | 29% |
| DID-S | NIS | St | benauwen | to agitate | 4 | 29% |
| DID-S | TIS | St | branden | to burn | 4 | 29% |
| DID-S | NIS | St | droevig | sad | 4 | 29% |
| DID-S | NIS | St | ergeren | to annoy | 4 | 29% |
| DID-S | TIS | St | folteren | to torture | 4 | 29% |
| DID-S | TIS | St | kotsen | to vomit | 4 | 29% |
| DID-S | TIS | St | kreng | bitch | 4 | 29% |
| DID-S | TIS | St | martelen | to torture | 4 | 29% |
| DID-S | TIS | St | mismaakt | deformed | 4 | 29% |
| DID-S | TIS | St | noodkreet | cry for help | 4 | 29% |
| DID-S | NIS | St | ongewenst | unwanted | 4 | 29% |
| DID-S | NIS | St | pijn | pain | 4 | 29% |
| DID-S | NIS | St | ruzie | fight or quarrel | 4 | 29% |
| DID-S | TIS | St | schande | shame | 4 | 29% |
| DID-S | TIS | St | seks | sex | 4 | 29% |
| DID-S | TIS | St | slaaf | slave | 4 | 29% |
| DID-S | TIS | St | snauwen | to snarl | 4 | 29% |
| DID-S | TIS | St | trappen | to kick | 4 | 29% |
| DID-S | NIS | St | uitgescholden | scolded | 4 | 29% |
| DID-S | TIS | St | uitgescholden | scolded | 4 | 29% |
| DID-S | NIS | St | verraad | betrayal | 4 | 29% |
| DID-S | TIS | St | verraad | betrayal | 4 | 29% |
| DID-S | TIS | St | wanhoop | despair | 4 | 29% |
| DID-S | TIS | St | zeer | ache | 4 | 29% |
| DID-S | NIS | NSt | afblaffen | to bark at | 4 | 29% |
| DID-S | NIS | NSt | agressie | aggression | 4 | 29% |
| DID-S | NIS | NSt | armoede | poverty | 4 | 29% |
| DID-S | TIS | NSt | atoombom | nuclear bomb | 4 | 29% |
| DID-S | TIS | NSt | blinddoek | blindfold | 4 | 29% |
| DID-S | TIS | NSt | doden | to kill | 4 | 29% |
| DID-S | TIS | NSt | doodgaan | to die | 4 | 29% |
| DID-S | NIS | NSt | etter | pus | 4 | 29% |
| DID-S | NIS | NSt | hoer | whore | 4 | 29% |
| DID-S | NIS | NSt | kreng | bitch | 4 | 29% |
| DID-S | TIS | NSt | misdaad | crime | 4 | 29% |
| DID-S | TIS | NSt | mislukt | failed | 4 | 29% |
| DID-S | NIS | NSt | mismaakt | deformed | 4 | 29% |
| DID-S | NIS | NSt | noodkreet | cry for help | 4 | 29% |
| DID-S | TIS | NSt | oplichten | to scam | 4 | 29% |
| DID-S | NIS | NSt | orgie | orgy | 4 | 29% |
| DID-S | NIS | NSt | roofmoord | robbery with murder | 4 | 29% |
| DID-S | NIS | NSt | ruzie | fight or quarrel | 4 | 29% |
| DID-S | TIS | NSt | slijmen | suck up to | 4 | 29% |
| DID-S | TIS | NSt | steekwond | stab wound | 4 | 29% |
| DID-S | NIS | NSt | stikken | to suffocate | 4 | 29% |
| DID-S | NIS | NSt | tiran | tyrant | 4 | 29% |
| DID-S | NIS | NSt | treiteren | to harass | 4 | 29% |
| DID-S | NIS | NSt | uitjouwen | to boo | 4 | 29% |
| DID-S | TIS | NSt | verlammen | to paralyze | 4 | 29% |
| DID-S | NIS | NSt | vernielen | to vandalize | 4 | 29% |
| DID-S | NIS | NSt | wanhoop | despair | 4 | 29% |
| DID-S | NIS | NSn | aanmaken | to prepare | 4 | 29% |
| DID-S | NIS | NSn | blinddoek | blindfold | 4 | 29% |
| DID-S | TIS | NSn | chauffeur | driver | 4 | 29% |
| DID-S | NIS | NSn | document | document | 4 | 29% |
| DID-S | TIS | NSn | dozijn | dozen | 4 | 29% |
| DID-S | NIS | NSn | elleboog | elbow | 4 | 29% |
| DID-S | TIS | NSn | etiket | label | 4 | 29% |
| DID-S | NIS | NSn | flacon | bottle or vial | 4 | 29% |
| DID-S | NIS | NSn | gaatje | little hole | 4 | 29% |
| DID-S | TIS | NSn | gaatje | little hole | 4 | 29% |
| DID-S | NIS | NSn | gebouw | building | 4 | 29% |
| DID-S | TIS | NSn | gebouw | building | 4 | 29% |
| DID-S | NIS | NSn | hagedis | lizard | 4 | 29% |
| DID-S | NIS | NSn | haken | hooks | 4 | 29% |
| DID-S | NIS | NSn | handen | hands | 4 | 29% |
| DID-S | TIS | NSn | kader | framework | 4 | 29% |
| DID-S | TIS | NSn | kast | closet | 4 | 29% |
| DID-S | TIS | NSn | kenteken | license plate | 4 | 29% |
| DID-S | NIS | NSn | kerk | church | 4 | 29% |
| DID-S | TIS | NSn | metaal | metal | 4 | 29% |
| DID-S | TIS | NSn | ogen | eyes | 4 | 29% |
| DID-S | NIS | NSn | regenton | rain barrel | 4 | 29% |
| DID-S | NIS | NSn | roeren | to stir | 4 | 29% |
| DID-S | TIS | NSn | spoelen | to flush or to rinse | 4 | 29% |
| DID-S | TIS | NSn | steil | steep | 4 | 29% |
| DID-S | NIS | NSn | stoep | sidewalk | 4 | 29% |
| DID-S | TIS | NSn | stoep | sidewalk | 4 | 29% |
| DID-S | NIS | NSn | stomerij | drycleaner | 4 | 29% |
| DID-S | NIS | NSn | takken | branches | 4 | 29% |
| DID-S | NIS | NSn | tapijt | tapestry | 4 | 29% |
| DID-S | NIS | NSn | traject | route | 4 | 29% |
| DID-S | NIS | NSn | uitkleden | to undress | 4 | 29% |
| DID-S | NIS | NSn | verdord | withered | 4 | 29% |
| DID-S | NIS | NSn | versie | version | 4 | 29% |
| DID-S | NIS | NSn | vierkant | square | 4 | 29% |
| DID-S | NIS | NSn | vreemde | stranger | 4 | 29% |
| DID-S | TIS | NSn | zakje | little bag | 4 | 29% |
| DID-S | NIS | NSn | zetel | seat | 4 | 29% |
| DID-S | NIS | St | afgrijzen | horror | 3 | 21% |
| DID-S | NIS | St | afkeer | aversion | 3 | 21% |
| DID-S | TIS | St | afschuw | revulsion | 3 | 21% |
| DID-S | TIS | St | afsnauwen | to snap at | 3 | 21% |
| DID-S | TIS | St | alleen | alone | 3 | 21% |
| DID-S | TIS | St | bed | bed | 3 | 21% |
| DID-S | TIS | St | benauwen | to agitate | 3 | 21% |
| DID-S | TIS | St | besmetten | to contaminate | 3 | 21% |
| DID-S | TIS | St | bloot | naked | 3 | 21% |
| DID-S | TIS | St | chanteren | to blackmail | 3 | 21% |
| DID-S | NIS | St | conflict | conflict | 3 | 21% |
| DID-S | TIS | St | conflict | conflict | 3 | 21% |
| DID-S | NIS | St | domkop | idiot | 3 | 21% |
| DID-S | TIS | St | domkop | idiot | 3 | 21% |
| DID-S | NIS | St | dreigen | to threaten | 3 | 21% |
| DID-S | TIS | St | dreigen | to threaten | 3 | 21% |
| DID-S | TIS | St | droefheid | sadness | 3 | 21% |
| DID-S | TIS | St | falen | to fail | 3 | 21% |
| DID-S | NIS | St | gezwel | tumor | 3 | 21% |
| DID-S | TIS | St | instorten | to collapse | 3 | 21% |
| DID-S | TIS | St | knijpen | to pinch | 3 | 21% |
| DID-S | NIS | St | kotsen | to vomit | 3 | 21% |
| DID-S | NIS | St | kou | cold | 3 | 21% |
| DID-S | TIS | St | kuthoer | pussy whore | 3 | 21% |
| DID-S | TIS | St | lafaard | coward | 3 | 21% |
| DID-S | TIS | St | meester | master | 3 | 21% |
| DID-S | TIS | St | metaal | metal | 3 | 21% |
| DID-S | NIS | St | misleiden | to deceive | 3 | 21% |
| DID-S | NIS | St | moedeloos | despondent | 3 | 21% |
| DID-S | TIS | St | mond | mouth | 3 | 21% |
| DID-S | NIS | St | omkomen | to perish | 3 | 21% |
| DID-S | NIS | St | ongeluk | accident | 3 | 21% |
| DID-S | TIS | St | onzeker | uncertain | 3 | 21% |
| DID-S | NIS | St | schande | shame | 3 | 21% |
| DID-S | TIS | St | slet | slut | 3 | 21% |
| DID-S | TIS | St | snijden | to cut | 3 | 21% |
| DID-S | NIS | St | stank | stench | 3 | 21% |
| DID-S | TIS | St | stank | stench | 3 | 21% |
| DID-S | NIS | St | stikken | to suffocate | 3 | 21% |
| DID-S | NIS | St | tegenslag | setback | 3 | 21% |
| DID-S | NIS | St | tiran | tyrant | 3 | 21% |
| DID-S | TIS | St | treiteren | to harass | 3 | 21% |
| DID-S | TIS | St | uitkleden | to undress | 3 | 21% |
| DID-S | TIS | St | uitlachen | to laugh at | 3 | 21% |
| DID-S | NIS | St | verbranden | to burn | 3 | 21% |
| DID-S | TIS | St | verdriet | sadness | 3 | 21% |
| DID-S | NIS | St | vreemde | stranger | 3 | 21% |
| DID-S | NIS | St | walging | disgust | 3 | 21% |
| DID-S | NIS | St | wanhoop | despair | 3 | 21% |
| DID-S | TIS | St | wanhopen | to despair | 3 | 21% |
| DID-S | TIS | NSt | afgrijzen | horror | 3 | 21% |
| DID-S | TIS | NSt | afhakken | to chop off | 3 | 21% |
| DID-S | NIS | NSt | afkraken | to decry | 3 | 21% |
| DID-S | TIS | NSt | afsnauwen | to snap at | 3 | 21% |
| DID-S | TIS | NSt | angst | fear | 3 | 21% |
| DID-S | TIS | NSt | armoede | poverty | 3 | 21% |
| DID-S | NIS | NSt | belazerd | fooled | 3 | 21% |
| DID-S | NIS | NSt | bordeel | brothel | 3 | 21% |
| DID-S | TIS | NSt | bordeel | brothel | 3 | 21% |
| DID-S | NIS | NSt | chanteren | to blackmail | 3 | 21% |
| DID-S | TIS | NSt | depressie | depression | 3 | 21% |
| DID-S | TIS | NSt | diefstal | theft | 3 | 21% |
| DID-S | NIS | NSt | doodgaan | to die | 3 | 21% |
| DID-S | NIS | NSt | dwingen | to coerce or to force | 3 | 21% |
| DID-S | TIS | NSt | ellende | misery | 3 | 21% |
| DID-S | TIS | NSt | ergernis | annoyance | 3 | 21% |
| DID-S | TIS | NSt | etter | pus | 3 | 21% |
| DID-S | TIS | NSt | falen | to fail | 3 | 21% |
| DID-S | NIS | NSt | hysterie | hysteria | 3 | 21% |
| DID-S | TIS | NSt | hysterie | hysteria | 3 | 21% |
| DID-S | NIS | NSt | inbraak | burglary | 3 | 21% |
| DID-S | TIS | NSt | inbraak | burglary | 3 | 21% |
| DID-S | TIS | NSt | instorten | to collapse | 3 | 21% |
| DID-S | TIS | NSt | kelder | basement | 3 | 21% |
| DID-S | TIS | NSt | kogel | bullet | 3 | 21% |
| DID-S | NIS | NSt | krenken | to hurt | 3 | 21% |
| DID-S | NIS | NSt | kreunen | to moan | 3 | 21% |
| DID-S | NIS | NSt | kuthoer | pussy whore | 3 | 21% |
| DID-S | TIS | NSt | lafaard | coward | 3 | 21% |
| DID-S | TIS | NSt | man | man | 3 | 21% |
| DID-S | TIS | NSt | meeloper | opportunist | 3 | 21% |
| DID-S | NIS | NSt | misdaad | crime | 3 | 21% |
| DID-S | TIS | NSt | misdrijf | crime | 3 | 21% |
| DID-S | NIS | NSt | misleiden | to deceive | 3 | 21% |
| DID-S | TIS | NSt | moedeloos | despondent | 3 | 21% |
| DID-S | TIS | NSt | ontrouw | unfaithful | 3 | 21% |
| DID-S | NIS | NSt | opsluiten | to lock up | 3 | 21% |
| DID-S | NIS | NSt | slaaf | slave | 3 | 21% |
| DID-S | NIS | NSt | slijmen | suck up to | 3 | 21% |
| DID-S | NIS | NSt | trauma | trauma | 3 | 21% |
| DID-S | TIS | NSt | trauma | trauma | 3 | 21% |
| DID-S | TIS | NSt | verbranden | to burn | 3 | 21% |
| DID-S | NIS | NSt | verleidster | temptress | 3 | 21% |
| DID-S | NIS | NSt | verraad | betrayal | 3 | 21% |
| DID-S | TIS | NSt | wanhopen | to despair | 3 | 21% |
| DID-S | TIS | NSt | wreedheid | cruelty | 3 | 21% |
| DID-S | TIS | NSt | wurgen | to strangle | 3 | 21% |
| DID-S | NIS | NSt | zeuren | to nag | 3 | 21% |
| DID-S | NIS | NSt | zondebok | scapegoat | 3 | 21% |
| DID-S | TIS | NSn | absorptie | absorption | 3 | 21% |
| DID-S | TIS | NSn | armoede | poverty | 3 | 21% |
| DID-S | TIS | NSn | atoombom | nuclear bomb | 3 | 21% |
| DID-S | TIS | NSn | begraven | to bury | 3 | 21% |
| DID-S | TIS | NSn | beroerte | stroke | 3 | 21% |
| DID-S | NIS | NSn | bloot | naked | 3 | 21% |
| DID-S | NIS | NSn | dokter | (general practice) doctor | 3 | 21% |
| DID-S | NIS | NSn | dozijn | dozen | 3 | 21% |
| DID-S | TIS | NSn | element | element | 3 | 21% |
| DID-S | NIS | NSn | geit | goat | 3 | 21% |
| DID-S | TIS | NSn | huisdier | pet | 3 | 21% |
| DID-S | NIS | NSn | kader | framework | 3 | 21% |
| DID-S | NIS | NSn | kast | closet | 3 | 21% |
| DID-S | NIS | NSn | kogel | bullet | 3 | 21% |
| DID-S | TIS | NSn | kozijn | window frame | 3 | 21% |
| DID-S | NIS | NSn | krabben | to scratch | 3 | 21% |
| DID-S | TIS | NSn | krabben | to scratch | 3 | 21% |
| DID-S | NIS | NSn | mes | knife | 3 | 21% |
| DID-S | NIS | NSn | octaaf | octave | 3 | 21% |
| DID-S | TIS | NSn | ongeval | accident | 3 | 21% |
| DID-S | TIS | NSn | pasen | Easter | 3 | 21% |
| DID-S | NIS | NSn | razernij | fury | 3 | 21% |
| DID-S | TIS | NSn | register | register | 3 | 21% |
| DID-S | TIS | NSn | schuren | to polish | 3 | 21% |
| DID-S | NIS | NSn | snijden | to cut | 3 | 21% |
| DID-S | NIS | NSn | steil | steep | 3 | 21% |
| DID-S | TIS | NSn | stoelpoot | chair leg | 3 | 21% |
| DID-S | TIS | NSn | versie | version | 3 | 21% |
| DID-S | TIS | NSn | vreemde | stranger | 3 | 21% |
| DID-S | NIS | NSn | vuur | fire | 3 | 21% |
| DID-S | NIS | St | achterlaten | leave behind | 2 | 14% |
| DID-S | TIS | St | afgunst | envy | 2 | 14% |
| DID-S | NIS | St | afschuw | revulsion | 2 | 14% |
| DID-S | NIS | St | agressie | aggression | 2 | 14% |
| DID-S | TIS | St | agressie | aggression | 2 | 14% |
| DID-S | NIS | St | atoombom | nuclear bomb | 2 | 14% |
| DID-S | NIS | St | bedreigen | to threaten | 2 | 14% |
| DID-S | NIS | St | begraven | to bury | 2 | 14% |
| DID-S | NIS | St | beklemmen | to oppress | 2 | 14% |
| DID-S | TIS | St | belazerd | fooled | 2 | 14% |
| DID-S | NIS | St | beroerte | stroke | 2 | 14% |
| DID-S | NIS | St | besmetten | to contaminate | 2 | 14% |
| DID-S | TIS | St | blinddoek | blindfold | 2 | 14% |
| DID-S | TIS | St | bloedbad | bloodbath | 2 | 14% |
| DID-S | NIS | St | boos | angry | 2 | 14% |
| DID-S | TIS | St | boos | angry | 2 | 14% |
| DID-S | NIS | St | branden | to burn | 2 | 14% |
| DID-S | TIS | St | buurman | neighbor | 2 | 14% |
| DID-S | NIS | St | chanteren | to blackmail | 2 | 14% |
| DID-S | NIS | St | doodgaan | to die | 2 | 14% |
| DID-S | NIS | St | dubbel | double | 2 | 14% |
| DID-S | TIS | St | ergeren | to annoy | 2 | 14% |
| DID-S | NIS | St | getreiter | harassment | 2 | 14% |
| DID-S | TIS | St | getreiter | harassment | 2 | 14% |
| DID-S | NIS | St | geweld | violence | 2 | 14% |
| DID-S | NIS | St | hoer | whore | 2 | 14% |
| DID-S | TIS | St | hoer | whore | 2 | 14% |
| DID-S | NIS | St | kanker | cancer | 2 | 14% |
| DID-S | NIS | St | kelder | basement | 2 | 14% |
| DID-S | NIS | St | kreng | bitch | 2 | 14% |
| DID-S | NIS | St | krenken | to hurt | 2 | 14% |
| DID-S | TIS | St | liegen | to lie | 2 | 14% |
| DID-S | NIS | St | masker | mask | 2 | 14% |
| DID-S | TIS | St | misdrijf | crime | 2 | 14% |
| DID-S | TIS | St | moeder | mother | 2 | 14% |
| DID-S | NIS | St | ontslag | resignation or dismissal | 2 | 14% |
| DID-S | TIS | St | orgie | orgy | 2 | 14% |
| DID-S | NIS | St | pedofiel | paedophile | 2 | 14% |
| DID-S | TIS | St | piekeren | to mull | 2 | 14% |
| DID-S | TIS | St | plafond | ceiling | 2 | 14% |
| DID-S | NIS | St | regel | rule | 2 | 14% |
| DID-S | TIS | St | regel | rule | 2 | 14% |
| DID-S | TIS | St | ruzie | fight or quarrel | 2 | 14% |
| DID-S | TIS | St | sadist | sadist | 2 | 14% |
| DID-S | NIS | St | schaden | to damage | 2 | 14% |
| DID-S | NIS | St | schoft | bastard | 2 | 14% |
| DID-S | TIS | St | schoft | bastard | 2 | 14% |
| DID-S | NIS | St | schoppen | to kick | 2 | 14% |
| DID-S | NIS | St | slaaf | slave | 2 | 14% |
| DID-S | NIS | St | slecht | bad | 2 | 14% |
| DID-S | NIS | St | slet | slut | 2 | 14% |
| DID-S | TIS | St | steekwond | stab wound | 2 | 14% |
| DID-S | NIS | St | stinken | to stink | 2 | 14% |
| DID-S | TIS | St | stinken | to stink | 2 | 14% |
| DID-S | NIS | St | treiteren | to harass | 2 | 14% |
| DID-S | NIS | St | uitjouwen | to boo | 2 | 14% |
| DID-S | TIS | St | uitjouwen | to boo | 2 | 14% |
| DID-S | NIS | St | uitlachen | to laugh at | 2 | 14% |
| DID-S | NIS | St | vastbinden | to tie | 2 | 14% |
| DID-S | NIS | St | verlammen | to paralyze | 2 | 14% |
| DID-S | NIS | St | vernielen | to vandalize | 2 | 14% |
| DID-S | NIS | St | wanhopen | to despair | 2 | 14% |
| DID-S | TIS | St | wraak | revenge | 2 | 14% |
| DID-S | NIS | St | wreedheid | cruelty | 2 | 14% |
| DID-S | NIS | St | wurgen | to strangle | 2 | 14% |
| DID-S | NIS | St | zeer | ache | 2 | 14% |
| DID-S | TIS | St | zeuren | to nag | 2 | 14% |
| DID-S | NIS | NSt | achterlaten | leave behind | 2 | 14% |
| DID-S | TIS | NSt | afkraken | to decry | 2 | 14% |
| DID-S | NIS | NSt | afsnauwen | to snap at | 2 | 14% |
| DID-S | TIS | NSt | agressie | aggression | 2 | 14% |
| DID-S | NIS | NSt | angst | fear | 2 | 14% |
| DID-S | TIS | NSt | baby | baby | 2 | 14% |
| DID-S | TIS | NSt | bang | afraid | 2 | 14% |
| DID-S | NIS | NSt | beklemmen | to oppress | 2 | 14% |
| DID-S | TIS | NSt | belazerd | fooled | 2 | 14% |
| DID-S | TIS | NSt | boos | angry | 2 | 14% |
| DID-S | TIS | NSt | buurman | neighbor | 2 | 14% |
| DID-S | NIS | NSt | conflict | conflict | 2 | 14% |
| DID-S | TIS | NSt | consulaat | consulate | 2 | 14% |
| DID-S | NIS | NSt | diefstal | theft | 2 | 14% |
| DID-S | TIS | NSt | dokter | (general practice) doctor | 2 | 14% |
| DID-S | NIS | NSt | domkop | idiot | 2 | 14% |
| DID-S | TIS | NSt | droefheid | sadness | 2 | 14% |
| DID-S | TIS | NSt | droevig | sad | 2 | 14% |
| DID-S | TIS | NSt | dwerg | dwarf | 2 | 14% |
| DID-S | TIS | NSt | ergeren | to annoy | 2 | 14% |
| DID-S | NIS | NSt | ergernis | annoyance | 2 | 14% |
| DID-S | NIS | NSt | falen | to fail | 2 | 14% |
| DID-S | NIS | NSt | getreiter | harassment | 2 | 14% |
| DID-S | TIS | NSt | haak | hook | 2 | 14% |
| DID-S | TIS | NSt | hertogin | duchess | 2 | 14% |
| DID-S | TIS | NSt | hoer | whore | 2 | 14% |
| DID-S | TIS | NSt | ijzer | iron | 2 | 14% |
| DID-S | TIS | NSt | knijpen | to pinch | 2 | 14% |
| DID-S | NIS | NSt | krabben | to scratch | 2 | 14% |
| DID-S | TIS | NSt | kreunen | to moan | 2 | 14% |
| DID-S | TIS | NSt | kuthoer | pussy whore | 2 | 14% |
| DID-S | TIS | NSt | kwetsen | to hurt | 2 | 14% |
| DID-S | TIS | NSt | legpuzzel | jigsaw puzzle | 2 | 14% |
| DID-S | TIS | NSt | liegen | to lie | 2 | 14% |
| DID-S | TIS | NSt | masker | mask | 2 | 14% |
| DID-S | NIS | NSt | misvormen | to deform | 2 | 14% |
| DID-S | TIS | NSt | omroep | broadcasting | 2 | 14% |
| DID-S | NIS | NSt | ongeluk | accident | 2 | 14% |
| DID-S | TIS | NSt | ongeluk | accident | 2 | 14% |
| DID-S | NIS | NSt | ongewenst | unwanted | 2 | 14% |
| DID-S | NIS | NSt | onmacht | powerlessness | 2 | 14% |
| DID-S | TIS | NSt | onmacht | powerlessness | 2 | 14% |
| DID-S | NIS | NSt | ontrouw | unfaithful | 2 | 14% |
| DID-S | TIS | NSt | onzeker | uncertain | 2 | 14% |
| DID-S | TIS | NSt | opsluiten | to lock up | 2 | 14% |
| DID-S | TIS | NSt | pasen | Easter | 2 | 14% |
| DID-S | NIS | NSt | razernij | fury | 2 | 14% |
| DID-S | TIS | NSt | schoft | bastard | 2 | 14% |
| DID-S | TIS | NSt | schuren | to polish | 2 | 14% |
| DID-S | NIS | NSt | seks | sex | 2 | 14% |
| DID-S | TIS | NSt | seks | sex | 2 | 14% |
| DID-S | TIS | NSt | slaaf | slave | 2 | 14% |
| DID-S | NIS | NSt | snauwen | to snarl | 2 | 14% |
| DID-S | NIS | NSt | snijden | to cut | 2 | 14% |
| DID-S | TIS | NSt | stank | stench | 2 | 14% |
| DID-S | TIS | NSt | stellen | to set | 2 | 14% |
| DID-S | TIS | NSt | sterven | to die | 2 | 14% |
| DID-S | TIS | NSt | tegenslag | setback | 2 | 14% |
| DID-S | TIS | NSt | tiran | tyrant | 2 | 14% |
| DID-S | TIS | NSt | touw | rope | 2 | 14% |
| DID-S | TIS | NSt | treiteren | to harass | 2 | 14% |
| DID-S | TIS | NSt | uitjouwen | to boo | 2 | 14% |
| DID-S | TIS | NSt | uitkleden | to undress | 2 | 14% |
| DID-S | NIS | NSt | uitlachen | to laugh at | 2 | 14% |
| DID-S | NIS | NSt | verdord | withered | 2 | 14% |
| DID-S | TIS | NSt | verdriet | sadness | 2 | 14% |
| DID-S | TIS | NSt | verkrachting | rape | 2 | 14% |
| DID-S | NIS | NSt | wanhopen | to despair | 2 | 14% |
| DID-S | TIS | NSt | zuigen | to suck | 2 | 14% |
| DID-S | NIS | NSn | aanslag | attack | 2 | 14% |
| DID-S | NIS | NSn | beklemmen | to oppress | 2 | 14% |
| DID-S | NIS | NSn | bladzijde | page | 2 | 14% |
| DID-S | TIS | NSn | blikje | can | 2 | 14% |
| DID-S | TIS | NSn | bloot | naked | 2 | 14% |
| DID-S | NIS | NSn | broer | brother | 2 | 14% |
| DID-S | NIS | NSn | chanteren | to blackmail | 2 | 14% |
| DID-S | NIS | NSn | chauffeur | driver | 2 | 14% |
| DID-S | TIS | NSn | document | document | 2 | 14% |
| DID-S | TIS | NSn | dokter | (general practice) doctor | 2 | 14% |
| DID-S | NIS | NSn | doorslikken | to swallow | 2 | 14% |
| DID-S | NIS | NSn | embryo | embryo | 2 | 14% |
| DID-S | TIS | NSn | embryo | embryo | 2 | 14% |
| DID-S | TIS | NSn | geit | goat | 2 | 14% |
| DID-S | TIS | NSn | gijzeling | kidnapping | 2 | 14% |
| DID-S | TIS | NSn | hertogin | duchess | 2 | 14% |
| DID-S | NIS | NSn | hijgen | to pant | 2 | 14% |
| DID-S | NIS | NSn | huisdier | pet | 2 | 14% |
| DID-S | TIS | NSn | ijzer | iron | 2 | 14% |
| DID-S | NIS | NSn | ivoor | ivory | 2 | 14% |
| DID-S | TIS | NSn | juni | June | 2 | 14% |
| DID-S | NIS | NSn | knijpen | to pinch | 2 | 14% |
| DID-S | TIS | NSn | kogel | bullet | 2 | 14% |
| DID-S | TIS | NSn | kou | cold | 2 | 14% |
| DID-S | NIS | NSn | laden | to load | 2 | 14% |
| DID-S | TIS | NSn | laden | to load | 2 | 14% |
| DID-S | TIS | NSn | lawaai | noise | 2 | 14% |
| DID-S | NIS | NSn | legpuzzel | jigsaw puzzle | 2 | 14% |
| DID-S | NIS | NSn | likken | to lick | 2 | 14% |
| DID-S | TIS | NSn | likken | to lick | 2 | 14% |
| DID-S | NIS | NSn | magazijn | warehouse | 2 | 14% |
| DID-S | TIS | NSn | meester | master | 2 | 14% |
| DID-S | NIS | NSn | metaal | metal | 2 | 14% |
| DID-S | NIS | NSn | misvormen | to deform | 2 | 14% |
| DID-S | TIS | NSn | mond | mouth | 2 | 14% |
| DID-S | TIS | NSn | moord | murder | 2 | 14% |
| DID-S | NIS | NSn | ogen | eyes | 2 | 14% |
| DID-S | TIS | NSn | ontrouw | unfaithful | 2 | 14% |
| DID-S | TIS | NSn | oom | uncle | 2 | 14% |
| DID-S | NIS | NSn | pasen | Easter | 2 | 14% |
| DID-S | NIS | NSn | piemel | willy | 2 | 14% |
| DID-S | NIS | NSn | schaden | to damage | 2 | 14% |
| DID-S | TIS | NSn | slijmen | suck up to | 2 | 14% |
| DID-S | NIS | NSn | spoelen | to flush or to rinse | 2 | 14% |
| DID-S | TIS | NSn | stank | stench | 2 | 14% |
| DID-S | NIS | NSn | stellen | to set | 2 | 14% |
| DID-S | TIS | NSn | stellen | to set | 2 | 14% |
| DID-S | TIS | NSn | sterven | to die | 2 | 14% |
| DID-S | NIS | NSn | stoel | chair | 2 | 14% |
| DID-S | TIS | NSn | stoel | chair | 2 | 14% |
| DID-S | TIS | NSn | uitlachen | to laugh at | 2 | 14% |
| DID-S | NIS | NSn | verbranden | to burn | 2 | 14% |
| DID-S | TIS | NSn | verdord | withered | 2 | 14% |
| DID-S | NIS | NSn | vergroten | enlarge | 2 | 14% |
| DID-S | NIS | NSn | zuigen | to suck | 2 | 14% |
| DID-S | NIS | St | aanslag | attack | 1 | 7% |
| DID-S | TIS | St | aanslag | attack | 1 | 7% |
| DID-S | NIS | St | afblaffen | to bark at | 1 | 7% |
| DID-S | TIS | St | afkraken | to decry | 1 | 7% |
| DID-S | TIS | St | armoede | poverty | 1 | 7% |
| DID-S | TIS | St | baby | baby | 1 | 7% |
| DID-S | TIS | St | bad | bath | 1 | 7% |
| DID-S | TIS | St | bedrog | deceit | 1 | 7% |
| DID-S | NIS | St | bladzijde | page | 1 | 7% |
| DID-S | NIS | St | bloedbad | bloodbath | 1 | 7% |
| DID-S | NIS | St | bloot | naked | 1 | 7% |
| DID-S | NIS | St | bordeel | brothel | 1 | 7% |
| DID-S | NIS | St | buurman | neighbor | 1 | 7% |
| DID-S | TIS | St | cirkel | circle | 1 | 7% |
| DID-S | NIS | St | crimineel | criminal | 1 | 7% |
| DID-S | TIS | St | dakgoot | gutter | 1 | 7% |
| DID-S | NIS | St | depressie | depression | 1 | 7% |
| DID-S | NIS | St | diefstal | theft | 1 | 7% |
| DID-S | NIS | St | doden | to kill | 1 | 7% |
| DID-S | TIS | St | dokter | (general practice) doctor | 1 | 7% |
| DID-S | TIS | St | doodslag | manslaughter | 1 | 7% |
| DID-S | NIS | St | doodsteek | deathblow | 1 | 7% |
| DID-S | NIS | St | doorslikken | to swallow | 1 | 7% |
| DID-S | TIS | St | droevig | sad | 1 | 7% |
| DID-S | TIS | St | dubbel | double | 1 | 7% |
| DID-S | NIS | St | ellende | misery | 1 | 7% |
| DID-S | TIS | St | embryo | embryo | 1 | 7% |
| DID-S | NIS | St | etiket | label | 1 | 7% |
| DID-S | NIS | St | etter | pus | 1 | 7% |
| DID-S | TIS | St | gaatje | little hole | 1 | 7% |
| DID-S | TIS | St | gezwel | tumor | 1 | 7% |
| DID-S | TIS | St | gijzeling | kidnapping | 1 | 7% |
| DID-S | TIS | St | handen | hands | 1 | 7% |
| DID-S | NIS | St | haten | to hate | 1 | 7% |
| DID-S | TIS | St | hysterie | hysteria | 1 | 7% |
| DID-S | NIS | St | instorten | to collapse | 1 | 7% |
| DID-S | TIS | St | kader | framework | 1 | 7% |
| DID-S | TIS | St | kelder | basement | 1 | 7% |
| DID-S | NIS | St | knijpen | to pinch | 1 | 7% |
| DID-S | TIS | St | kou | cold | 1 | 7% |
| DID-S | NIS | St | krabben | to scratch | 1 | 7% |
| DID-S | NIS | St | kreunen | to moan | 1 | 7% |
| DID-S | NIS | St | lijden | to suffer | 1 | 7% |
| DID-S | NIS | St | man | man | 1 | 7% |
| DID-S | NIS | St | martelen | to torture | 1 | 7% |
| DID-S | NIS | St | meeloper | opportunist | 1 | 7% |
| DID-S | TIS | St | mes | knife | 1 | 7% |
| DID-S | NIS | St | messteek | knife stab | 1 | 7% |
| DID-S | TIS | St | middel | middle | 1 | 7% |
| DID-S | NIS | St | misdaad | crime | 1 | 7% |
| DID-S | TIS | St | misdaad | crime | 1 | 7% |
| DID-S | NIS | St | miskraam | miscarriage | 1 | 7% |
| DID-S | TIS | St | miskraam | miscarriage | 1 | 7% |
| DID-S | TIS | St | misleiden | to deceive | 1 | 7% |
| DID-S | TIS | St | misvormen | to deform | 1 | 7% |
| DID-S | NIS | St | moeder | mother | 1 | 7% |
| DID-S | NIS | St | mond | mouth | 1 | 7% |
| DID-S | NIS | St | moord | murder | 1 | 7% |
| DID-S | NIS | St | noodkreet | cry for help | 1 | 7% |
| DID-S | NIS | St | ongeval | accident | 1 | 7% |
| DID-S | TIS | St | ontrouw | unfaithful | 1 | 7% |
| DID-S | NIS | St | oom | uncle | 1 | 7% |
| DID-S | TIS | St | oom | uncle | 1 | 7% |
| DID-S | NIS | St | oorlog | war | 1 | 7% |
| DID-S | NIS | St | oplichten | to scam | 1 | 7% |
| DID-S | NIS | St | opsluiten | to lock up | 1 | 7% |
| DID-S | NIS | St | piemel | willy | 1 | 7% |
| DID-S | NIS | St | plank | shelf | 1 | 7% |
| DID-S | TIS | St | razernij | fury | 1 | 7% |
| DID-S | NIS | St | sadist | sadist | 1 | 7% |
| DID-S | TIS | St | schaden | to damage | 1 | 7% |
| DID-S | NIS | St | slijmen | suck up to | 1 | 7% |
| DID-S | NIS | St | spoelen | to flush or to rinse | 1 | 7% |
| DID-S | NIS | St | steil | steep | 1 | 7% |
| DID-S | NIS | St | sterven | to die | 1 | 7% |
| DID-S | NIS | St | stiekem | secretly | 1 | 7% |
| DID-S | NIS | St | stoep | sidewalk | 1 | 7% |
| DID-S | TIS | St | tegenslag | setback | 1 | 7% |
| DID-S | TIS | St | touw | rope | 1 | 7% |
| DID-S | TIS | St | treden | to step | 1 | 7% |
| DID-S | NIS | St | trottoir | pavement | 1 | 7% |
| DID-S | NIS | St | uitkleden | to undress | 1 | 7% |
| DID-S | TIS | St | vastbinden | to tie | 1 | 7% |
| DID-S | NIS | St | verdrinken | to drown | 1 | 7% |
| DID-S | TIS | St | verdrinken | to drown | 1 | 7% |
| DID-S | NIS | St | verleidster | temptress | 1 | 7% |
| DID-S | TIS | St | vernielen | to vandalize | 1 | 7% |
| DID-S | NIS | St | versie | version | 1 | 7% |
| DID-S | TIS | St | versie | version | 1 | 7% |
| DID-S | NIS | St | verzuipen | to drown | 1 | 7% |
| DID-S | TIS | St | verzuipen | to drown | 1 | 7% |
| DID-S | TIS | St | vreemde | stranger | 1 | 7% |
| DID-S | NIS | St | vuur | fire | 1 | 7% |
| DID-S | TIS | St | vuur | fire | 1 | 7% |
| DID-S | NIS | St | woest | enraged | 1 | 7% |
| DID-S | TIS | St | woest | enraged | 1 | 7% |
| DID-S | TIS | St | zakje | little bag | 1 | 7% |
| DID-S | NIS | St | zondebok | scapegoat | 1 | 7% |
| DID-S | TIS | St | zondebok | scapegoat | 1 | 7% |
| DID-S | TIS | NSt | achterlaten | leave behind | 1 | 7% |
| DID-S | TIS | NSt | afblaffen | to bark at | 1 | 7% |
| DID-S | NIS | NSt | afgunst | envy | 1 | 7% |
| DID-S | TIS | NSt | afgunst | envy | 1 | 7% |
| DID-S | TIS | NSt | afkeer | aversion | 1 | 7% |
| DID-S | NIS | NSt | afschuw | revulsion | 1 | 7% |
| DID-S | NIS | NSt | afwijzing | rejection | 1 | 7% |
| DID-S | TIS | NSt | afwijzing | rejection | 1 | 7% |
| DID-S | NIS | NSt | baby | baby | 1 | 7% |
| DID-S | TIS | NSt | bad | bath | 1 | 7% |
| DID-S | NIS | NSt | bang | afraid | 1 | 7% |
| DID-S | TIS | NSt | bedreigen | to threaten | 1 | 7% |
| DID-S | TIS | NSt | bedrieger | deceiver | 1 | 7% |
| DID-S | TIS | NSt | begraven | to bury | 1 | 7% |
| DID-S | TIS | NSt | benauwen | to agitate | 1 | 7% |
| DID-S | NIS | NSt | blinddoek | blindfold | 1 | 7% |
| DID-S | TIS | NSt | branden | to burn | 1 | 7% |
| DID-S | TIS | NSt | chauffeur | driver | 1 | 7% |
| DID-S | TIS | NSt | cirkel | circle | 1 | 7% |
| DID-S | TIS | NSt | conflict | conflict | 1 | 7% |
| DID-S | NIS | NSt | consulaat | consulate | 1 | 7% |
| DID-S | NIS | NSt | depressie | depression | 1 | 7% |
| DID-S | TIS | NSt | domkop | idiot | 1 | 7% |
| DID-S | NIS | NSt | dreigen | to threaten | 1 | 7% |
| DID-S | TIS | NSt | dreigen | to threaten | 1 | 7% |
| DID-S | NIS | NSt | droefheid | sadness | 1 | 7% |
| DID-S | TIS | NSt | dwingen | to coerce or to force | 1 | 7% |
| DID-S | TIS | NSt | elleboog | elbow | 1 | 7% |
| DID-S | TIS | NSt | embryo | embryo | 1 | 7% |
| DID-S | NIS | NSt | ergeren | to annoy | 1 | 7% |
| DID-S | TIS | NSt | haken | hooks | 1 | 7% |
| DID-S | TIS | NSt | haten | to hate | 1 | 7% |
| DID-S | NIS | NSt | ijzer | iron | 1 | 7% |
| DID-S | NIS | NSt | ivoor | ivory | 1 | 7% |
| DID-S | TIS | NSt | kader | framework | 1 | 7% |
| DID-S | NIS | NSt | kerk | church | 1 | 7% |
| DID-S | TIS | NSt | kerk | church | 1 | 7% |
| DID-S | NIS | NSt | kou | cold | 1 | 7% |
| DID-S | TIS | NSt | kou | cold | 1 | 7% |
| DID-S | NIS | NSt | kwetsen | to hurt | 1 | 7% |
| DID-S | NIS | NSt | laden | to load | 1 | 7% |
| DID-S | NIS | NSt | liegen | to lie | 1 | 7% |
| DID-S | TIS | NSt | lijden | to suffer | 1 | 7% |
| DID-S | TIS | NSt | likken | to lick | 1 | 7% |
| DID-S | NIS | NSt | meester | master | 1 | 7% |
| DID-S | TIS | NSt | meester | master | 1 | 7% |
| DID-S | TIS | NSt | mes | knife | 1 | 7% |
| DID-S | TIS | NSt | metaal | metal | 1 | 7% |
| DID-S | TIS | NSt | misleiden | to deceive | 1 | 7% |
| DID-S | TIS | NSt | mismaakt | deformed | 1 | 7% |
| DID-S | TIS | NSt | misvormen | to deform | 1 | 7% |
| DID-S | TIS | NSt | noodkreet | cry for help | 1 | 7% |
| DID-S | TIS | NSt | octaaf | octave | 1 | 7% |
| DID-S | TIS | NSt | ogen | eyes | 1 | 7% |
| DID-S | TIS | NSt | ongeval | accident | 1 | 7% |
| DID-S | TIS | NSt | ongewenst | unwanted | 1 | 7% |
| DID-S | TIS | NSt | ontslag | resignation or dismissal | 1 | 7% |
| DID-S | NIS | NSt | onzeker | uncertain | 1 | 7% |
| DID-S | TIS | NSt | oom | uncle | 1 | 7% |
| DID-S | TIS | NSt | piekeren | to mull | 1 | 7% |
| DID-S | TIS | NSt | piemel | willy | 1 | 7% |
| DID-S | NIS | NSt | pijn | pain | 1 | 7% |
| DID-S | TIS | NSt | pijn | pain | 1 | 7% |
| DID-S | TIS | NSt | pilaar | pillar | 1 | 7% |
| DID-S | TIS | NSt | razernij | fury | 1 | 7% |
| DID-S | TIS | NSt | register | register | 1 | 7% |
| DID-S | TIS | NSt | ruzie | fight or quarrel | 1 | 7% |
| DID-S | NIS | NSt | schaden | to damage | 1 | 7% |
| DID-S | TIS | NSt | schaden | to damage | 1 | 7% |
| DID-S | NIS | NSt | schande | shame | 1 | 7% |
| DID-S | TIS | NSt | schande | shame | 1 | 7% |
| DID-S | TIS | NSt | schok | shock | 1 | 7% |
| DID-S | NIS | NSt | schoppen | to kick | 1 | 7% |
| DID-S | TIS | NSt | schroef | screw | 1 | 7% |
| DID-S | TIS | NSt | snauwen | to snarl | 1 | 7% |
| DID-S | TIS | NSt | snijden | to cut | 1 | 7% |
| DID-S | TIS | NSt | spoelen | to flush or to rinse | 1 | 7% |
| DID-S | TIS | NSt | steil | steep | 1 | 7% |
| DID-S | NIS | NSt | stellen | to set | 1 | 7% |
| DID-S | TIS | NSt | stiekem | secretly | 1 | 7% |
| DID-S | TIS | NSt | stikken | to suffocate | 1 | 7% |
| DID-S | TIS | NSt | stomerij | drycleaner | 1 | 7% |
| DID-S | TIS | NSt | tegel | tile | 1 | 7% |
| DID-S | TIS | NSt | traject | route | 1 | 7% |
| DID-S | NIS | NSt | trappen | to kick | 1 | 7% |
| DID-S | NIS | NSt | uitgescholden | scolded | 1 | 7% |
| DID-S | TIS | NSt | uitlachen | to laugh at | 1 | 7% |
| DID-S | TIS | NSt | vader | father | 1 | 7% |
| DID-S | TIS | NSt | vastbinden | to tie | 1 | 7% |
| DID-S | NIS | NSt | verdriet | sadness | 1 | 7% |
| DID-S | TIS | NSt | vergroten | enlarge | 1 | 7% |
| DID-S | TIS | NSt | verraad | betrayal | 1 | 7% |
| DID-S | TIS | NSt | vies | dirty | 1 | 7% |
| DID-S | TIS | NSt | vreemde | stranger | 1 | 7% |
| DID-S | TIS | NSt | walging | disgust | 1 | 7% |
| DID-S | NIS | NSt | woest | enraged | 1 | 7% |
| DID-S | TIS | NSt | woest | enraged | 1 | 7% |
| DID-S | TIS | NSt | wraak | revenge | 1 | 7% |
| DID-S | TIS | NSt | zakje | little bag | 1 | 7% |
| DID-S | NIS | NSt | zuigen | to suck | 1 | 7% |
| DID-S | TIS | NSn | aanslag | attack | 1 | 7% |
| DID-S | NIS | NSn | achterlaten | leave behind | 1 | 7% |
| DID-S | TIS | NSn | afblaffen | to bark at | 1 | 7% |
| DID-S | TIS | NSn | afgrijzen | horror | 1 | 7% |
| DID-S | TIS | NSn | afhakken | to chop off | 1 | 7% |
| DID-S | TIS | NSn | afkeer | aversion | 1 | 7% |
| DID-S | NIS | NSn | afscheid | goodbye | 1 | 7% |
| DID-S | TIS | NSn | afscheid | goodbye | 1 | 7% |
| DID-S | NIS | NSn | afschuw | revulsion | 1 | 7% |
| DID-S | TIS | NSn | afschuw | revulsion | 1 | 7% |
| DID-S | NIS | NSn | alleen | alone | 1 | 7% |
| DID-S | NIS | NSn | armoede | poverty | 1 | 7% |
| DID-S | TIS | NSn | baby | baby | 1 | 7% |
| DID-S | TIS | NSn | bad | bath | 1 | 7% |
| DID-S | TIS | NSn | bed | bed | 1 | 7% |
| DID-S | NIS | NSn | bedrog | deceit | 1 | 7% |
| DID-S | NIS | NSn | begraven | to bury | 1 | 7% |
| DID-S | TIS | NSn | beklemmen | to oppress | 1 | 7% |
| DID-S | NIS | NSn | belazerd | fooled | 1 | 7% |
| DID-S | TIS | NSn | belazerd | fooled | 1 | 7% |
| DID-S | NIS | NSn | benauwen | to agitate | 1 | 7% |
| DID-S | NIS | NSn | beroerte | stroke | 1 | 7% |
| DID-S | TIS | NSn | bordeel | brothel | 1 | 7% |
| DID-S | NIS | NSn | branden | to burn | 1 | 7% |
| DID-S | NIS | NSn | buurman | neighbor | 1 | 7% |
| DID-S | TIS | NSn | chanteren | to blackmail | 1 | 7% |
| DID-S | TIS | NSn | conflict | conflict | 1 | 7% |
| DID-S | TIS | NSn | crimineel | criminal | 1 | 7% |
| DID-S | TIS | NSn | diefstal | theft | 1 | 7% |
| DID-S | TIS | NSn | domkop | idiot | 1 | 7% |
| DID-S | NIS | NSn | doodgaan | to die | 1 | 7% |
| DID-S | TIS | NSn | doodgaan | to die | 1 | 7% |
| DID-S | TIS | NSn | doodslag | manslaughter | 1 | 7% |
| DID-S | NIS | NSn | droevig | sad | 1 | 7% |
| DID-S | NIS | NSn | dubbel | double | 1 | 7% |
| DID-S | TIS | NSn | dubbel | double | 1 | 7% |
| DID-S | TIS | NSn | elleboog | elbow | 1 | 7% |
| DID-S | NIS | NSn | etter | pus | 1 | 7% |
| DID-S | TIS | NSn | falen | to fail | 1 | 7% |
| DID-S | TIS | NSn | gezwel | tumor | 1 | 7% |
| DID-S | TIS | NSn | hagedis | lizard | 1 | 7% |
| DID-S | TIS | NSn | haken | hooks | 1 | 7% |
| DID-S | TIS | NSn | handen | hands | 1 | 7% |
| DID-S | TIS | NSn | hijgen | to pant | 1 | 7% |
| DID-S | NIS | NSn | hoer | whore | 1 | 7% |
| DID-S | TIS | NSn | hoer | whore | 1 | 7% |
| DID-S | NIS | NSn | hysterie | hysteria | 1 | 7% |
| DID-S | TIS | NSn | inbraak | burglary | 1 | 7% |
| DID-S | TIS | NSn | incest | incest | 1 | 7% |
| DID-S | NIS | NSn | instorten | to collapse | 1 | 7% |
| DID-S | TIS | NSn | kanker | cancer | 1 | 7% |
| DID-S | TIS | NSn | kelder | basement | 1 | 7% |
| DID-S | NIS | NSn | krenken | to hurt | 1 | 7% |
| DID-S | TIS | NSn | krenken | to hurt | 1 | 7% |
| DID-S | TIS | NSn | kwetsen | to hurt | 1 | 7% |
| DID-S | TIS | NSn | legpuzzel | jigsaw puzzle | 1 | 7% |
| DID-S | NIS | NSn | lepra | leprosy | 1 | 7% |
| DID-S | TIS | NSn | liegen | to lie | 1 | 7% |
| DID-S | NIS | NSn | man | man | 1 | 7% |
| DID-S | TIS | NSn | martelen | to torture | 1 | 7% |
| DID-S | NIS | NSn | masker | mask | 1 | 7% |
| DID-S | TIS | NSn | masker | mask | 1 | 7% |
| DID-S | TIS | NSn | meeloper | opportunist | 1 | 7% |
| DID-S | NIS | NSn | meester | master | 1 | 7% |
| DID-S | TIS | NSn | mes | knife | 1 | 7% |
| DID-S | TIS | NSn | middel | middle | 1 | 7% |
| DID-S | TIS | NSn | misdrijf | crime | 1 | 7% |
| DID-S | TIS | NSn | miskraam | miscarriage | 1 | 7% |
| DID-S | NIS | NSn | misleiden | to deceive | 1 | 7% |
| DID-S | TIS | NSn | misleiden | to deceive | 1 | 7% |
| DID-S | TIS | NSn | mislukt | failed | 1 | 7% |
| DID-S | TIS | NSn | mismaakt | deformed | 1 | 7% |
| DID-S | TIS | NSn | misvormen | to deform | 1 | 7% |
| DID-S | TIS | NSn | moedeloos | despondent | 1 | 7% |
| DID-S | NIS | NSn | moeder | mother | 1 | 7% |
| DID-S | NIS | NSn | mond | mouth | 1 | 7% |
| DID-S | NIS | NSn | nacht | night | 1 | 7% |
| DID-S | TIS | NSn | nacht | night | 1 | 7% |
| DID-S | TIS | NSn | octaaf | octave | 1 | 7% |
| DID-S | TIS | NSn | omkomen | to perish | 1 | 7% |
| DID-S | NIS | NSn | ongeval | accident | 1 | 7% |
| DID-S | TIS | NSn | onmacht | powerlessness | 1 | 7% |
| DID-S | NIS | NSn | ontslag | resignation or dismissal | 1 | 7% |
| DID-S | TIS | NSn | onzeker | uncertain | 1 | 7% |
| DID-S | NIS | NSn | oom | uncle | 1 | 7% |
| DID-S | TIS | NSn | oorlog | war | 1 | 7% |
| DID-S | NIS | NSn | oplichten | to scam | 1 | 7% |
| DID-S | TIS | NSn | oplichten | to scam | 1 | 7% |
| DID-S | TIS | NSn | orgie | orgy | 1 | 7% |
| DID-S | TIS | NSn | pedofiel | paedophile | 1 | 7% |
| DID-S | TIS | NSn | piekeren | to mull | 1 | 7% |
| DID-S | TIS | NSn | piemel | willy | 1 | 7% |
| DID-S | TIS | NSn | razernij | fury | 1 | 7% |
| DID-S | NIS | NSn | regel | rule | 1 | 7% |
| DID-S | TIS | NSn | roofmoord | robbery with murder | 1 | 7% |
| DID-S | TIS | NSn | schaden | to damage | 1 | 7% |
| DID-S | NIS | NSn | schoft | bastard | 1 | 7% |
| DID-S | NIS | NSn | schok | shock | 1 | 7% |
| DID-S | NIS | NSn | seks | sex | 1 | 7% |
| DID-S | NIS | NSn | slijmen | suck up to | 1 | 7% |
| DID-S | TIS | NSn | snijden | to cut | 1 | 7% |
| DID-S | NIS | NSn | tegenslag | setback | 1 | 7% |
| DID-S | NIS | NSn | trappen | to kick | 1 | 7% |
| DID-S | TIS | NSn | trappen | to kick | 1 | 7% |
| DID-S | TIS | NSn | trauma | trauma | 1 | 7% |
| DID-S | TIS | NSn | treiteren | to harass | 1 | 7% |
| DID-S | TIS | NSn | uitgescholden | scolded | 1 | 7% |
| DID-S | TIS | NSn | uitkleden | to undress | 1 | 7% |
| DID-S | NIS | NSn | uitlachen | to laugh at | 1 | 7% |
| DID-S | NIS | NSn | vastbinden | to tie | 1 | 7% |
| DID-S | TIS | NSn | verbranden | to burn | 1 | 7% |
| DID-S | TIS | NSn | verdrinken | to drown | 1 | 7% |
| DID-S | NIS | NSn | vies | dirty | 1 | 7% |
| DID-S | NIS | NSn | wanhopen | to despair | 1 | 7% |
| DID-S | TIS | NSn | woest | enraged | 1 | 7% |
| DID-S | NIS | NSn | zeer | ache | 1 | 7% |
| DID-S | TIS | NSn | zondebok | scapegoat | 1 | 7% |
| DID-S | TIS | NSn | zuigen | to suck | 1 | 7% |
| CTRL^^n3^ | NIS | NSt | incest | incest | 14 | 93% |
| CTRL | NIS | NSt | moord | murder | 14 | 93% |
| CTRL | NIS | NSt | wurgen | to strangle | 14 | 93% |
| CTRL | TIS | St | bang | afraid | 13 | 87% |
| CTRL | NIS | NSt | bloedbad | bloodbath | 13 | 87% |
| CTRL | NIS | NSt | doodslag | manslaughter | 13 | 87% |
| CTRL | NIS | NSt | folteren | to torture | 13 | 87% |
| CTRL | NIS | NSt | martelen | to torture | 13 | 87% |
| CTRL | NIS | NSt | misdrijf | crime | 13 | 87% |
| CTRL | NIS | NSt | oorlog | war | 13 | 87% |
| CTRL | NIS | NSt | stikken | to suffocate | 13 | 87% |
| CTRL | TIS | St | onrecht | injustice | 12 | 80% |
| CTRL | TIS | NSt | bloedbad | bloodbath | 12 | 80% |
| CTRL | NIS | NSt | crimineel | criminal | 12 | 80% |
| CTRL | NIS | NSt | doden | to kill | 12 | 80% |
| CTRL | NIS | NSt | verdrinken | to drown | 12 | 80% |
| CTRL | NIS | NSt | verkrachting | rape | 12 | 80% |
| CTRL | NIS | NSt | wreedheid | cruelty | 12 | 80% |
| CTRL | TIS | St | lijden | to suffer | 11 | 73% |
| CTRL | NIS | St | onzeker | uncertain | 11 | 73% |
| CTRL | NIS | NSt | doodsteek | deathblow | 11 | 73% |
| CTRL | TIS | NSt | oorlog | war | 11 | 73% |
| CTRL | NIS | NSt | roofmoord | robbery with murder | 11 | 73% |
| CTRL | TIS | NSt | roofmoord | robbery with murder | 11 | 73% |
| CTRL | TIS | NSn | regenton | rain barrel | 11 | 73% |
| CTRL | TIS | NSn | stukadoor | plasterer | 11 | 73% |
| CTRL | TIS | St | angst | fear | 10 | 67% |
| CTRL | TIS | NSt | doodslag | manslaughter | 10 | 67% |
| CTRL | NIS | NSt | geweld | violence | 10 | 67% |
| CTRL | NIS | NSt | gijzeling | kidnapping | 10 | 67% |
| CTRL | NIS | NSt | pedofiel | paedophile | 10 | 67% |
| CTRL | NIS | NSt | sadist | sadist | 10 | 67% |
| CTRL | TIS | NSt | steekwond | stab wound | 10 | 67% |
| CTRL | TIS | NSn | consulaat | consulate | 10 | 67% |
| CTRL | NIS | NSn | telegram | telegram | 10 | 67% |
| CTRL | NIS | St | ergeren | to annoy | 9 | 60% |
| CTRL | TIS | St | kwetsen | to hurt | 9 | 60% |
| CTRL | TIS | St | ongewenst | unwanted | 9 | 60% |
| CTRL | TIS | St | onmacht | powerlessness | 9 | 60% |
| CTRL | NIS | St | piekeren | to mull | 9 | 60% |
| CTRL | TIS | St | pijn | pain | 9 | 60% |
| CTRL | NIS | St | verdriet | sadness | 9 | 60% |
| CTRL | TIS | St | verdriet | sadness | 9 | 60% |
| CTRL | NIS | NSt | beroerte | stroke | 9 | 60% |
| CTRL | TIS | NSt | martelen | to torture | 9 | 60% |
| CTRL | NIS | NSt | messteek | knife stab | 9 | 60% |
| CTRL | TIS | NSt | moord | murder | 9 | 60% |
| CTRL | NIS | NSt | omkomen | to perish | 9 | 60% |
| CTRL | TIS | NSt | oplichten | to scam | 9 | 60% |
| CTRL | NIS | NSn | dakgoot | gutter | 9 | 60% |
| CTRL | NIS | NSn | haak | hook | 9 | 60% |
| CTRL | TIS | NSn | kenteken | license plate | 9 | 60% |
| CTRL | NIS | NSn | leuning | railing | 9 | 60% |
| CTRL | TIS | NSn | potlood | pencil | 9 | 60% |
| CTRL | TIS | NSn | stomerij | drycleaner | 9 | 60% |
| CTRL | NIS | NSn | stukadoor | plasterer | 9 | 60% |
| CTRL | TIS | NSn | tegel | tile | 9 | 60% |
| CTRL | NIS | NSn | tijdperk | era | 9 | 60% |
| CTRL | NIS | NSn | uitgever | publisher | 9 | 60% |
| CTRL | TIS | St | agressie | aggression | 8 | 53% |
| CTRL | TIS | St | benauwen | to agitate | 8 | 53% |
| CTRL | TIS | St | dwingen | to coerce or to force | 8 | 53% |
| CTRL | TIS | St | ellende | misery | 8 | 53% |
| CTRL | NIS | St | falen | to fail | 8 | 53% |
| CTRL | TIS | St | geweld | violence | 8 | 53% |
| CTRL | TIS | St | instorten | to collapse | 8 | 53% |
| CTRL | NIS | St | onmacht | powerlessness | 8 | 53% |
| CTRL | TIS | St | onzeker | uncertain | 8 | 53% |
| CTRL | TIS | St | ruzie | fight or quarrel | 8 | 53% |
| CTRL | NIS | St | tegenslag | setback | 8 | 53% |
| CTRL | TIS | St | trauma | trauma | 8 | 53% |
| CTRL | TIS | St | verkrachting | rape | 8 | 53% |
| CTRL | NIS | NSt | aanslag | attack | 8 | 53% |
| CTRL | NIS | NSt | atoombom | nuclear bomb | 8 | 53% |
| CTRL | TIS | NSt | atoombom | nuclear bomb | 8 | 53% |
| CTRL | TIS | NSt | beroerte | stroke | 8 | 53% |
| CTRL | TIS | NSt | doodsteek | deathblow | 8 | 53% |
| CTRL | TIS | NSt | haten | to hate | 8 | 53% |
| CTRL | NIS | NSt | misdaad | crime | 8 | 53% |
| CTRL | NIS | NSt | ontrouw | unfaithful | 8 | 53% |
| CTRL | NIS | NSt | slet | slut | 8 | 53% |
| CTRL | NIS | NSt | treiteren | to harass | 8 | 53% |
| CTRL | TIS | NSt | wurgen | to strangle | 8 | 53% |
| CTRL | NIS | NSn | bestek | cutlery | 8 | 53% |
| CTRL | NIS | NSn | flacon | bottle or vial | 8 | 53% |
| CTRL | TIS | NSn | grondstof | natural resource | 8 | 53% |
| CTRL | TIS | NSn | haak | hook | 8 | 53% |
| CTRL | NIS | NSn | ijzer | iron | 8 | 53% |
| CTRL | TIS | NSn | ijzer | iron | 8 | 53% |
| CTRL | TIS | NSn | leuning | railing | 8 | 53% |
| CTRL | NIS | NSn | metselaar | bricklayer | 8 | 53% |
| CTRL | TIS | NSn | metselaar | bricklayer | 8 | 53% |
| CTRL | NIS | NSn | plank | shelf | 8 | 53% |
| CTRL | NIS | NSn | register | register | 8 | 53% |
| CTRL | NIS | NSn | schuren | to polish | 8 | 53% |
| CTRL | TIS | NSn | stellen | to set | 8 | 53% |
| CTRL | TIS | NSn | telegram | telegram | 8 | 53% |
| CTRL | NIS | NSn | teller | counter | 8 | 53% |
| CTRL | NIS | NSn | uitkleden | to undress | 8 | 53% |
| CTRL | NIS | NSn | versie | version | 8 | 53% |
| CTRL | NIS | NSn | vierkant | square | 8 | 53% |
| CTRL | NIS | NSn | zakje | little bag | 8 | 53% |
| CTRL | TIS | NSn | zakje | little bag | 8 | 53% |
| CTRL | NIS | NSn | zandloper | hourglass | 8 | 53% |
| CTRL | TIS | NSn | zegel | seal | 8 | 53% |
| CTRL | TIS | St | afkraken | to decry | 7 | 47% |
| CTRL | TIS | St | bedreigen | to threaten | 7 | 47% |
| CTRL | TIS | St | beklemmen | to oppress | 7 | 47% |
| CTRL | TIS | St | dreigen | to threaten | 7 | 47% |
| CTRL | TIS | St | droefheid | sadness | 7 | 47% |
| CTRL | TIS | St | dwang | coercion or force | 7 | 47% |
| CTRL | NIS | St | ergernis | annoyance | 7 | 47% |
| CTRL | NIS | St | gezwel | tumor | 7 | 47% |
| CTRL | NIS | St | kou | cold | 7 | 47% |
| CTRL | TIS | St | misdrijf | crime | 7 | 47% |
| CTRL | TIS | St | schaden | to damage | 7 | 47% |
| CTRL | TIS | St | verraad | betrayal | 7 | 47% |
| CTRL | TIS | St | zeer | ache | 7 | 47% |
| CTRL | NIS | St | zeuren | to nag | 7 | 47% |
| CTRL | TIS | NSt | aanslag | attack | 7 | 47% |
| CTRL | NIS | NSt | afgrijzen | horror | 7 | 47% |
| CTRL | NIS | NSt | bedrieger | deceiver | 7 | 47% |
| CTRL | TIS | NSt | crimineel | criminal | 7 | 47% |
| CTRL | TIS | NSt | diefstal | theft | 7 | 47% |
| CTRL | TIS | NSt | doden | to kill | 7 | 47% |
| CTRL | NIS | NSt | ellende | misery | 7 | 47% |
| CTRL | TIS | NSt | folteren | to torture | 7 | 47% |
| CTRL | TIS | NSt | gijzeling | kidnapping | 7 | 47% |
| CTRL | NIS | NSt | haten | to hate | 7 | 47% |
| CTRL | TIS | NSt | kanker | cancer | 7 | 47% |
| CTRL | NIS | NSt | lepra | leprosy | 7 | 47% |
| CTRL | TIS | NSt | lepra | leprosy | 7 | 47% |
| CTRL | TIS | NSt | messteek | knife stab | 7 | 47% |
| CTRL | TIS | NSt | slet | slut | 7 | 47% |
| CTRL | NIS | NSt | tiran | tyrant | 7 | 47% |
| CTRL | NIS | NSt | verraad | betrayal | 7 | 47% |
| CTRL | TIS | NSt | verzuipen | to drown | 7 | 47% |
| CTRL | TIS | NSt | wraak | revenge | 7 | 47% |
| CTRL | TIS | NSn | bestek | cutlery | 7 | 47% |
| CTRL | NIS | NSn | consulaat | consulate | 7 | 47% |
| CTRL | NIS | NSn | element | element | 7 | 47% |
| CTRL | NIS | NSn | grondstof | natural resource | 7 | 47% |
| CTRL | TIS | NSn | ivoor | ivory | 7 | 47% |
| CTRL | NIS | NSn | omroep | broadcasting | 7 | 47% |
| CTRL | TIS | NSn | omroep | broadcasting | 7 | 47% |
| CTRL | TIS | NSn | postzegel | stamp | 7 | 47% |
| CTRL | TIS | NSn | roeren | to stir | 7 | 47% |
| CTRL | NIS | NSn | schroef | screw | 7 | 47% |
| CTRL | NIS | NSn | stomerij | drycleaner | 7 | 47% |
| CTRL | NIS | NSn | tegel | tile | 7 | 47% |
| CTRL | NIS | NSn | touw | rope | 7 | 47% |
| CTRL | NIS | NSn | traject | route | 7 | 47% |
| CTRL | TIS | NSn | versie | version | 7 | 47% |
| CTRL | TIS | St | afscheid | goodbye | 6 | 40% |
| CTRL | NIS | St | afwijzing | rejection | 6 | 40% |
| CTRL | TIS | St | belazerd | fooled | 6 | 40% |
| CTRL | NIS | St | kanker | cancer | 6 | 40% |
| CTRL | TIS | St | omkomen | to perish | 6 | 40% |
| CTRL | TIS | St | piekeren | to mull | 6 | 40% |
| CTRL | TIS | St | schuldig | guilty | 6 | 40% |
| CTRL | TIS | St | wanhopen | to despair | 6 | 40% |
| CTRL | NIS | NSt | chanteren | to blackmail | 6 | 40% |
| CTRL | TIS | NSt | chanteren | to blackmail | 6 | 40% |
| CTRL | TIS | NSt | hysterie | hysteria | 6 | 40% |
| CTRL | TIS | NSt | incest | incest | 6 | 40% |
| CTRL | TIS | NSt | kogel | bullet | 6 | 40% |
| CTRL | TIS | NSt | kreng | bitch | 6 | 40% |
| CTRL | NIS | NSt | kuthoer | pussy whore | 6 | 40% |
| CTRL | TIS | NSt | ontrouw | unfaithful | 6 | 40% |
| CTRL | TIS | NSt | pedofiel | paedophile | 6 | 40% |
| CTRL | TIS | NSt | sadist | sadist | 6 | 40% |
| CTRL | TIS | NSt | verdrinken | to drown | 6 | 40% |
| CTRL | NIS | NSt | verzuipen | to drown | 6 | 40% |
| CTRL | NIS | NSn | cirkel | circle | 6 | 40% |
| CTRL | TIS | NSn | elleboog | elbow | 6 | 40% |
| CTRL | NIS | NSn | etiket | label | 6 | 40% |
| CTRL | NIS | NSn | firma | firm | 6 | 40% |
| CTRL | TIS | NSn | firma | firm | 6 | 40% |
| CTRL | TIS | NSn | flacon | bottle or vial | 6 | 40% |
| CTRL | NIS | NSn | gaatje | little hole | 6 | 40% |
| CTRL | TIS | NSn | geit | goat | 6 | 40% |
| CTRL | TIS | NSn | hertogin | duchess | 6 | 40% |
| CTRL | TIS | NSn | kast | closet | 6 | 40% |
| CTRL | NIS | NSn | kenteken | license plate | 6 | 40% |
| CTRL | TIS | NSn | paragraaf | paragraph | 6 | 40% |
| CTRL | NIS | NSn | plafond | ceiling | 6 | 40% |
| CTRL | NIS | NSn | rad | wheel | 6 | 40% |
| CTRL | TIS | NSn | rad | wheel | 6 | 40% |
| CTRL | TIS | NSn | schroef | screw | 6 | 40% |
| CTRL | NIS | NSn | stellen | to set | 6 | 40% |
| CTRL | TIS | NSn | teller | counter | 6 | 40% |
| CTRL | NIS | NSn | theelepel | teaspoon | 6 | 40% |
| CTRL | TIS | NSn | tijdperk | era | 6 | 40% |
| CTRL | TIS | NSn | vierkant | square | 6 | 40% |
| CTRL | TIS | NSn | zandloper | hourglass | 6 | 40% |
| CTRL | NIS | NSn | zegel | seal | 6 | 40% |
| CTRL | NIS | NSn | zetel | seat | 6 | 40% |
| CTRL | TIS | St | achterlaten | leave behind | 5 | 33% |
| CTRL | NIS | St | afscheid | goodbye | 5 | 33% |
| CTRL | TIS | St | afwijzing | rejection | 5 | 33% |
| CTRL | TIS | St | alleen | alone | 5 | 33% |
| CTRL | NIS | St | angst | fear | 5 | 33% |
| CTRL | TIS | St | bedrieger | deceiver | 5 | 33% |
| CTRL | TIS | St | boos | angry | 5 | 33% |
| CTRL | NIS | St | conflict | conflict | 5 | 33% |
| CTRL | TIS | St | depressie | depression | 5 | 33% |
| CTRL | NIS | St | doodgaan | to die | 5 | 33% |
| CTRL | NIS | St | droefheid | sadness | 5 | 33% |
| CTRL | NIS | St | droevig | sad | 5 | 33% |
| CTRL | TIS | St | kanker | cancer | 5 | 33% |
| CTRL | NIS | St | lawaai | noise | 5 | 33% |
| CTRL | TIS | St | lawaai | noise | 5 | 33% |
| CTRL | TIS | St | moedeloos | despondent | 5 | 33% |
| CTRL | TIS | St | pedofiel | paedophile | 5 | 33% |
| CTRL | TIS | St | schok | shock | 5 | 33% |
| CTRL | TIS | St | schoppen | to kick | 5 | 33% |
| CTRL | TIS | St | tegenslag | setback | 5 | 33% |
| CTRL | TIS | St | wanhoop | despair | 5 | 33% |
| CTRL | TIS | NSt | afblaffen | to bark at | 5 | 33% |
| CTRL | TIS | NSt | afgunst | envy | 5 | 33% |
| CTRL | TIS | NSt | bedrieger | deceiver | 5 | 33% |
| CTRL | TIS | NSt | inbraak | burglary | 5 | 33% |
| CTRL | TIS | NSt | lafaard | coward | 5 | 33% |
| CTRL | TIS | NSt | liegen | to lie | 5 | 33% |
| CTRL | NIS | NSt | miskraam | miscarriage | 5 | 33% |
| CTRL | TIS | NSt | slaaf | slave | 5 | 33% |
| CTRL | NIS | NSt | steekwond | stab wound | 5 | 33% |
| CTRL | TIS | NSt | tiran | tyrant | 5 | 33% |
| CTRL | NIS | NSt | trauma | trauma | 5 | 33% |
| CTRL | NIS | NSt | verlammen | to paralyze | 5 | 33% |
| CTRL | TIS | NSn | bordeel | brothel | 5 | 33% |
| CTRL | TIS | NSn | chauffeur | driver | 5 | 33% |
| CTRL | NIS | NSn | citaat | quote | 5 | 33% |
| CTRL | TIS | NSn | dakgoot | gutter | 5 | 33% |
| CTRL | NIS | NSn | dwerg | dwarf | 5 | 33% |
| CTRL | NIS | NSn | embryo | embryo | 5 | 33% |
| CTRL | NIS | NSn | gebouw | building | 5 | 33% |
| CTRL | NIS | NSn | geit | goat | 5 | 33% |
| CTRL | TIS | NSn | handen | hands | 5 | 33% |
| CTRL | NIS | NSn | kast | closet | 5 | 33% |
| CTRL | NIS | NSn | kelder | basement | 5 | 33% |
| CTRL | NIS | NSn | kozijn | window frame | 5 | 33% |
| CTRL | NIS | NSn | laden | to load | 5 | 33% |
| CTRL | TIS | NSn | meester | master | 5 | 33% |
| CTRL | NIS | NSn | metaal | metal | 5 | 33% |
| CTRL | NIS | NSn | middel | middle | 5 | 33% |
| CTRL | TIS | NSn | plafond | ceiling | 5 | 33% |
| CTRL | TIS | NSn | plank | shelf | 5 | 33% |
| CTRL | NIS | NSn | potlood | pencil | 5 | 33% |
| CTRL | TIS | NSn | register | register | 5 | 33% |
| CTRL | NIS | NSn | roeren | to stir | 5 | 33% |
| CTRL | TIS | NSn | schuren | to polish | 5 | 33% |
| CTRL | NIS | NSn | spoelen | to flush or to rinse | 5 | 33% |
| CTRL | NIS | NSn | tapijt | tapestry | 5 | 33% |
| CTRL | TIS | NSn | tapijt | tapestry | 5 | 33% |
| CTRL | TIS | NSn | theelepel | teaspoon | 5 | 33% |
| CTRL | TIS | NSn | touw | rope | 5 | 33% |
| CTRL | TIS | NSn | traject | route | 5 | 33% |
| CTRL | NIS | NSn | trede | step | 5 | 33% |
| CTRL | TIS | NSn | trede | step | 5 | 33% |
| CTRL | NIS | NSn | treden | to step | 5 | 33% |
| CTRL | NIS | NSn | trottoir | pavement | 5 | 33% |
| CTRL | TIS | NSn | uitgever | publisher | 5 | 33% |
| CTRL | TIS | NSn | verleidster | temptress | 5 | 33% |
| CTRL | TIS | NSn | zetel | seat | 5 | 33% |
| CTRL | TIS | St | afsnauwen | to snap at | 4 | 27% |
| CTRL | NIS | St | bang | afraid | 4 | 27% |
| CTRL | TIS | St | begraven | to bury | 4 | 27% |
| CTRL | NIS | St | boos | angry | 4 | 27% |
| CTRL | TIS | St | broer | brother | 4 | 27% |
| CTRL | TIS | St | conflict | conflict | 4 | 27% |
| CTRL | NIS | St | depressie | depression | 4 | 27% |
| CTRL | TIS | St | doden | to kill | 4 | 27% |
| CTRL | TIS | St | droevig | sad | 4 | 27% |
| CTRL | TIS | St | falen | to fail | 4 | 27% |
| CTRL | TIS | St | gezwel | tumor | 4 | 27% |
| CTRL | NIS | St | krabben | to scratch | 4 | 27% |
| CTRL | TIS | St | krenken | to hurt | 4 | 27% |
| CTRL | NIS | St | kwetsen | to hurt | 4 | 27% |
| CTRL | TIS | St | leugen | lie | 4 | 27% |
| CTRL | TIS | St | miskraam | miscarriage | 4 | 27% |
| CTRL | TIS | St | misleiden | to deceive | 4 | 27% |
| CTRL | TIS | St | noodkreet | cry for help | 4 | 27% |
| CTRL | TIS | St | ongeluk | accident | 4 | 27% |
| CTRL | TIS | St | ontrouw | unfaithful | 4 | 27% |
| CTRL | NIS | St | pijn | pain | 4 | 27% |
| CTRL | TIS | St | sadist | sadist | 4 | 27% |
| CTRL | TIS | St | snauwen | to snarl | 4 | 27% |
| CTRL | TIS | St | stiekem | secretly | 4 | 27% |
| CTRL | TIS | St | stikken | to suffocate | 4 | 27% |
| CTRL | TIS | St | tiran | tyrant | 4 | 27% |
| CTRL | NIS | St | trauma | trauma | 4 | 27% |
| CTRL | TIS | St | verlammen | to paralyze | 4 | 27% |
| CTRL | TIS | St | vernielen | to vandalize | 4 | 27% |
| CTRL | TIS | St | verzuipen | to drown | 4 | 27% |
| CTRL | NIS | St | wanhopen | to despair | 4 | 27% |
| CTRL | TIS | St | wreedheid | cruelty | 4 | 27% |
| CTRL | NIS | NSt | agressie | aggression | 4 | 27% |
| CTRL | NIS | NSt | armoede | poverty | 4 | 27% |
| CTRL | TIS | NSt | armoede | poverty | 4 | 27% |
| CTRL | NIS | NSt | bedreigen | to threaten | 4 | 27% |
| CTRL | NIS | NSt | doodgaan | to die | 4 | 27% |
| CTRL | NIS | NSt | dwang | coercion or force | 4 | 27% |
| CTRL | TIS | NSt | getreiter | harassment | 4 | 27% |
| CTRL | NIS | NSt | gezwel | tumor | 4 | 27% |
| CTRL | TIS | NSt | gezwel | tumor | 4 | 27% |
| CTRL | TIS | NSt | hoer | whore | 4 | 27% |
| CTRL | NIS | NSt | kanker | cancer | 4 | 27% |
| CTRL | NIS | NSt | kogel | bullet | 4 | 27% |
| CTRL | TIS | NSt | kotsen | to vomit | 4 | 27% |
| CTRL | NIS | NSt | liegen | to lie | 4 | 27% |
| CTRL | NIS | NSt | lijden | to suffer | 4 | 27% |
| CTRL | TIS | NSt | miskraam | miscarriage | 4 | 27% |
| CTRL | TIS | NSt | mismaakt | deformed | 4 | 27% |
| CTRL | TIS | NSt | omkomen | to perish | 4 | 27% |
| CTRL | TIS | NSt | ontslag | resignation or dismissal | 4 | 27% |
| CTRL | NIS | NSt | oplichten | to scam | 4 | 27% |
| CTRL | NIS | NSt | opsluiten | to lock up | 4 | 27% |
| CTRL | TIS | NSt | orgie | orgy | 4 | 27% |
| CTRL | TIS | NSt | razernij | fury | 4 | 27% |
| CTRL | NIS | NSt | snauwen | to snarl | 4 | 27% |
| CTRL | TIS | NSt | stinken | to stink | 4 | 27% |
| CTRL | TIS | NSt | treiteren | to harass | 4 | 27% |
| CTRL | TIS | NSt | uitlachen | to laugh at | 4 | 27% |
| CTRL | TIS | NSt | vastbinden | to tie | 4 | 27% |
| CTRL | NIS | NSt | wanhoop | despair | 4 | 27% |
| CTRL | NIS | NSn | absorptie | absorption | 4 | 27% |
| CTRL | TIS | NSn | absorptie | absorption | 4 | 27% |
| CTRL | NIS | NSn | blikje | can | 4 | 27% |
| CTRL | TIS | NSn | citaat | quote | 4 | 27% |
| CTRL | NIS | NSn | deurknop | door knob | 4 | 27% |
| CTRL | NIS | NSn | doorslikken | to swallow | 4 | 27% |
| CTRL | NIS | NSn | dubbel | double | 4 | 27% |
| CTRL | NIS | NSn | elleboog | elbow | 4 | 27% |
| CTRL | NIS | NSn | haken | hooks | 4 | 27% |
| CTRL | TIS | NSn | haken | hooks | 4 | 27% |
| CTRL | NIS | NSn | hertogin | duchess | 4 | 27% |
| CTRL | NIS | NSn | ivoor | ivory | 4 | 27% |
| CTRL | NIS | NSn | kader | framework | 4 | 27% |
| CTRL | TIS | NSn | laden | to load | 4 | 27% |
| CTRL | TIS | NSn | likken | to lick | 4 | 27% |
| CTRL | NIS | NSn | meester | master | 4 | 27% |
| CTRL | TIS | NSn | middel | middle | 4 | 27% |
| CTRL | NIS | NSn | octaaf | octave | 4 | 27% |
| CTRL | TIS | NSn | octaaf | octave | 4 | 27% |
| CTRL | TIS | NSn | orgie | orgy | 4 | 27% |
| CTRL | NIS | NSn | paragraaf | paragraph | 4 | 27% |
| CTRL | TIS | NSn | pasen | Easter | 4 | 27% |
| CTRL | TIS | NSn | pilaar | pillar | 4 | 27% |
| CTRL | NIS | NSn | regenton | rain barrel | 4 | 27% |
| CTRL | NIS | NSn | takken | branches | 4 | 27% |
| CTRL | TIS | NSn | takken | branches | 4 | 27% |
| CTRL | TIS | NSn | trottoir | pavement | 4 | 27% |
| CTRL | TIS | NSn | uitkleden | to undress | 4 | 27% |
| CTRL | TIS | NSn | verbranden | to burn | 4 | 27% |
| CTRL | NIS | NSn | vreemde | stranger | 4 | 27% |
| CTRL | TIS | St | afblaffen | to bark at | 3 | 20% |
| CTRL | NIS | St | afkraken | to decry | 3 | 20% |
| CTRL | NIS | St | alleen | alone | 3 | 20% |
| CTRL | TIS | St | bedrog | deceit | 3 | 20% |
| CTRL | NIS | St | benauwen | to agitate | 3 | 20% |
| CTRL | NIS | St | beroerte | stroke | 3 | 20% |
| CTRL | NIS | St | besmetten | to contaminate | 3 | 20% |
| CTRL | TIS | St | doodgaan | to die | 3 | 20% |
| CTRL | TIS | St | dubbel | double | 3 | 20% |
| CTRL | NIS | St | dwingen | to coerce or to force | 3 | 20% |
| CTRL | TIS | St | gijzeling | kidnapping | 3 | 20% |
| CTRL | NIS | St | inbraak | burglary | 3 | 20% |
| CTRL | TIS | St | knijpen | to pinch | 3 | 20% |
| CTRL | NIS | St | kotsen | to vomit | 3 | 20% |
| CTRL | NIS | St | krenken | to hurt | 3 | 20% |
| CTRL | TIS | St | liegen | to lie | 3 | 20% |
| CTRL | NIS | St | lijden | to suffer | 3 | 20% |
| CTRL | TIS | St | mes | knife | 3 | 20% |
| CTRL | TIS | St | nacht | night | 3 | 20% |
| CTRL | TIS | St | ongeval | accident | 3 | 20% |
| CTRL | NIS | St | onrecht | injustice | 3 | 20% |
| CTRL | TIS | St | razernij | fury | 3 | 20% |
| CTRL | NIS | St | ruzie | fight or quarrel | 3 | 20% |
| CTRL | TIS | St | schoft | bastard | 3 | 20% |
| CTRL | NIS | St | schok | shock | 3 | 20% |
| CTRL | NIS | St | schuldig | guilty | 3 | 20% |
| CTRL | NIS | St | snauwen | to snarl | 3 | 20% |
| CTRL | NIS | St | sterven | to die | 3 | 20% |
| CTRL | TIS | St | uitgescholden | scolded | 3 | 20% |
| CTRL | TIS | St | uitlachen | to laugh at | 3 | 20% |
| CTRL | TIS | St | vader | father | 3 | 20% |
| CTRL | NIS | NSt | afkraken | to decry | 3 | 20% |
| CTRL | NIS | NSt | afsnauwen | to snap at | 3 | 20% |
| CTRL | TIS | NSt | agressie | aggression | 3 | 20% |
| CTRL | NIS | NSt | belazerd | fooled | 3 | 20% |
| CTRL | TIS | NSt | bordeel | brothel | 3 | 20% |
| CTRL | TIS | NSt | domkop | idiot | 3 | 20% |
| CTRL | NIS | NSt | dwingen | to coerce or to force | 3 | 20% |
| CTRL | TIS | NSt | dwingen | to coerce or to force | 3 | 20% |
| CTRL | NIS | NSt | hoer | whore | 3 | 20% |
| CTRL | NIS | NSt | hysterie | hysteria | 3 | 20% |
| CTRL | NIS | NSt | kotsen | to vomit | 3 | 20% |
| CTRL | NIS | NSt | krenken | to hurt | 3 | 20% |
| CTRL | TIS | NSt | kuthoer | pussy whore | 3 | 20% |
| CTRL | TIS | NSt | misdaad | crime | 3 | 20% |
| CTRL | TIS | NSt | misdrijf | crime | 3 | 20% |
| CTRL | TIS | NSt | misleiden | to deceive | 3 | 20% |
| CTRL | NIS | NSt | mismaakt | deformed | 3 | 20% |
| CTRL | NIS | NSt | ongeluk | accident | 3 | 20% |
| CTRL | TIS | NSt | ongeval | accident | 3 | 20% |
| CTRL | TIS | NSt | opsluiten | to lock up | 3 | 20% |
| CTRL | NIS | NSt | pijn | pain | 3 | 20% |
| CTRL | NIS | NSt | ruzie | fight or quarrel | 3 | 20% |
| CTRL | NIS | NSt | schoft | bastard | 3 | 20% |
| CTRL | NIS | NSt | schoppen | to kick | 3 | 20% |
| CTRL | NIS | NSt | slaaf | slave | 3 | 20% |
| CTRL | TIS | NSt | slecht | bad | 3 | 20% |
| CTRL | TIS | NSt | slijmen | suck up to | 3 | 20% |
| CTRL | TIS | NSt | snijden | to cut | 3 | 20% |
| CTRL | TIS | NSt | stank | stench | 3 | 20% |
| CTRL | NIS | NSt | sterven | to die | 3 | 20% |
| CTRL | TIS | NSt | stikken | to suffocate | 3 | 20% |
| CTRL | TIS | NSt | verdord | withered | 3 | 20% |
| CTRL | TIS | NSt | verkrachting | rape | 3 | 20% |
| CTRL | TIS | NSt | verlammen | to paralyze | 3 | 20% |
| CTRL | TIS | NSt | vies | dirty | 3 | 20% |
| CTRL | NIS | NSt | walging | disgust | 3 | 20% |
| CTRL | TIS | NSt | walging | disgust | 3 | 20% |
| CTRL | TIS | NSt | wreedheid | cruelty | 3 | 20% |
| CTRL | TIS | NSt | zondebok | scapegoat | 3 | 20% |
| CTRL | NIS | NSn | aanmaken | to prepare | 3 | 20% |
| CTRL | TIS | NSn | aanmaken | to prepare | 3 | 20% |
| CTRL | TIS | NSn | afhakken | to chop off | 3 | 20% |
| CTRL | TIS | NSn | atoombom | nuclear bomb | 3 | 20% |
| CTRL | TIS | NSn | besmetten | to contaminate | 3 | 20% |
| CTRL | NIS | NSn | bladzijde | page | 3 | 20% |
| CTRL | TIS | NSn | bladzijde | page | 3 | 20% |
| CTRL | TIS | NSn | blikje | can | 3 | 20% |
| CTRL | NIS | NSn | blinddoek | blindfold | 3 | 20% |
| CTRL | TIS | NSn | blinddoek | blindfold | 3 | 20% |
| CTRL | NIS | NSn | bloot | naked | 3 | 20% |
| CTRL | NIS | NSn | branden | to burn | 3 | 20% |
| CTRL | TIS | NSn | cirkel | circle | 3 | 20% |
| CTRL | TIS | NSn | deurknop | door knob | 3 | 20% |
| CTRL | NIS | NSn | dozijn | dozen | 3 | 20% |
| CTRL | TIS | NSn | dozijn | dozen | 3 | 20% |
| CTRL | TIS | NSn | dwerg | dwarf | 3 | 20% |
| CTRL | TIS | NSn | element | element | 3 | 20% |
| CTRL | TIS | NSn | etter | pus | 3 | 20% |
| CTRL | TIS | NSn | gaatje | little hole | 3 | 20% |
| CTRL | TIS | NSn | kelder | basement | 3 | 20% |
| CTRL | TIS | NSn | knijpen | to pinch | 3 | 20% |
| CTRL | NIS | NSn | kreunen | to moan | 3 | 20% |
| CTRL | NIS | NSn | legpuzzel | jigsaw puzzle | 3 | 20% |
| CTRL | NIS | NSn | likken | to lick | 3 | 20% |
| CTRL | TIS | NSn | magazijn | warehouse | 3 | 20% |
| CTRL | NIS | NSn | mes | knife | 3 | 20% |
| CTRL | TIS | NSn | metaal | metal | 3 | 20% |
| CTRL | NIS | NSn | nacht | night | 3 | 20% |
| CTRL | NIS | NSn | piemel | willy | 3 | 20% |
| CTRL | NIS | NSn | pilaar | pillar | 3 | 20% |
| CTRL | NIS | NSn | postzegel | stamp | 3 | 20% |
| CTRL | NIS | NSn | snijden | to cut | 3 | 20% |
| CTRL | TIS | NSn | spoelen | to flush or to rinse | 3 | 20% |
| CTRL | TIS | NSn | steil | steep | 3 | 20% |
| CTRL | NIS | NSn | stoel | chair | 3 | 20% |
| CTRL | NIS | NSn | stoelpoot | chair leg | 3 | 20% |
| CTRL | NIS | NSn | stoep | sidewalk | 3 | 20% |
| CTRL | TIS | NSn | stoep | sidewalk | 3 | 20% |
| CTRL | TIS | NSn | verdord | withered | 3 | 20% |
| CTRL | NIS | NSn | verleidster | temptress | 3 | 20% |
| CTRL | TIS | NSn | vreemde | stranger | 3 | 20% |
| CTRL | TIS | NSn | vuur | fire | 3 | 20% |
| CTRL | NIS | NSn | zuigen | to suck | 3 | 20% |
| CTRL | TIS | NSn | zuigen | to suck | 3 | 20% |
| CTRL | NIS | St | afblaffen | to bark at | 2 | 13% |
| CTRL | TIS | St | afkeer | aversion | 2 | 13% |
| CTRL | NIS | St | afsnauwen | to snap at | 2 | 13% |
| CTRL | TIS | St | armoede | poverty | 2 | 13% |
| CTRL | NIS | St | bedrog | deceit | 2 | 13% |
| CTRL | NIS | St | begraven | to bury | 2 | 13% |
| CTRL | NIS | St | buurman | neighbor | 2 | 13% |
| CTRL | TIS | St | chanteren | to blackmail | 2 | 13% |
| CTRL | NIS | St | diefstal | theft | 2 | 13% |
| CTRL | NIS | St | domkop | idiot | 2 | 13% |
| CTRL | TIS | St | doodsteek | deathblow | 2 | 13% |
| CTRL | NIS | St | dwang | coercion or force | 2 | 13% |
| CTRL | TIS | St | etiket | label | 2 | 13% |
| CTRL | TIS | St | getreiter | harassment | 2 | 13% |
| CTRL | TIS | St | haten | to hate | 2 | 13% |
| CTRL | TIS | St | hijgen | to pant | 2 | 13% |
| CTRL | TIS | St | hoer | whore | 2 | 13% |
| CTRL | TIS | St | hysterie | hysteria | 2 | 13% |
| CTRL | TIS | St | inbraak | burglary | 2 | 13% |
| CTRL | TIS | St | incest | incest | 2 | 13% |
| CTRL | NIS | St | kreng | bitch | 2 | 13% |
| CTRL | TIS | St | kreunen | to moan | 2 | 13% |
| CTRL | NIS | St | leugen | lie | 2 | 13% |
| CTRL | NIS | St | liegen | to lie | 2 | 13% |
| CTRL | TIS | St | martelen | to torture | 2 | 13% |
| CTRL | TIS | St | masker | mask | 2 | 13% |
| CTRL | TIS | St | messteek | knife stab | 2 | 13% |
| CTRL | TIS | St | misdaad | crime | 2 | 13% |
| CTRL | NIS | St | miskraam | miscarriage | 2 | 13% |
| CTRL | TIS | St | mislukt | failed | 2 | 13% |
| CTRL | TIS | St | moord | murder | 2 | 13% |
| CTRL | NIS | St | omkomen | to perish | 2 | 13% |
| CTRL | NIS | St | ongewenst | unwanted | 2 | 13% |
| CTRL | NIS | St | ontslag | resignation or dismissal | 2 | 13% |
| CTRL | TIS | St | ontslag | resignation or dismissal | 2 | 13% |
| CTRL | TIS | St | oom | uncle | 2 | 13% |
| CTRL | TIS | St | opsluiten | to lock up | 2 | 13% |
| CTRL | TIS | St | piemel | willy | 2 | 13% |
| CTRL | TIS | St | schande | shame | 2 | 13% |
| CTRL | TIS | St | slet | slut | 2 | 13% |
| CTRL | TIS | St | sterven | to die | 2 | 13% |
| CTRL | TIS | St | trappen | to kick | 2 | 13% |
| CTRL | TIS | St | treiteren | to harass | 2 | 13% |
| CTRL | NIS | St | uitlachen | to laugh at | 2 | 13% |
| CTRL | TIS | St | vastbinden | to tie | 2 | 13% |
| CTRL | TIS | St | verdrinken | to drown | 2 | 13% |
| CTRL | NIS | St | verzuipen | to drown | 2 | 13% |
| CTRL | TIS | St | vies | dirty | 2 | 13% |
| CTRL | TIS | St | walging | disgust | 2 | 13% |
| CTRL | NIS | St | wanhoop | despair | 2 | 13% |
| CTRL | TIS | St | wraak | revenge | 2 | 13% |
| CTRL | TIS | St | wurgen | to strangle | 2 | 13% |
| CTRL | NIS | St | zeer | ache | 2 | 13% |
| CTRL | TIS | St | zondebok | scapegoat | 2 | 13% |
| CTRL | NIS | NSt | afhakken | to chop off | 2 | 13% |
| CTRL | TIS | NSt | afhakken | to chop off | 2 | 13% |
| CTRL | NIS | NSt | afkeer | aversion | 2 | 13% |
| CTRL | TIS | NSt | afschuw | revulsion | 2 | 13% |
| CTRL | TIS | NSt | afsnauwen | to snap at | 2 | 13% |
| CTRL | NIS | NSt | afwijzing | rejection | 2 | 13% |
| CTRL | NIS | NSt | bang | afraid | 2 | 13% |
| CTRL | NIS | NSt | bedrog | deceit | 2 | 13% |
| CTRL | NIS | NSt | begraven | to bury | 2 | 13% |
| CTRL | NIS | NSt | besmetten | to contaminate | 2 | 13% |
| CTRL | TIS | NSt | blinddoek | blindfold | 2 | 13% |
| CTRL | TIS | NSt | boos | angry | 2 | 13% |
| CTRL | TIS | NSt | depressie | depression | 2 | 13% |
| CTRL | TIS | NSt | doodgaan | to die | 2 | 13% |
| CTRL | NIS | NSt | etter | pus | 2 | 13% |
| CTRL | TIS | NSt | etter | pus | 2 | 13% |
| CTRL | NIS | NSt | getreiter | harassment | 2 | 13% |
| CTRL | NIS | NSt | inbraak | burglary | 2 | 13% |
| CTRL | NIS | NSt | instorten | to collapse | 2 | 13% |
| CTRL | NIS | NSt | kreng | bitch | 2 | 13% |
| CTRL | NIS | NSt | kwetsen | to hurt | 2 | 13% |
| CTRL | TIS | NSt | leugen | lie | 2 | 13% |
| CTRL | TIS | NSt | meeloper | opportunist | 2 | 13% |
| CTRL | NIS | NSt | mislukt | failed | 2 | 13% |
| CTRL | TIS | NSt | misvormen | to deform | 2 | 13% |
| CTRL | NIS | NSt | onrecht | injustice | 2 | 13% |
| CTRL | TIS | NSt | onrecht | injustice | 2 | 13% |
| CTRL | NIS | NSt | schaden | to damage | 2 | 13% |
| CTRL | TIS | NSt | schande | shame | 2 | 13% |
| CTRL | TIS | NSt | schoft | bastard | 2 | 13% |
| CTRL | TIS | NSt | schoppen | to kick | 2 | 13% |
| CTRL | NIS | NSt | slecht | bad | 2 | 13% |
| CTRL | TIS | NSt | sterven | to die | 2 | 13% |
| CTRL | NIS | NSt | stinken | to stink | 2 | 13% |
| CTRL | TIS | NSt | uitgescholden | scolded | 2 | 13% |
| CTRL | NIS | NSt | uitjouwen | to boo | 2 | 13% |
| CTRL | TIS | NSt | uitjouwen | to boo | 2 | 13% |
| CTRL | TIS | NSt | verleidster | temptress | 2 | 13% |
| CTRL | TIS | NSt | vernielen | to vandalize | 2 | 13% |
| CTRL | TIS | NSt | wanhoop | despair | 2 | 13% |
| CTRL | NIS | NSt | woest | enraged | 2 | 13% |
| CTRL | TIS | NSt | woest | enraged | 2 | 13% |
| CTRL | TIS | NSn | achterlaten | leave behind | 2 | 13% |
| CTRL | NIS | NSn | atoombom | nuclear bomb | 2 | 13% |
| CTRL | TIS | NSn | bad | bath | 2 | 13% |
| CTRL | NIS | NSn | begraven | to bury | 2 | 13% |
| CTRL | TIS | NSn | bloot | naked | 2 | 13% |
| CTRL | NIS | NSn | broer | brother | 2 | 13% |
| CTRL | TIS | NSn | buurman | neighbor | 2 | 13% |
| CTRL | NIS | NSn | chauffeur | driver | 2 | 13% |
| CTRL | NIS | NSn | domkop | idiot | 2 | 13% |
| CTRL | TIS | NSn | domkop | idiot | 2 | 13% |
| CTRL | TIS | NSn | embryo | embryo | 2 | 13% |
| CTRL | TIS | NSn | folteren | to torture | 2 | 13% |
| CTRL | NIS | NSn | hagedis | lizard | 2 | 13% |
| CTRL | TIS | NSn | hagedis | lizard | 2 | 13% |
| CTRL | NIS | NSn | handen | hands | 2 | 13% |
| CTRL | NIS | NSn | hijgen | to pant | 2 | 13% |
| CTRL | TIS | NSn | hijgen | to pant | 2 | 13% |
| CTRL | TIS | NSn | juni | June | 2 | 13% |
| CTRL | TIS | NSn | kader | framework | 2 | 13% |
| CTRL | NIS | NSn | kogel | bullet | 2 | 13% |
| CTRL | TIS | NSn | kozijn | window frame | 2 | 13% |
| CTRL | TIS | NSn | krabben | to scratch | 2 | 13% |
| CTRL | TIS | NSn | martelen | to torture | 2 | 13% |
| CTRL | TIS | NSn | mes | knife | 2 | 13% |
| CTRL | NIS | NSn | misvormen | to deform | 2 | 13% |
| CTRL | TIS | NSn | misvormen | to deform | 2 | 13% |
| CTRL | TIS | NSn | moord | murder | 2 | 13% |
| CTRL | TIS | NSn | ogen | eyes | 2 | 13% |
| CTRL | NIS | NSn | ontslag | resignation or dismissal | 2 | 13% |
| CTRL | NIS | NSn | oom | uncle | 2 | 13% |
| CTRL | TIS | NSn | opsluiten | to lock up | 2 | 13% |
| CTRL | NIS | NSn | orgie | orgy | 2 | 13% |
| CTRL | NIS | NSn | pasen | Easter | 2 | 13% |
| CTRL | TIS | NSn | piemel | willy | 2 | 13% |
| CTRL | TIS | NSn | regel | rule | 2 | 13% |
| CTRL | TIS | NSn | schoft | bastard | 2 | 13% |
| CTRL | TIS | NSn | stank | stench | 2 | 13% |
| CTRL | NIS | NSn | steil | steep | 2 | 13% |
| CTRL | TIS | NSn | stoel | chair | 2 | 13% |
| CTRL | TIS | NSn | stoelpoot | chair leg | 2 | 13% |
| CTRL | NIS | NSn | trappen | to kick | 2 | 13% |
| CTRL | TIS | NSn | treden | to step | 2 | 13% |
| CTRL | TIS | NSn | vergroten | enlarge | 2 | 13% |
| CTRL | TIS | NSn | verlammen | to paralyze | 2 | 13% |
| CTRL | NIS | NSn | vuur | fire | 2 | 13% |
| CTRL | NIS | St | achterlaten | leave behind | 1 | 7% |
| CTRL | TIS | St | afgrijzen | horror | 1 | 7% |
| CTRL | NIS | St | afgunst | envy | 1 | 7% |
| CTRL | TIS | St | afgunst | envy | 1 | 7% |
| CTRL | NIS | St | afkeer | aversion | 1 | 7% |
| CTRL | TIS | St | afschuw | revulsion | 1 | 7% |
| CTRL | NIS | St | agressie | aggression | 1 | 7% |
| CTRL | NIS | St | armoede | poverty | 1 | 7% |
| CTRL | TIS | St | baby | baby | 1 | 7% |
| CTRL | NIS | St | bedrieger | deceiver | 1 | 7% |
| CTRL | NIS | St | belazerd | fooled | 1 | 7% |
| CTRL | TIS | St | bestek | cutlery | 1 | 7% |
| CTRL | TIS | St | buurman | neighbor | 1 | 7% |
| CTRL | NIS | St | dakgoot | gutter | 1 | 7% |
| CTRL | TIS | St | deurknop | door knob | 1 | 7% |
| CTRL | TIS | St | document | document | 1 | 7% |
| CTRL | TIS | St | dokter | (general practice) doctor | 1 | 7% |
| CTRL | TIS | St | domkop | idiot | 1 | 7% |
| CTRL | TIS | St | doodslag | manslaughter | 1 | 7% |
| CTRL | NIS | St | dubbel | double | 1 | 7% |
| CTRL | NIS | St | ellende | misery | 1 | 7% |
| CTRL | TIS | St | ergeren | to annoy | 1 | 7% |
| CTRL | TIS | St | ergernis | annoyance | 1 | 7% |
| CTRL | TIS | St | folteren | to torture | 1 | 7% |
| CTRL | NIS | St | hagedis | lizard | 1 | 7% |
| CTRL | NIS | St | hijgen | to pant | 1 | 7% |
| CTRL | NIS | St | huisdier | pet | 1 | 7% |
| CTRL | TIS | St | huisdier | pet | 1 | 7% |
| CTRL | NIS | St | ijzer | iron | 1 | 7% |
| CTRL | NIS | St | instorten | to collapse | 1 | 7% |
| CTRL | TIS | St | juni | June | 1 | 7% |
| CTRL | NIS | St | kelder | basement | 1 | 7% |
| CTRL | TIS | St | kelder | basement | 1 | 7% |
| CTRL | NIS | St | kerk | church | 1 | 7% |
| CTRL | TIS | St | kerk | church | 1 | 7% |
| CTRL | NIS | St | knijpen | to pinch | 1 | 7% |
| CTRL | TIS | St | kogel | bullet | 1 | 7% |
| CTRL | TIS | St | kotsen | to vomit | 1 | 7% |
| CTRL | TIS | St | kou | cold | 1 | 7% |
| CTRL | TIS | St | kreng | bitch | 1 | 7% |
| CTRL | TIS | St | kuthoer | pussy whore | 1 | 7% |
| CTRL | NIS | St | lafaard | coward | 1 | 7% |
| CTRL | TIS | St | man | man | 1 | 7% |
| CTRL | NIS | St | masker | mask | 1 | 7% |
| CTRL | NIS | St | meeloper | opportunist | 1 | 7% |
| CTRL | TIS | St | meeloper | opportunist | 1 | 7% |
| CTRL | NIS | St | mes | knife | 1 | 7% |
| CTRL | NIS | St | metaal | metal | 1 | 7% |
| CTRL | NIS | St | moedeloos | despondent | 1 | 7% |
| CTRL | TIS | St | moeder | mother | 1 | 7% |
| CTRL | TIS | St | mond | mouth | 1 | 7% |
| CTRL | NIS | St | ogen | eyes | 1 | 7% |
| CTRL | TIS | St | ogen | eyes | 1 | 7% |
| CTRL | NIS | St | ongeluk | accident | 1 | 7% |
| CTRL | NIS | St | ongeval | accident | 1 | 7% |
| CTRL | NIS | St | ontrouw | unfaithful | 1 | 7% |
| CTRL | TIS | St | oorlog | war | 1 | 7% |
| CTRL | NIS | St | oplichten | to scam | 1 | 7% |
| CTRL | NIS | St | opsluiten | to lock up | 1 | 7% |
| CTRL | NIS | St | pasen | Easter | 1 | 7% |
| CTRL | TIS | St | plafond | ceiling | 1 | 7% |
| CTRL | NIS | St | razernij | fury | 1 | 7% |
| CTRL | NIS | St | schande | shame | 1 | 7% |
| CTRL | TIS | St | slaaf | slave | 1 | 7% |
| CTRL | TIS | St | slecht | bad | 1 | 7% |
| CTRL | TIS | St | slijmen | suck up to | 1 | 7% |
| CTRL | TIS | St | spoelen | to flush or to rinse | 1 | 7% |
| CTRL | NIS | St | stank | stench | 1 | 7% |
| CTRL | TIS | St | stank | stench | 1 | 7% |
| CTRL | NIS | St | steekwond | stab wound | 1 | 7% |
| CTRL | TIS | St | steekwond | stab wound | 1 | 7% |
| CTRL | NIS | St | steil | steep | 1 | 7% |
| CTRL | NIS | St | stiekem | secretly | 1 | 7% |
| CTRL | NIS | St | stinken | to stink | 1 | 7% |
| CTRL | NIS | St | stomerij | drycleaner | 1 | 7% |
| CTRL | TIS | St | touw | rope | 1 | 7% |
| CTRL | NIS | St | treiteren | to harass | 1 | 7% |
| CTRL | NIS | St | uitgescholden | scolded | 1 | 7% |
| CTRL | TIS | St | uitjouwen | to boo | 1 | 7% |
| CTRL | NIS | St | vader | father | 1 | 7% |
| CTRL | NIS | St | verbranden | to burn | 1 | 7% |
| CTRL | NIS | St | verdrinken | to drown | 1 | 7% |
| CTRL | NIS | St | verkrachting | rape | 1 | 7% |
| CTRL | TIS | St | verleidster | temptress | 1 | 7% |
| CTRL | NIS | St | vies | dirty | 1 | 7% |
| CTRL | NIS | St | vreemde | stranger | 1 | 7% |
| CTRL | TIS | St | zetel | seat | 1 | 7% |
| CTRL | TIS | St | zeuren | to nag | 1 | 7% |
| CTRL | NIS | St | zuigen | to suck | 1 | 7% |
| CTRL | TIS | St | zuigen | to suck | 1 | 7% |
| CTRL | NIS | NSt | achterlaten | leave behind | 1 | 7% |
| CTRL | TIS | NSt | achterlaten | leave behind | 1 | 7% |
| CTRL | NIS | NSt | afblaffen | to bark at | 1 | 7% |
| CTRL | TIS | NSt | afgrijzen | horror | 1 | 7% |
| CTRL | TIS | NSt | afkraken | to decry | 1 | 7% |
| CTRL | NIS | NSt | afschuw | revulsion | 1 | 7% |
| CTRL | TIS | NSt | afwijzing | rejection | 1 | 7% |
| CTRL | NIS | NSt | alleen | alone | 1 | 7% |
| CTRL | NIS | NSt | angst | fear | 1 | 7% |
| CTRL | TIS | NSt | bang | afraid | 1 | 7% |
| CTRL | TIS | NSt | bedrog | deceit | 1 | 7% |
| CTRL | TIS | NSt | begraven | to bury | 1 | 7% |
| CTRL | TIS | NSt | belazerd | fooled | 1 | 7% |
| CTRL | TIS | NSt | besmetten | to contaminate | 1 | 7% |
| CTRL | NIS | NSt | boos | angry | 1 | 7% |
| CTRL | NIS | NSt | bordeel | brothel | 1 | 7% |
| CTRL | NIS | NSt | branden | to burn | 1 | 7% |
| CTRL | NIS | NSt | conflict | conflict | 1 | 7% |
| CTRL | NIS | NSt | depressie | depression | 1 | 7% |
| CTRL | NIS | NSt | diefstal | theft | 1 | 7% |
| CTRL | NIS | NSt | domkop | idiot | 1 | 7% |
| CTRL | NIS | NSt | dreigen | to threaten | 1 | 7% |
| CTRL | TIS | NSt | dreigen | to threaten | 1 | 7% |
| CTRL | TIS | NSt | dwang | coercion or force | 1 | 7% |
| CTRL | NIS | NSt | ergeren | to annoy | 1 | 7% |
| CTRL | TIS | NSt | ergeren | to annoy | 1 | 7% |
| CTRL | NIS | NSt | ergernis | annoyance | 1 | 7% |
| CTRL | TIS | NSt | ergernis | annoyance | 1 | 7% |
| CTRL | TIS | NSt | falen | to fail | 1 | 7% |
| CTRL | TIS | NSt | geweld | violence | 1 | 7% |
| CTRL | TIS | NSt | hijgen | to pant | 1 | 7% |
| CTRL | TIS | NSt | instorten | to collapse | 1 | 7% |
| CTRL | NIS | NSt | knijpen | to pinch | 1 | 7% |
| CTRL | TIS | NSt | knijpen | to pinch | 1 | 7% |
| CTRL | TIS | NSt | kou | cold | 1 | 7% |
| CTRL | TIS | NSt | krabben | to scratch | 1 | 7% |
| CTRL | NIS | NSt | mes | knife | 1 | 7% |
| CTRL | TIS | NSt | middel | middle | 1 | 7% |
| CTRL | TIS | NSt | mislukt | failed | 1 | 7% |
| CTRL | NIS | NSt | misvormen | to deform | 1 | 7% |
| CTRL | TIS | NSt | moedeloos | despondent | 1 | 7% |
| CTRL | NIS | NSt | noodkreet | cry for help | 1 | 7% |
| CTRL | TIS | NSt | noodkreet | cry for help | 1 | 7% |
| CTRL | TIS | NSt | ongewenst | unwanted | 1 | 7% |
| CTRL | NIS | NSt | onmacht | powerlessness | 1 | 7% |
| CTRL | NIS | NSt | ontslag | resignation or dismissal | 1 | 7% |
| CTRL | NIS | NSt | orgie | orgy | 1 | 7% |
| CTRL | TIS | NSt | pijn | pain | 1 | 7% |
| CTRL | TIS | NSt | ruzie | fight or quarrel | 1 | 7% |
| CTRL | TIS | NSt | schaden | to damage | 1 | 7% |
| CTRL | NIS | NSt | schok | shock | 1 | 7% |
| CTRL | NIS | NSt | stank | stench | 1 | 7% |
| CTRL | NIS | NSt | steil | steep | 1 | 7% |
| CTRL | NIS | NSt | tegenslag | setback | 1 | 7% |
| CTRL | TIS | NSt | tegenslag | setback | 1 | 7% |
| CTRL | TIS | NSt | telegram | telegram | 1 | 7% |
| CTRL | NIS | NSt | trappen | to kick | 1 | 7% |
| CTRL | NIS | NSt | uitgescholden | scolded | 1 | 7% |
| CTRL | NIS | NSt | uitlachen | to laugh at | 1 | 7% |
| CTRL | NIS | NSt | vastbinden | to tie | 1 | 7% |
| CTRL | NIS | NSt | verbranden | to burn | 1 | 7% |
| CTRL | TIS | NSt | verbranden | to burn | 1 | 7% |
| CTRL | NIS | NSt | verdord | withered | 1 | 7% |
| CTRL | NIS | NSt | verleidster | temptress | 1 | 7% |
| CTRL | NIS | NSt | vernielen | to vandalize | 1 | 7% |
| CTRL | TIS | NSt | verraad | betrayal | 1 | 7% |
| CTRL | TIS | NSt | vierkant | square | 1 | 7% |
| CTRL | TIS | NSt | wanhopen | to despair | 1 | 7% |
| CTRL | NIS | NSt | wraak | revenge | 1 | 7% |
| CTRL | TIS | NSt | zetel | seat | 1 | 7% |
| CTRL | NIS | NSt | zondebok | scapegoat | 1 | 7% |
| CTRL | NIS | NSn | aanslag | attack | 1 | 7% |
| CTRL | TIS | NSn | aanslag | attack | 1 | 7% |
| CTRL | TIS | NSn | afblaffen | to bark at | 1 | 7% |
| CTRL | NIS | NSn | afhakken | to chop off | 1 | 7% |
| CTRL | NIS | NSn | afkeer | aversion | 1 | 7% |
| CTRL | NIS | NSn | afschuw | revulsion | 1 | 7% |
| CTRL | TIS | NSn | afschuw | revulsion | 1 | 7% |
| CTRL | NIS | NSn | armoede | poverty | 1 | 7% |
| CTRL | TIS | NSn | armoede | poverty | 1 | 7% |
| CTRL | NIS | NSn | bad | bath | 1 | 7% |
| CTRL | NIS | NSn | bang | afraid | 1 | 7% |
| CTRL | NIS | NSn | bed | bed | 1 | 7% |
| CTRL | NIS | NSn | bedrog | deceit | 1 | 7% |
| CTRL | TIS | NSn | begraven | to bury | 1 | 7% |
| CTRL | TIS | NSn | beklemmen | to oppress | 1 | 7% |
| CTRL | NIS | NSn | bordeel | brothel | 1 | 7% |
| CTRL | TIS | NSn | branden | to burn | 1 | 7% |
| CTRL | NIS | NSn | buurman | neighbor | 1 | 7% |
| CTRL | TIS | NSn | chanteren | to blackmail | 1 | 7% |
| CTRL | NIS | NSn | conflict | conflict | 1 | 7% |
| CTRL | TIS | NSn | crimineel | criminal | 1 | 7% |
| CTRL | NIS | NSn | depressie | depression | 1 | 7% |
| CTRL | TIS | NSn | diefstal | theft | 1 | 7% |
| CTRL | NIS | NSn | document | document | 1 | 7% |
| CTRL | TIS | NSn | document | document | 1 | 7% |
| CTRL | NIS | NSn | dokter | (general practice) doctor | 1 | 7% |
| CTRL | TIS | NSn | dokter | (general practice) doctor | 1 | 7% |
| CTRL | TIS | NSn | doodsteek | deathblow | 1 | 7% |
| CTRL | TIS | NSn | doorslikken | to swallow | 1 | 7% |
| CTRL | NIS | NSn | droevig | sad | 1 | 7% |
| CTRL | TIS | NSn | dubbel | double | 1 | 7% |
| CTRL | TIS | NSn | dwang | coercion or force | 1 | 7% |
| CTRL | NIS | NSn | ergernis | annoyance | 1 | 7% |
| CTRL | TIS | NSn | ergernis | annoyance | 1 | 7% |
| CTRL | TIS | NSn | etiket | label | 1 | 7% |
| CTRL | NIS | NSn | etter | pus | 1 | 7% |
| CTRL | NIS | NSn | folteren | to torture | 1 | 7% |
| CTRL | TIS | NSn | gebouw | building | 1 | 7% |
| CTRL | TIS | NSn | gezwel | tumor | 1 | 7% |
| CTRL | TIS | NSn | gijzeling | kidnapping | 1 | 7% |
| CTRL | TIS | NSn | hysterie | hysteria | 1 | 7% |
| CTRL | TIS | NSn | inbraak | burglary | 1 | 7% |
| CTRL | NIS | NSn | incest | incest | 1 | 7% |
| CTRL | TIS | NSn | incest | incest | 1 | 7% |
| CTRL | NIS | NSn | juni | June | 1 | 7% |
| CTRL | NIS | NSn | knijpen | to pinch | 1 | 7% |
| CTRL | TIS | NSn | kogel | bullet | 1 | 7% |
| CTRL | NIS | NSn | kou | cold | 1 | 7% |
| CTRL | NIS | NSn | krabben | to scratch | 1 | 7% |
| CTRL | TIS | NSn | krenken | to hurt | 1 | 7% |
| CTRL | TIS | NSn | kreunen | to moan | 1 | 7% |
| CTRL | NIS | NSn | lafaard | coward | 1 | 7% |
| CTRL | TIS | NSn | lafaard | coward | 1 | 7% |
| CTRL | TIS | NSn | legpuzzel | jigsaw puzzle | 1 | 7% |
| CTRL | TIS | NSn | lepra | leprosy | 1 | 7% |
| CTRL | NIS | NSn | magazijn | warehouse | 1 | 7% |
| CTRL | NIS | NSn | masker | mask | 1 | 7% |
| CTRL | NIS | NSn | meeloper | opportunist | 1 | 7% |
| CTRL | NIS | NSn | miskraam | miscarriage | 1 | 7% |
| CTRL | NIS | NSn | mislukt | failed | 1 | 7% |
| CTRL | TIS | NSn | mislukt | failed | 1 | 7% |
| CTRL | NIS | NSn | mismaakt | deformed | 1 | 7% |
| CTRL | TIS | NSn | mismaakt | deformed | 1 | 7% |
| CTRL | TIS | NSn | moeder | mother | 1 | 7% |
| CTRL | NIS | NSn | mond | mouth | 1 | 7% |
| CTRL | NIS | NSn | noodkreet | cry for help | 1 | 7% |
| CTRL | TIS | NSn | noodkreet | cry for help | 1 | 7% |
| CTRL | NIS | NSn | ogen | eyes | 1 | 7% |
| CTRL | NIS | NSn | ongeval | accident | 1 | 7% |
| CTRL | TIS | NSn | ongeval | accident | 1 | 7% |
| CTRL | NIS | NSn | onmacht | powerlessness | 1 | 7% |
| CTRL | NIS | NSn | opsluiten | to lock up | 1 | 7% |
| CTRL | NIS | NSn | pedofiel | paedophile | 1 | 7% |
| CTRL | TIS | NSn | pedofiel | paedophile | 1 | 7% |
| CTRL | NIS | NSn | regel | rule | 1 | 7% |
| CTRL | TIS | NSn | roofmoord | robbery with murder | 1 | 7% |
| CTRL | TIS | NSn | sadist | sadist | 1 | 7% |
| CTRL | TIS | NSn | schaden | to damage | 1 | 7% |
| CTRL | NIS | NSn | schande | shame | 1 | 7% |
| CTRL | TIS | NSn | schande | shame | 1 | 7% |
| CTRL | NIS | NSn | schok | shock | 1 | 7% |
| CTRL | TIS | NSn | schoppen | to kick | 1 | 7% |
| CTRL | NIS | NSn | schuldig | guilty | 1 | 7% |
| CTRL | NIS | NSn | seks | sex | 1 | 7% |
| CTRL | TIS | NSn | slecht | bad | 1 | 7% |
| CTRL | NIS | NSn | slet | slut | 1 | 7% |
| CTRL | NIS | NSn | stank | stench | 1 | 7% |
| CTRL | TIS | NSn | steekwond | stab wound | 1 | 7% |
| CTRL | TIS | NSn | sterven | to die | 1 | 7% |
| CTRL | NIS | NSn | stiekem | secretly | 1 | 7% |
| CTRL | TIS | NSn | stiekem | secretly | 1 | 7% |
| CTRL | TIS | NSn | stikken | to suffocate | 1 | 7% |
| CTRL | TIS | NSn | stinken | to stink | 1 | 7% |
| CTRL | TIS | NSn | trappen | to kick | 1 | 7% |
| CTRL | NIS | NSn | trauma | trauma | 1 | 7% |
| CTRL | NIS | NSn | vader | father | 1 | 7% |
| CTRL | NIS | NSn | vastbinden | to tie | 1 | 7% |
| CTRL | TIS | NSn | vastbinden | to tie | 1 | 7% |
| CTRL | NIS | NSn | verdord | withered | 1 | 7% |
| CTRL | NIS | NSn | vergroten | enlarge | 1 | 7% |
| CTRL | NIS | NSn | verraad | betrayal | 1 | 7% |
| CTRL | NIS | NSn | woest | enraged | 1 | 7% |
| CTRL | NIS | NSn | zeer | ache | 1 | 7% |

TrialType = word presented in the trial (in Dutch), eText = English translation of the word, n = number of participants who rated this word highly, prop = percentage of participants who rated highly this word out of total individuals in the Group, St = self-relevant trauma-related, NSt = non-self-relevant trauma-related, NSn = Non-self-relevant neutral, NIS = neutral identity state, TIS = trauma-related identity state, DID-G = individuals with a diagnosis of dissociative identity disorder (DID), that is genuine DID, DID-S = DID-simulating controls, CTRL = a paired control group of healthy participants (controls for the NIS) and individuals with a diagnosis of PTSD (controls for the TIS)

^n1 = 14

^n2 = 14

^n3 = 15

**REFERENCES**

Bernstein, E. M., & Putnam, F. W. (1986). Development, reliability, and validity of a dissociation scale. *Journal of Nervous and Mental Disease,* *174*(12), 727–735.

Frischholz, E. J., Braun, B. G., Sachs, R. G., Hopkins, L., Shaeffer, D. M., Lewis, J., Leavitt, F., Pasquotto, J. N., & Schwartz, D. R. (1990). The Dissociative Experiences Scale: Further replication and validation. *Dissociation*, *3*, 151–153.

Nijenhuis, E. R. S., Spinhoven, P., Dyck, R., Van der Hart, O., & Vanderlinden, J. (1998). Psychometric Characteristics of the Somatoform Dissociation Questionnaire: A Replication Study. *Psychotherapy and Psychosomatics*, *67*(1), 17–23. https://doi.org/https://doi.org/10.1159/000012254

Nijenhuis, E. R., Spinhoven, P., Van Dyck, R., Van der Hart, O., & Vanderlinden, J. (1996). The development and psychometric characteristics of the Somatoform Dissociation Questionnaire (SDQ-20). *The Journal of Nervous and Mental Disease*, *184*(11), 688–694.

Sar, V., Tutkun, H., Alyanak, B., Bakim, B., & Baral, lsln. (2000). Frequency of dissociative disorders among psychiatric outpatients in Turkey. *Comprehensive Psychiatry*, *41*(3), 216–222. https://doi.org/10.1016/S0010-440X(00)90050-6

Sierra, M., & Berrios, G. E. (2000). The Cambridge Depersonalisation Scale: a new instrument for the measurement of depersonalisation. *Psychiatry Research*, *93*(2), 153–164. https://doi.org/10.1016/S0165-1781(00)00100-1

Vissia, E. M., Giesen, M. E., Chalavi, S., Nijenhuis, E. R. S., Draijer, N., Brand, B. L., & Reinders, A. A. T. S. (2016). Supplementary material for the manuscript: Is it Trauma or Fantasy-based? Comparing Dissociative Identity Disorder, Posttraumatic Stress Disorder, Simulators, and Controls. *Acta Psychiatrica Scandinavica*, *134*(2), 1–27. https://onlinelibrary.wiley.com/action/downloadSupplement?doi=10.1111%2Facps.12590&file=acps12590-sup-0001-SupInfo.pdf
